# Benchmarking missing-values approaches for predictive models on health databases

**DOI:** 10.1093/gigascience/giac013

**Published:** 2022-04-15

**Authors:** Alexandre Perez-Lebel, Gaël Varoquaux, Marine Le Morvan, Julie Josse, Jean-Baptiste Poline

**Affiliations:** McConnell Brain Imaging Centre, The Neuro (Montreal Neurological Institute-Hospital), Faculty of Medicine, McGill University, 3801 University Street, Montreal, QC H3A 2B4, Canada; Inria Saclay – Île-de-France, Parietal team, 1 Rue Honoré d'Estienne d'Orves, 91120 Palaiseau, France; Mila - Quebec Artificial Intelligence Institute, 6666 Saint-Urbain Street, Montréal, QC H2S 3H1, Canada; McConnell Brain Imaging Centre, The Neuro (Montreal Neurological Institute-Hospital), Faculty of Medicine, McGill University, 3801 University Street, Montreal, QC H3A 2B4, Canada; Inria Saclay – Île-de-France, Parietal team, 1 Rue Honoré d'Estienne d'Orves, 91120 Palaiseau, France; Mila - Quebec Artificial Intelligence Institute, 6666 Saint-Urbain Street, Montréal, QC H2S 3H1, Canada; Inria Saclay – Île-de-France, Parietal team, 1 Rue Honoré d'Estienne d'Orves, 91120 Palaiseau, France; Inria Montpellier, Bâtiment 5, 860 Rue de St-Priest, 34090 Montpellier, France; IDESP Institut Desbrest d’Épidémiologie et de Santé Publique, Campus Santé, IURC, 641 avenue du Doyen Gaston Giraud, 34090 Montpellier, France; McConnell Brain Imaging Centre, The Neuro (Montreal Neurological Institute-Hospital), Faculty of Medicine, McGill University, 3801 University Street, Montreal, QC H3A 2B4, Canada

**Keywords:** missing values, machine learning, supervised learning, benchmark, imputation, multiple imputation, bagging

## Abstract

**Background:**

As databases grow larger, it becomes harder to fully control their collection, and they frequently come with missing values. These large databases are well suited to train machine learning models, e.g., for forecasting or to extract biomarkers in biomedical settings. Such predictive approaches can use discriminative—rather than generative—modeling and thus open the door to new missing-values strategies. Yet existing empirical evaluations of strategies to handle missing values have focused on inferential statistics.

**Results:**

Here we conduct a systematic benchmark of missing-values strategies in predictive models with a focus on large health databases: 4 electronic health record datasets, 1 population brain imaging database, 1 health survey, and 2 intensive care surveys. Using gradient-boosted trees, we compare native support for missing values with simple and state-of-the-art imputation prior to learning. We investigate prediction accuracy and computational time. For prediction after imputation, we find that adding an indicator to express which values have been imputed is important, suggesting that the data are missing not at random. Elaborate missing-values imputation can improve prediction compared to simple strategies but requires longer computational time on large data. Learning trees that model missing values—with missing incorporated attribute—leads to robust, fast, and well-performing predictive modeling.

**Conclusions:**

Native support for missing values in supervised machine learning predicts better than state-of-the-art imputation with much less computational cost. When using imputation, it is important to add indicator columns expressing which values have been imputed.

## Background: Missing Values in Databases

Missing values are pervasive in many application domains. This is particularly true on health data, where missing values arise for a multitude of reasons: 2 patients rarely follow the same medical path and undergo the exact same set of examinations; measurements are omitted because of lack of time or because the patient’s condition does not allow it; hospitals do not collect exactly the same information because of diverging practices and the use of different devices; and so forth. This problem is exacerbated when the data are aggregated across multiple sources or when each individual sample comprises many features. The more data there are, the more data are missing.

There is a rich and established statistical literature for the treatment of missing data [1, 2], which has so far been mostly focused on inferential purposes, i.e., estimating parameters of a probabilistic model with their confidence intervals. For such a problem, an important distinction between missing data mechanisms was introduced by Rubin [3]: missing completely at random (MCAR), where the probability of having missing data does not depend on the covariates; missing at random (MAR), where the probability of a missing value only depends on the observed values of other variables; and missing not at random (MNAR), which covers all other cases. MNAR corresponds to cases where the missingness carries information. For example, if heartbeat measures are not reported when the values are too low, it creates an MNAR situation. Most available methods for inference in the presence of missing values are only valid under the MAR assumption, including maximum likelihood approaches with the expectation maximization algorithm [4], as well as multiple imputation [5]. The latter is a 2-step approach where the data are first imputed multiple times to create multiple completed datasets, and then the analysis is performed on each imputed dataset separately before combining the results to take into account the uncertainty due to missing values.

Supervised learning to build models that best predict a response using covariates with missing values can lead to a different trade-off than inference models [6,7]. In health, such predictive models are central to building complex biomarkers or risk scores or to forecasting an epidemic, and they can even underlie causal inference for policy evaluation [8]. They are increasingly used on electronic health records [9–11], where the choice of strategy to handle missing values remains a challenge [12]. Indeed, unlike with inference, little work to date has focused on the systematic evaluation of supervised learning with missing values. Existing works focus on benchmarking imputation quality [13,14]—which, as our study points out, is a different goal than prediction quality—or only focus on imputation-based methods [15].

In practice, a number of options are commonly used to learn predictive models with missing values. The simplest one is to delete all observations containing missing values. However, leaving aside the possible biases that this practice may induce, it often leads to considerable loss of information in high and even moderate dimensions. Indeed, when there are many variables, it is common that only a few observations are completely observed.

To deal with arbitrary subsets of input features, the most common practice currently consists in first imputing the missing values and then learning a predictive model (e.g., regression or classification) on the completed data. The popularity of this approach is mainly due to its simplicity and ease of implementation. After imputation, off-the-shelf learners can be applied on the completed dataset. Recent theoretical results show that applying a supervised-learning regression on imputed data can asymptotically recover the optimal prediction function; however most imputation strategies, including the common imputation by the conditional expectation, create discontinuities in the regression function to learn [16].

A small number of machine learning models can natively handle missing values, in particular popular tree-based methods. Trees greedily partition the input space into subspaces in order to minimize a risk. This non-smooth optimization scheme enables them to be easily adapted to directly learn from incomplete data. Several adaptations of trees to missing values have been proposed (see [7] for a short review). Missing incorporated in attributes (MIA, [17]) is the most promising strategy [7], described in the Experiment section.

In this work, we benchmark the most popular methods for supervised learning with missing values on multiple large real-world health databases. In contrast to most simulations, real health databases combine a number of challenges: unknown data distributions (not necessarily Gaussian), uncontrolled missing data mechanism (not necessarily MAR), mixed quantitative and categorical data, and often a high level of noise. In such a challenging setting, we compare existing approaches to make recommendations that are directly relevant for the practitioner. To establish general recommendations, we study a total of 13 prediction real-world tasks (10 classification and 3 regression tasks) across 4 publicly available health databases of very different nature. For each of these tasks, we compare methodologies based on imputation followed by regression or classification with tree-based models that can natively handle missing values with a MIA strategy. These methods are chosen from the common practice as well as theoretical work on supervised learning with missing values [7].

The present study has several strengths in terms of benchmarking methodology, avoiding common limitations. It uses both real data and real missingness; multiple draws of cross-validation loops are used; the imputation procedure is not fitted on the whole dataset but rather on the training set to prevent leaks from the training set to the out-of-sample test set; hyperparameters of the predictive model are tuned for each method to reduce bias in the hyperparameter selection; and finally the study benchmarks both imputation methods and predictive models that handles missing values. As a result, our benchmark is very computation-intensive: the whole study cost ∼520 000 CPU hours, i.e., 60 years on a single CPU, revealing the need to also account for compute cost in recommendations.

After briefly exposing our benchmarking methodology, we give a synthetic view of the findings and discuss observed trends. Overall, the benchmarks reveal the presence of MNAR values and non-linear mechanisms. High-quality conditional imputation gives good prediction provided that a variable indicating which entries were imputed is added to the completed data. However, its algorithmic complexity makes it prohibitively costly on large data. Rather, tree-based methods with integrated support for missing values (MIA) perform as well or better, at a fraction of the computational cost.

## Empirical Study

### Benchmarking the imputation and MIA methods

Our experiments compare 2-step procedures based on imputation followed by regression or classification, as well as tree-based models with an intrinsic support for missing values thanks to MIA. The 12 methods compared are summarized in Table 1: MIA, 8 methods based on single imputation, and 3 methods using multiple imputation via bagging. Below, we describe further the imputation strategies benchmarked, as well as MIA.

**Table 1:** Methods compared in the main experiment

In-article name	Imputer	Mask	Bagging
MIA		No	No
Mean	Mean	No	No
Mean+mask	Mean	Yes	No
Median	Median	No	No
Median+mask	Median	Yes	No
Iterative	Iterative	No	No
Iterative+mask	Iterative	Yes	No
KNN	KNN	No	No
KNN+mask	KNN	Yes	No
Iterative+bagging	Iterative	No	Yes (100)
Iterative+mask+bagging	Iterative	Yes	Yes (100)
MIA+bagging		No	Yes (100)

All methods use gradient-boosted trees as predictive model; 10 use imputation and 2 use MIA. Bagging uses 100 estimators in the ensemble. KNN: *k*-nearest neighbors.

#### Single imputation

*Constant imputation: mean and median*.The simplest approach to imputation is to replace missing values by a constant such as the mean, the median, or the mode of the corresponding feature. This is frowned upon in classical statistical practice because the resulting data distribution is severely distorted compared to that of fully observed data. Yet, in a supervised setting, the goal is different from that of inferential tasks. Recent theoretical results have established that powerful learners such as those based on trees can learn to recognize such imputed values and give the best possible predictions [7]. The key to the success of this strategy is to impute the training and the test set with the same constant: missing values of the test set are imputed with the constants learned on the training set (e.g., mean, median).

*Conditional imputation: MICE and KNN*. Powerful imputation approaches rely on conditional dependencies between features to fill in the missing values. Adapting machine learning techniques gives flexible estimators of these dependencies. Classical approaches include *k*-nearest neighbor (KNN) regressors [18], and iterative conditional imputers that predict one feature as a function of others, as with the MICE imputer [19]. In our experiments, we benchmark their implementation in scikit-learn [20]: the KNNImputer as well as the IterativeImputer, using linear models to impute missing values.

##### Adding the mask

Conditional imputation can make it hard for the learner to retrieve which entries were originally observed and which were originally missing. However, the information of missingness can be relevant for predicting the outcome in cases where it depends on missingness, or in MNAR settings where the missingness carries information. For these reasons, it can be useful after imputation to add new binary features that encode whether a value was originally missing or not: the “mask” or “missingness indicator” [6,7,21].

#### Multiple imputation

When estimating model parameters, it is important to reflect the uncertainty due to the missing values. For this purpose, multiple imputation methods are widely used, often via resampling methods such as the bootstrap. However, for prediction (classification or regression) theoretical conditions differ from those of parameter estimation. Indeed, it has been shown recently that a sufficiently flexible learner reaches optimal performance asymptotically with single imputation, whatever the missing data mechanism and whatever the choice of imputation function [16]. Still, this result holds in asymptotic regimes, and there is a need for empirical results on handling missing values with multiple imputation or bootstrap in the context of supervised learning. Theoretically, the only result that we are aware of for multiple imputation in the context of prediction requires access to an oracle predictor for fully observed data and is valid only in MAR ([7], th. 3). In general, it is not clear how to use multiple imputation for supervised learning: sampling can be applied in different ways during training the model or applying their predictions to new data. Khan et al. [22] review and compare a number of methods for using multiple imputation and bootstrap: learning on an averaged version of a multiply imputed dataset, bagging single imputations, bagging multiple imputations, and constructing ensembles based on predictors that were each learned on a version of a multiply imputed dataset ([23], chap 16). Because these methods all come with a substantial computing cost, we focus on the most promising approach: bagging single imputation. More precisely, for each task we draw 100 bootstrap replicates. We then fit the single imputation and the predictive model on each of these replicates to obtain 100 predictors. Final predictions are made either by voting or by averaging (see Table A4).

#### Directly handling missing values with tree-based models: MIA

We also consider the MIA strategy to readily model missing values in tree-based models. It has the benefit of using all samples, including incomplete ones, to produce the splits of the input space. More precisely for each split based on variable *j*, all samples with a missing value in variable *j* are sent either to the left or to the right child node, depending on which option leads to the lowest risk. Note that the samples with an observed value in variable *j* can either be split between the left and right child node according to whether their valus *x_j_* is greater or smaller than a threshold, or all be sent to the same child node so that they are separated from the samples with a missing value in variable *j*. That makes MIA particularly suited to MNAR settings because it can harness the missingness information. Moreover, because trees with MIA directly learn with missing values, they provide a straightforward way of dealing with missing values in the test set. We use the implementation in scikit-learn’s boosted trees (HistGradientBoostingRegressor).

#### Predictive model

For the supervised learning step, we focus on gradient-boosted trees, although we also benchmark linear models in a complementary analysis described in the Appendices. We applied supervised learning to the imputed data for the imputation-based methods. We also used the tree models with their support of MIA for a direct handling of missing values. Gradient-boosted trees are state-of-the art predictors for tabular data [24–26] and thus constitute a strong baseline. Moreover, using gradient-boosted trees enables us to keep the same predictive model for all approaches, thereby putting emphasis on the effect of the missing data treatment.

To define the input features we either use the choice of experts in prior studies or feature screening, a classic machine learning procedure using a simple ANOVA-based univariate test of the link of each feature to the outcome [27]. In both cases, the same set of selected features is used for all methods within each predictive task. Selecting features is necessary because some of the imputation methods studied are not tractable with a large number of features.

### Health databases

To reach conclusions as general as possible we used 4 real-world health-related databases. These databases vary in terms of location, size, purpose, and time, to cover a wider data scope. These databases already existed and no data were collected for this study. Below, we describe them briefly, giving the prediction tasks studied for each.

#### Traumabase

Traumabase [28] is a collaboration studying major trauma. The database gathers information from 20 French trauma centers on >20,000 trauma cases from patient admission until discharge from critical care. Data collection started in 2010 and is still ongoing in 2020. We used records spanning 2010–2019. Data can be obtained by contacting the team on the Traumabase website [28].

We defined 5 prediction tasks on this database, 4 classifications and 1 regression. Outcomes are diverse: patient death, hemorrhagic shock, septic shock, and platelet count. Features for the hemorrhagic shock prediction are taken from Jiang et al. [29].

#### UK Biobank

UK Biobank (UKBB) [30] is a major prospective epidemiology cohort with biomedical measurements. It provides health information on >500,000 UK participants aged 40–69 years from 2006 to 2010. The data are available upon application as detailed on the UK BioBank website [30].

We defined 5 tasks on this database, 4 classifications and 1 regression. Outcomes are the diagnosis of 3 diseases—breast cancer, skin cancer, Parkinson disease—as well as prediction of the fluid intelligence score. Breast cancer prediction uses features defined in Läll et al. [31].

#### MIMIC-III

The Medical Information Mart for Intensive Care (MIMIC) database [32] is an intensive care unit (ICU) dataset developed by the MIT Lab for Computational Physiology. It comprises deidentified health data associated with ∼60,000 ICU admissions recorded at the Beth Israel Deaconess Medical Center of Boston, United States, between 2001 and 2012. It includes demographic characteristics, vital signs, laboratory test results, medications, and more. The data can be accessed via an application described on the MIMIC website [32].

We defined 2 classification tasks on this database. Outcomes are septic shock and hemorrhagic shock.

#### NHIS

The National Health Interview Survey (NHIS) [33] is a major data collection program of the National Center for Health Statistics (NCHS), part of the Centers for Disease Control and Prevention (CDC) in the United States. It aims to monitor the health of the population. Since 1957, it has been collecting data from the United States. We use the 2017 edition, summing up to ∼35,000 households containing ∼87,500 persons. The database is freely accessible on the NHIS website [33].

We defined 1 regression task on this database. Outcome is the yearly income.

More details on each database and task can be found in the Appendix, in particular in Table A6 and Fig. A3, which detail the number of features available and their type (numerical, ordinal, and categorical), and Fig. A5, which gives the distribution of missing values across features.

### Findings

**Figure 1.**
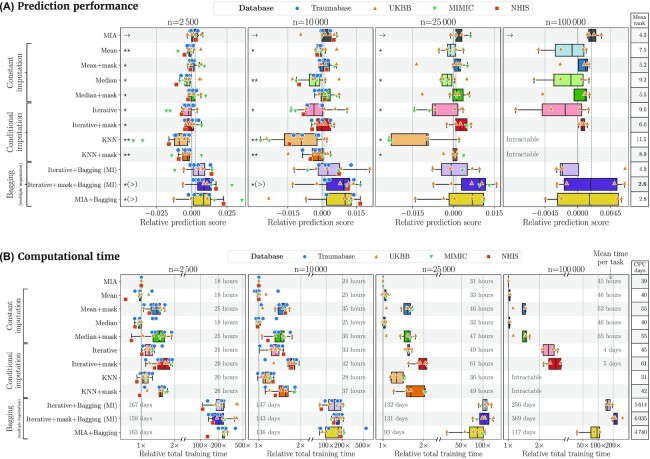
**Gradient-boosted trees models**. Comparison of prediction performance and training times across the 12 methods (see Table 1) for 13 prediction tasks spread over 4 databases, and for 4 sizes of dataset (2,500, 10,000, 25,000, and 100,000 samples). For each of the tasks and sizes, we computed a reference score by averaging the scores obtained by the 12 methods on the corresponding task and size. The relative prediction score of a method on a task and size is the deviation of the prediction score from the reference score of this task and size. For computational time, the total training time comprises imputation and tuning times and is given relative to that of MIA for each task and size. The box plots are composed of a box extending from first to third quartiles, a vertical line showing the median and left and right whiskers extending from the box to the last datum inside 1.5 times the interquartile range. More details on how these plots were created are given in the Plotting method section. The significance is assessed with a 1-sided Wilcoxon signed-rank test with MIA taken as reference (Table A3a). Methods that performed significantly poorer (respectively, better) at the 0.05 level are marked with “⋆” (“⋆( > )”) and “⋆⋆” (“⋆⋆( > )”) for Bonferroni-corrected levels. Two tables give the overall average ranks and the total number of CPU days for each method, all tasks and sizes combined. The mean number of CPU hours *per task* required to evaluate each method is given on each line. Detailed scores and ranks broken out by tasks are given in Table A7 and Fig. A6. Notice that KNN and KNN+mask were intractable at *n* = 100,000 due to their memory footprint of $\mathcal {O}(n^2)$.

Figure 1 summarizes the performance and computational times of the various methods across the 4 databases and 13 prediction tasks. To explore the importance of the amount of data, we created training datasets of 4 sizes: 2,500, 10,000, 25,000, and 100,000 samples. We report the general trends.

#### Bagging improves prediction, MIA performs well at limited cost

Iterative+mask+bagging obtains the best overall mean rank (2.6) across all tasks and sizes in terms of prediction score, closely followed by MIA+bagging (2.8) as shown on Fig. 1A and Table A7B. Overall, bagging improves all approaches markedly (Fig. A8). However the cost of these bagged methods can be prohibitive. At size *n* = 100,000, iterative+mask+bagging and MIA+bagging cost 369 and 117 CPU days per task, respectively, ∼100–200 times slower than a non-bagged method such as MIA (1.9 CPU days per task).

MIA makes it possible to navigate a trade-off between prediction performance and computational tractability: with bagging it comes close to iterative+mask with one-half the computational cost on large databases. Without bagging, it is the best overall performer, with an overall mean rank of 4.3, and up to 200 times faster. It is followed by mean+mask, median+mask and iterative+mask, with overall mean ranks of 5.2, 5.5, and 6.0, respectively. Mean, KNN+mask, iterative, median, and KNN performed the worst, with overall mean ranks of 7.5, 8.9, 9.0, 9.2, and 11.5, respectively. Table A7a and b give more quantitative details about scores and ranks of each method.

Similar observations can be made on each size separately. MIA obtained the best prediction scores on every size, with mean ranks of 4.3, 4.6, 4.4, and 2.5 on sizes 2,500, 10,000, 25,000, and 100,000, respectively, as shown in Fig. A2a.

In terms of computing time, beyond the fact that bagging multiplies the cost of every method by 100, MIA is almost always the fastest (Fig. 1B), although it gives excellent prediction performance. It is on par with mean and median imputations, but adding the mask to these methods—a key ingredient to prediction performance—doubles their computing times. At the other end of the spectrum, iterative+mask and KNN+mask are the slowest non-bagged methods. The gaps between training times of the methods increase with the size of the database, revealing the difference in algorithmic scalability.

##### Statistical significance

To assess significance of the above results, we ran 3 statistical tests: the Friedman test [34,35], the Nemenyi test [36], and the 1-sided Wilcoxon signed-rank test [37], all described in Demšar [38].

The Friedman test compares the mean ranks of several algorithms run on several datasets. The null hypothesis assumes that all algorithms are equivalent, i.e., their rank should be equal. Table A2 shows that the null hypothesis is rejected, with *P*-values much less than the 0.05 level for the sizes 2,500, 10,000, and 25,000. This indicates that ≥1 algorithm has significantly different performances from 1 other on these sizes. Following Demšar [38], we then proceed with a post hoc analysis with the Nemenyi test, assessing the significance of the difference between 2 algorithms using a critical difference. Algorithms with a difference in ranks smaller than the critical difference are not significantly different. Unfortunately, there are many methods to compare (12) comparatively to the number of datasets (13). As a result, the critical difference is high as shown in equation (3) and Table A2a and few comparisons are statistically significant when comparing the performance of MIA with the one of the other methods as shown on Fig. A2a. However, there are some significant results when comparing bagged methods. For example at size *n*= 2,500, iterative+mask+bagging and MIA+bagging performed significantly better than mean, median, iterative, KNN+mask, and KNN.

We run a complementary analysis with a 1-sided Wilcoxon signed-rank test, used for non-parametric tests comparing algorithms pairwise. We compare MIA with every other method. The null hypothesis claims that the median of the score differences between the 2 methods is positive (respectively, negative) for the 1-sided right (1-sided left) test. Results of the test are shown in Fig. 1A and Table A3b. At size *n*= 2,500, MIA performed significantly better than every other non-bagged method at the 0.05 level. MIA also performed significantly better than mean, KNN, and KNN+mask at the Bonferroni-corrected level. Bagged methods iterative+mask+bagging and MIA+bagging performed significantly better than MIA at the 0.05 level. The bigger the size *n*, the fewer tasks are available and so the less significant are the results.

#### Adding the mask improves prediction

Imputations with the additional variable representing the mask perform systematically better in terms of mean prediction score than their counterpart without mask (Fig. 1A, Table A7b).

In addition, MIA is not significantly better than the masked imputations, yet it is significantly better than the non-masked imputations (Fig. 1A, TableA3). However, adding the mask leads to longer training times (Fig. 1B). Indeed, adding the mask doubles the number of features for the supervised-learning step.

#### Conditional imputation is on par with constant imputation

Figure 1 shows that conditional imputation using iterative or KNN imputers does not perform consistently better than constant imputation. The overall mean rank of iterative and KNN are 9.0 and 11.5 versus 7.5 and 9.2 for mean and median, respectively (Fig. 1A and Table A7), and a similar delta is visible on the masked version.

#### Supplementary finding: Boosted trees outperform linear methods

**Figure A1.**
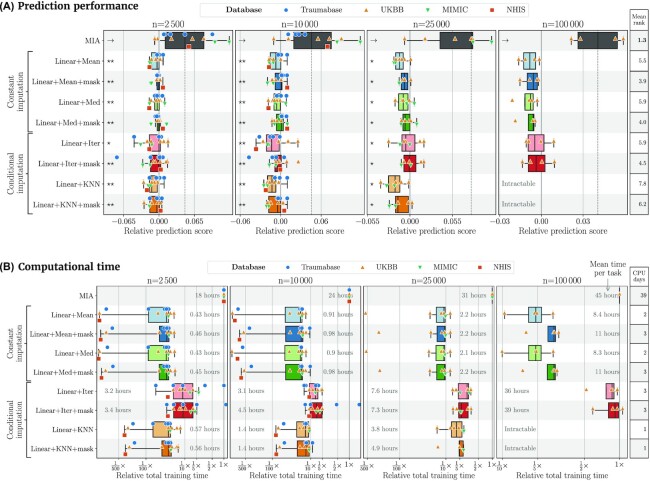
**Supplementary results: linear and gradient-boosted trees models**. Comparison of prediction performance and training times across the 9 methods(linear models and gradient boosting trees, see Table A1) for 13 prediction tasks spread over 4 databases, and for 4 sizes of dataset (2,500, 10,000, 25,000, and 100,000 samples). For linear models, ridge is used for regressions and logistic regression for classifications. For each of the tasks and sizes, we computed a reference score by averaging the scores obtained by the 9 methods on the corresponding task and size. The relative prediction score of a method on a task and size is the deviation of the prediction score from the reference score of this task and size. For computational time, the total training time comprises imputation and tuning times and is given relative to the one of MIA for each task and size. The box plots are composed of a box extending from first to third quartiles, a vertical line showing the median and left and right whiskers extending from the box to the last datum inside 1.5 times the interquartile range. More details on how these plots were created are given in the Detailed benchmarking methodology section. The significance is assessed with a 1-sided Wilcoxon signed-rank test with MIA taken as reference (see Table A3b). Methods which performed significantly poorer (resp. better) at the 0.05 level are marked with “⋆” (resp.“⋆(>)”) and “⋆⋆” (resp. “⋆⋆ (>)”) for Bonferroni-corrected levels. Two tables give the overall average ranks and the total number of CPU days for each method, all tasks and sizes combined. The average number of CPU hours per task required to evaluate each method is given on each line. Detailed scores and ranks broken out by tasks are given in Table A8. Notice that KNN and KNN+mask were intractable at n=100,000 due to their memory footprint of $\mathcal {O}(n^2)$.

**Figure A2.**
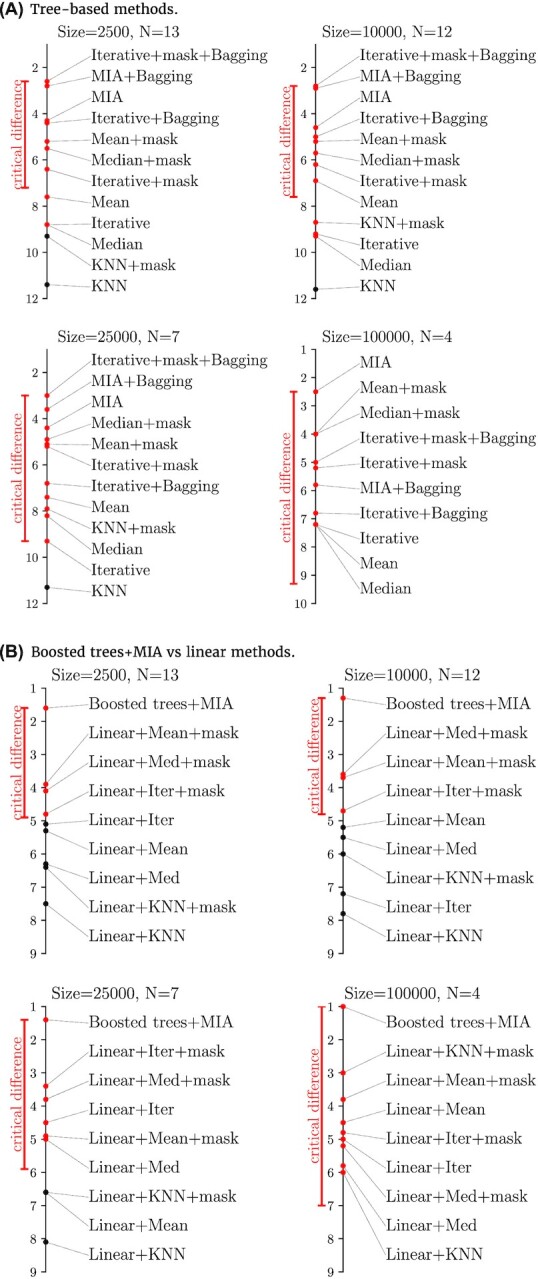
**Mean ranks by method and by size of dataset**. The critical difference is computed using the Nemenyi test (equation (3) and Table A2). Methods within the critical difference range do not perform significantly differently from one another according to the Nemenyi test. Methods within the critical difference range of MIA are in red, others in black.

Imputation methods paired with a linear model performed more poorly than when paired with boosted trees (Fig. A1, Table A8). Additionally, boosted trees paired with MIA are significantly better than every other method based on a linear model (TableA3).

## Discussion

### Interpretation

#### Model aggregation drives the good performance of multiple imputation

As with standard multiple-imputation strategies used for parameter estimation, bagging generates multiple bootstrap replicate training sets. Yet, the standard practice of multiple imputation strives to capture well the conditional distribution of the missing values given the observed one, while such conditional imputation is not needed for good prediction (as revealed by the good performance of MIA and [16]). Indeed, bagging in itself is known to improve generalization. To answer whether the good performance of multiple imputation can be attributed to ensembling (averaging multiple predictors) or capturing the conditional distribution, we performed an additional experiment with mean+mask+bagging (see Fig. A8). We observed that mean+mask+bagging is on par with iterative+mask+bagging, which suggests that the improved performances are rather due to the effect of bagging itself rather than capturing the conditional distribution of the missing data given the observed ones.

#### Good imputation does not imply good prediction, even for multiple imputation

It may be surprising at first that a sophisticated conditional imputation does not outperform constant imputation. Indeed, it contradicts the intuition that better imputation should lead to better prediction. Theoretical work shows that this intuition is not always true [16]: even in MAR settings, it may not hold for strongly non-linear mechanisms and little dependency across features. In the health databases that we studied, the features are weakly correlated: on average, only 12% of the features are correlated at >0.3 in absolute value (Table A5). This low correlation among features may explain our findings. If features are mostly independent, there is little information on the unobserved values to be extracted from the observed ones. For supervised learning, constant imputation comes with the benefit that it creates a simple structure captured by the supervised-learning step, which can then adapt to the missingness [7].

#### Boosted trees with MIA give best predictive models at little cost

MIA, the missing-values support inside gradient-boosted trees, appears as a method of choice to deal with missing values. Once put aside the prohibitively costly bagged methods, MIA was on average the best in terms of performance in our extensive benchmark while having a low computational cost. Sophisticated conditional imputation such as the iterative or KNN imputers are appealing because they may recover plausible values for missing entries, as discussed below. However, they are intractable with large datasets. Beyond the costs outlined by our experiments (Fig. 1B), the broader problem is the algorithmic scalability: for a dataset of *p* features and *n* samples, the compute cost of a KNN imputer scales as *n*^2^*p*^2^ and the memory footprint as *n*^2^, while the compute cost of an iterator imputer scales as *p*^2^*n*min (*n, p*) when it is based on linear models, the cheapest alternative. If both *p* and *n* grow, these costs rapidly become prohibitive. They prevented us from exploring larger datasets, e.g., with more features. Note that to ground valid predictions, the imputation model must be learned only on the training set; hence it is recomputed many times in a cross-validation loop.

**Figure A3.**
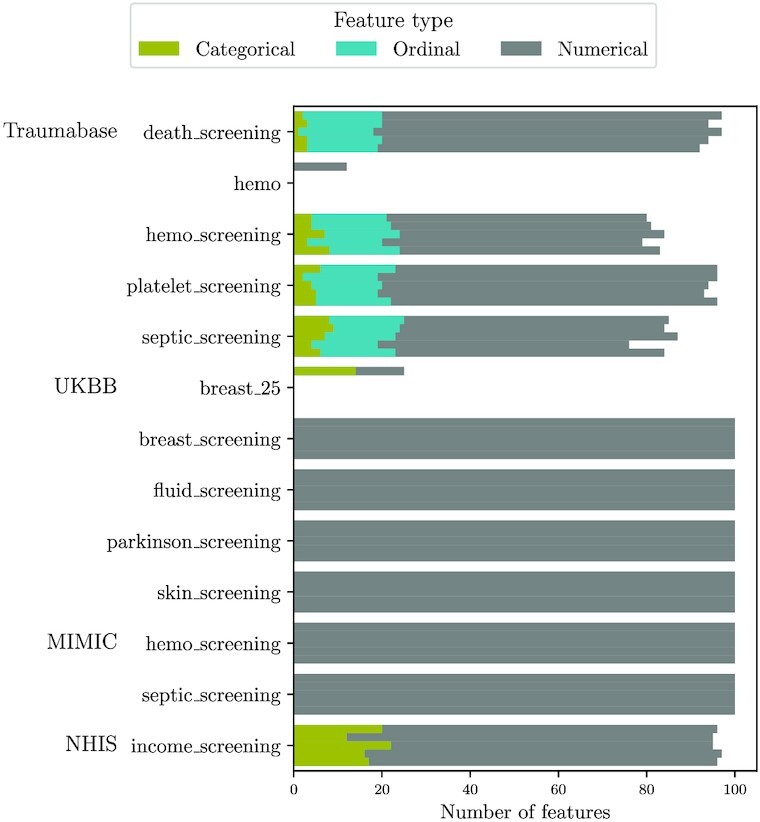
**Types of features**. Number of categorical, ordinal and numerical features in each dataset, before encoding. Note that one non-encoded categorical feature can lead to several selected encoded features. Since we select 100 encoded features, some task have less than 100 non-encoded features. For tasks having several trials, five horizontal bars are plotted representing one trial each, as feature selection may select different features.

Regardless of missing-values handling, gradient-boosted trees predict significantly better than linear models (Table A3). Tree-based models excel on categorical or ordinal features; however these are only a minority of the features of the databases studied (Fig. A3). Hence the good performance of gradient-boosted trees probably reveals non-linear mechanisms in the data. Note that the smallest database that we explored has a sample size of *n* = 2,500. For much smaller data, the simplest model—the linear model—may be the best choice.

#### The missingness is informative

For imputation-based pipelines, prediction significantly improves with the missingness mask added as input features. This suggests that the missingness is informative, which is often the case in health databases [39, 40]. Hence for all health databases studied, either the covariates are MNAR or the outcome to predict depends on the missingness. Either case falls outside of the theoretical framework that grounds the validity of statistical analysis using imputation [3,7]. The empirical results also confirm that the practice of adding the mask as input makes it possible to harness the predictive information in missing data patterns [6], which is otherwise hidden in the imputed data and much more difficult to recover.

#### Features with high missing rates are also important

**Figure A5.**
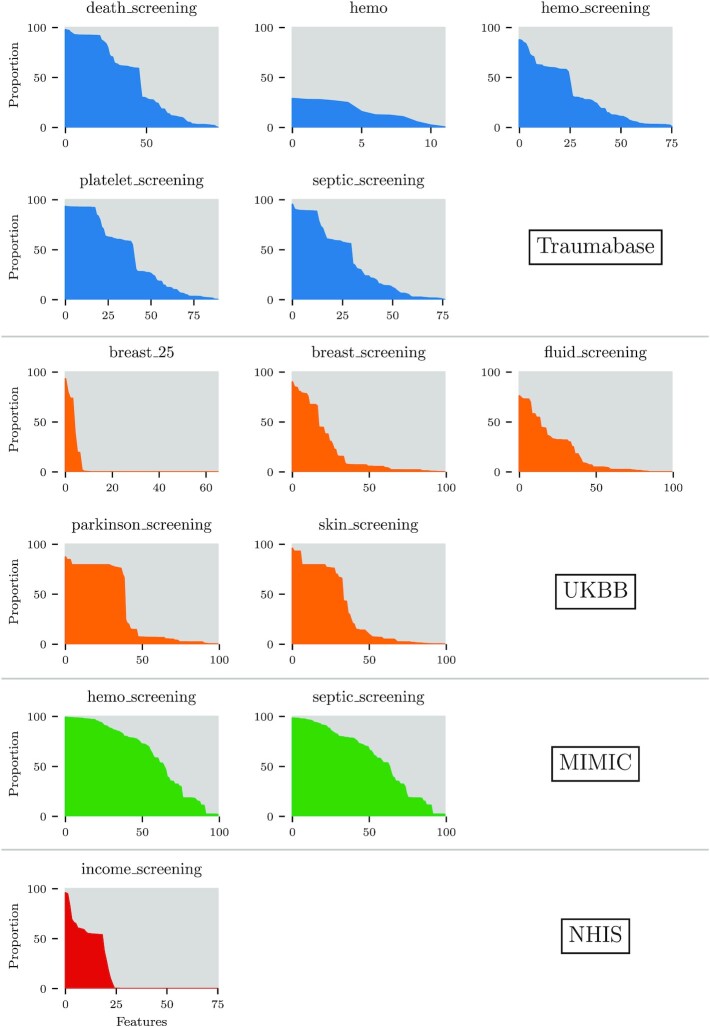
**Missing values distribution**. Proportion of missing values across selected encoded features for each task and for trial number 1, sorted in decreasing order. Other trials have similar proportions.

**Figure A7.**
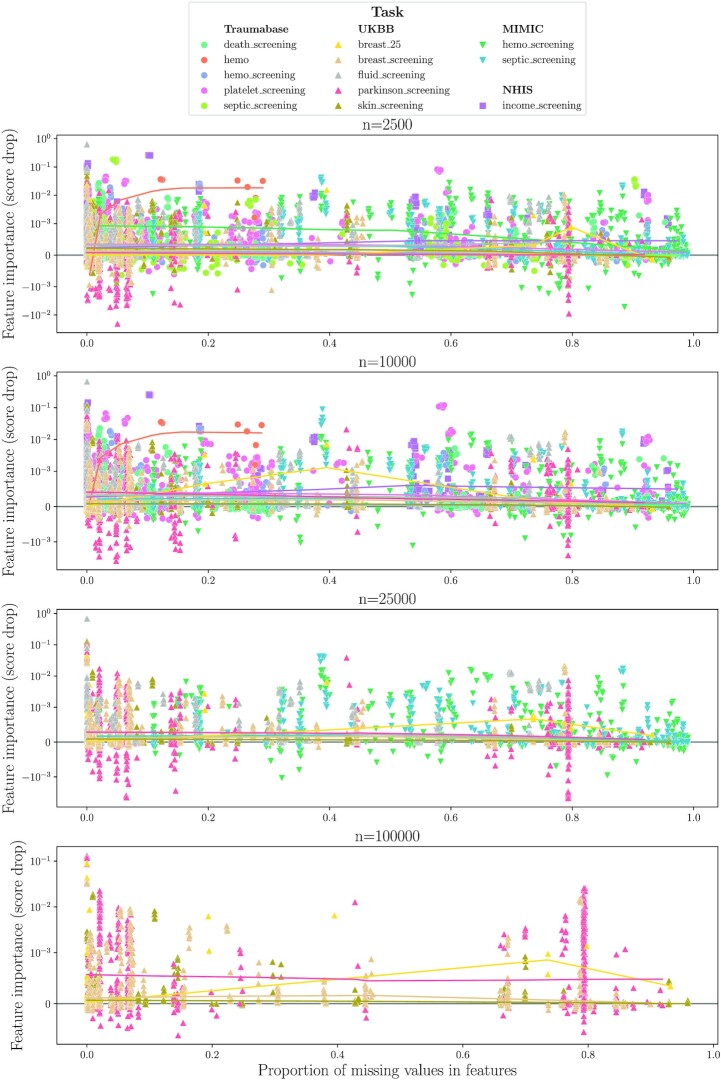
**Feature importance versus proportion of missing values**. Importance is measured as the drop in score when randomly permuting the considered feature. Each feature is permuted 10 times and its importance is taken as the average drop in score. Score drops are also averaged across folds. Local regressions (LOWESS) are plotted for each task to better visualize trends.

**Figure A8.**
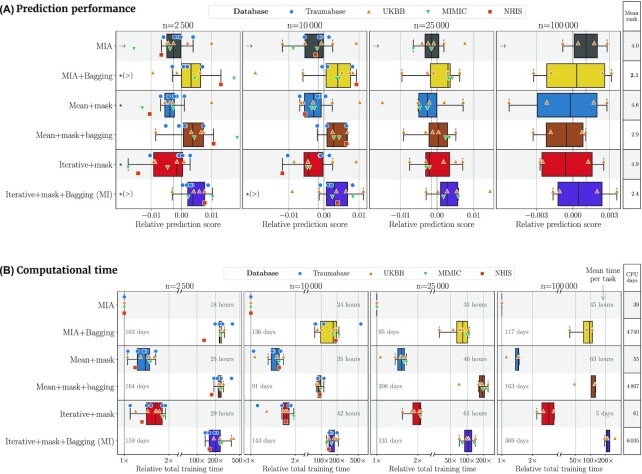
**Effect of bagging**. Comparison of prediction performance and training times between MIA, Mean+mask, Iterative+mask and their bagged version, for 13 prediction tasks spread over 4 databases, and for 4 sizes of dataset (2 500, 10 000, 25 000 and 100 000 samples). This figure is based on Figure 1, refer to caption of Figure 1 for more details.

Within each task, the missing rate per feature varies over a wide spectrum (see Fig. A5). We checked that features’ missing rates and predictive importance were not associated. For this, we measured permutation features: the drop in a model score after shuffling a feature, thereby canceling its contribution to the model performance. We ran this experiment for each task and each feature using scikit-learn’s implementation (see Table A4). We found no association between a feature’s missing rate and its importance (Fig. A7). Predictions do not only rely on features with few missing values. Moreover, even features with a very high level of missing values (e.g., >80%) seem to be as important as the others. This highlights the fact that it is worth making the effort of learning with incomplete features, even when they have a high missing rate.

#### Imputation may benefit robustness or interpretability

A good imputation may bring the benefit of recovering a meaningful missing value, reflecting a biological or clinical reality rather than operational constraints. For instance, the weight of a patient may be measured upon scheduled admission to a hospital but not at the emergency department. A predictive model based on an imputed underlying value may lend itself better to mechanistic interpretation than a model implicitly capturing missingness such as MIA. In addition, using missingness to drive prediction may be more fragile, e.g., to changes in the operational process. In such a case, shifts in the missing data patterns should be closely monitored [6,41,42] because they could seriously alter prediction performance. Indeed, machine learning models building their predictions on “shortcuts” in the data—not directly related to outcome of interest but rather to the acquisition—sometimes generalize less well to new hospitals [43]. Nevertheless, in health care the mere presence of a measure, such as a colonoscopy, is often an indication in itself.

### Limitations and further work

#### Limitations: not all differences are significant

Relative performance of approaches varies across datasets, which is not surprising because no prediction model is expected to dominate on all data. The diversity of the datasets and the statistical analysis grounds the generality of the findings. Yet, not all differences are significant at large sample sizes. This lack of significance can simply be explained because of a small statistical power of the benchmark because only a few datasets are available to test these very large sample size settings (only 4 tasks at the *n* = 100,000 size).

More datasets would probably have made more differences significant. Yet, the benchmarks presented here already incurred large computational costs, owing to the nested cross-validation: ∼520,000 CPU hours. Also, the findings build upon 13 different tasks, markedly more than the typical machine learning benchmark: only 6% of empirical results published at NeurIPS and 8% ICLR (both leading machine learning venues) build upon >10 datasets [44].

#### Limitations: imputation quality is not assessed

All the conclusions of this study pertain to prediction and do not allow us to conclude regarding imputation’s ability to accurately reconstruct missing values. The focus of our study is indeed on prediction.

#### Further work: more benchmarks would be interesting, and costly

To limit computation costs and mimic typical usage, no hyperparameter tuning was performed on the parameters of the imputers. Recently, software tools have been introduced to perform model selection on imputation jointly with the supervised step [12,45]. Further evaluation could quantify the gains brought by such joint model selection, although it would need sizable computational resources.

Further work could test more supervised learning models. The motivation of the present study was not to find the absolute best pipeline but rather to understand compromises that hold across datasets and are readily usable.

### Conclusion

Extensive benchmarking on health databases reveals trends in the performance of methods to build predictive models handling missing values. First, directly incorporating missing values in tree-based models with MIA gives a small but systematic improvement in prediction performance over prior imputation. Second, the computational cost of imputation using MICE or KNN becomes intractable for large datasets. Third, gradient-boosted trees give better predictions than linear models. Fourth, bagging increases predictive performance but with a severe computational cost. Fifth, good imputation does not imply good prediction because both have different trade-offs. Finally, the experiments reveal that the missingness is informative. Overall, a novel message of this benchmark is that for building predictive models, supervised learning directly handling missing values should be considered, beyond imputation.

## Potential Implications

This work suggests a departure from current practices: supervised learning directly handling missing values can be preferable to imputation. In particular, classic conditional expectation methods can be computationally intractable in terms of both time and memory on large datasets. Constant imputation with the mask also performs well with little cost.

## Detailed Benchmarking Methodology

### Experiment

We selected 4 real databases with missing values described in section Health databases. From them we empirically defined 13 prediction tasks, i.e., a set of input features and an outcome to predict, with the intent of covering as diverse a range of use cases as possible: regressions, classifications, diverse outcomes, and diverse feature types (numerical, ordinal, and categorical). We subsampled the datasets to study 4 sizes: 2,500, 10,000, 25,000 and 100,000 samples. We selected a subset of features from the databases for each prediction task using 2 approaches. We manually selected or defined features based on articles or automatically selected 100 encoded features using an univariate ANOVA selection. We often used the latter because it has the advantage of not requiring expert knowledge to define the features. Manual selection keeps fewer features than our automated selection. Note that we one-hot encoded categorical features before selecting 100 encoded features with ANOVA. Fewer than 100 non-encoded features may thus be involved in the task. The ANOVA is fitted on one-third of the samples, and the remaining two-thirds are kept for fitting and evaluating the methods. To reduce bias induced by the choice of subset on which the ANOVA is fit, we ran 5 trials in which the subset is redrawn each time and the scores and times are averaged. Task having their features manually selected are given the entire sample and only 1 trial is performed. Each of the 12 methods is given the exact same features and cross-validation folds.

The next step consists in benchmarking the 12 methods of Table 1 on the defined prediction tasks. We used the implementation from scikit-learn [20] for all methods (see Table A4). Two nested cross-validations are used. The outer one yields 5 training and test sets. On each training set, we perform a cross-validated hyperparameter search (the inner cross-validation) and select the best hyperparameters. We evaluate the best model on the respective test set. We assess the quality of the prediction with a coefficient of determination for regressions and the area under the receiver operating curve for classification. We average the scores obtained on the 5 test sets of the outer cross-validation to give the final score. Finally, we compare averaged prediction scores each to the other.

We also monitored training and imputation times to add time concerns to our analysis. A detailed description of the experimental method is available on protocols.io [46]. A link to the code of the experiments is given in Availability of Source Code and Requirements.

### Plotting method

#### Figures on prediction scores

The experiment gives 1 prediction score per fold, per trial, per task, per method, per size. Cross-validations aggregate and average scores across the folds and trials, resulting in a mean score for each of the (task, method, size) combinations. For each (task, size) pair, we computed a reference score by averaging the scores obtained by the 12 methods on the corresponding task and size. The plotted metric is what we call the relative prediction score (i.e., the deviation of the prediction score from the reference score) for each permutation of (task, method, size). We created 1 box plot for each of the 4 sizes with the same structure: the relative prediction score on the x-axis and the 12 methods on the y-axis. Each is overlaid with a scatter plot plotting the relative prediction score per (task, method, size). The scatter plot shares its x-axis with the box plot. On the y-axis, however, each dot is given a y-coordinate according to its method and database so that scores coming from a same method and database are plotted on the same horizontal line.

#### Figures on computation time

Computational time plots follow the same structure. The metric of interest is now the total training time. It includes imputation time and the full hyperparameter tuning time. It is evaluated using the computer’s process time instead of wall-clock time. The total training time of MIA is taken as reference time for each (task, size). The relative total training time is computed by dividing by the reference time. The x-scale is logarithmic to better apprehend comparison on large scales.

## Availability of Source Code and Requirements

- Project name: Benchmarking missing-values approaches for predictive models on health databases
- Project home page: https://github.com/aperezlebel/benchmark_mv_approaches
- Operating system: Platform independent
- Programming language: Python 3.7.6
- Other requirements: all requirements are listed in the requirements.txt file of the repository.
- License: MIT

## Data Availability

All supporting data and materials are available in the GigaScience GigaDB database [47]. The datasets supporting the results of this article are available at the following URL.

- Traumabase: by contacting the team at http://www.traumabase.eu/en_US/contact.
- UKBB: upon application at https://www.ukbiobank.ac.uk/register-apply/.
- MIMIC-III: upon application at https://mimic.physionet.org/gettingstarted/access/.
- NHIS: freely available at https://www.cdc.gov/nchs/nhis/nhis_2017_data_release.htm.

A thorough description of the protocols of the experiments conducted in this article is available on protocols.io [46].

## Abbreviations

ANOVA: analysis of variance; AUC: area under the curve; CPU: central processing unit; ICU: intensive care unit; KNN: *k*-nearest neighbors; MAR: missing at random; MIA: missing incorporated in attribute; MNAR: missing not at random; MIMIC: Medical Information Mart for Intensive Care; NHIS: National Health Interview Survey; LOWESS: locally weighted scatterplot smoothing; UKBBUK: UK Biobank.

## Ethical Approval

Only secondary data use was involved in this work. Each of the databases has previously been approved by the Ethical Review Board. Access to each database was obtained according to the corresponding rules and authorization, when applicable (MIMIC, Traumabase, UKBB).

## Competing Interests

The authors declare that they have no competing interests.

## Funding

G.V., J.J., and M.L. acknowledge funding from DataAI under the MissingBigData grant. G.V. and M.L. acknowledge funding from Agence National de la Recherche under the DirtyData grant (ANR-17-CE23-0018). J.J. acknowledges funding from Agence National de la Recherche under the MUSE grant (ANR-16-IDEX-0006). A.P.L. was partially funded by a Mitacs Globalink Research Award for research in Canada. J.B.P. is partially funded by the National Institutes of Health (NIH) NIH-NIBIB P41 EB019936 (ReproNim) NIH-NIMH R01 MH083320 (CANDIShare) and NIH RF1 MH120021 (NIDM), the National Institute of Mental Health of the NIH under Award No. R01MH096906 (Neurosynth), as well as the Canada First Research Excellence Fund, awarded to McGill University for the Healthy Brains for Healthy Lives initiative and the Brain Canada Foundation with support from Health Canada.

## Authors’ Contributions

Following the CRediT Contributor Roles Taxonomy:

- A.P.L.: Investigation, Data curation, Formal Analysis, Writing - original draft, Writing - review & editing.
- G.V.: Funding acquisition, Conceptualization, Supervision, Writing - review & editing, Validation.
- M.L.: Supervision, Writing - review & editing, Validation.
- J.J.: Writing - review & editing.
- J.B.P.: Funding acquisition, Supervision, Writing - review & editing, Validation.

## Authors’ Information

A.P.L. is a Ph.D. student in machine learning at Inria. This work started as an internship at the Montreal Neurological Institute of McGill University and extended into the early months of the Ph.D. program.

## Supplementary Material

giac013_GIGA-D-21-00180_Original_Submission

giac013_GIGA-D-21-00180_Revision_1

giac013_GIGA-D-21-00180_Revision_2

giac013_Response_to_Reviewer_Comments_Revision_1

giac013_Response_to_Reviewer_Comments_Revision_2

giac013_Reviewer_1_Report_Original_Submission Jamie Perin -- 8/9/2021 Reviewed

giac013_Reviewer_1_Report_Revision_1 Jamie Perin -- 12/15/2021 Reviewed

giac013_Reviewer_2_Report_Original_Submission Jason Poulos -- 8/16/2021 Reviewed

giac013_Reviewer_2_Report_Revision_1 Jason Poulos -- 12/13/2021 Reviewed

## Figures and Tables

**Figure A4.**
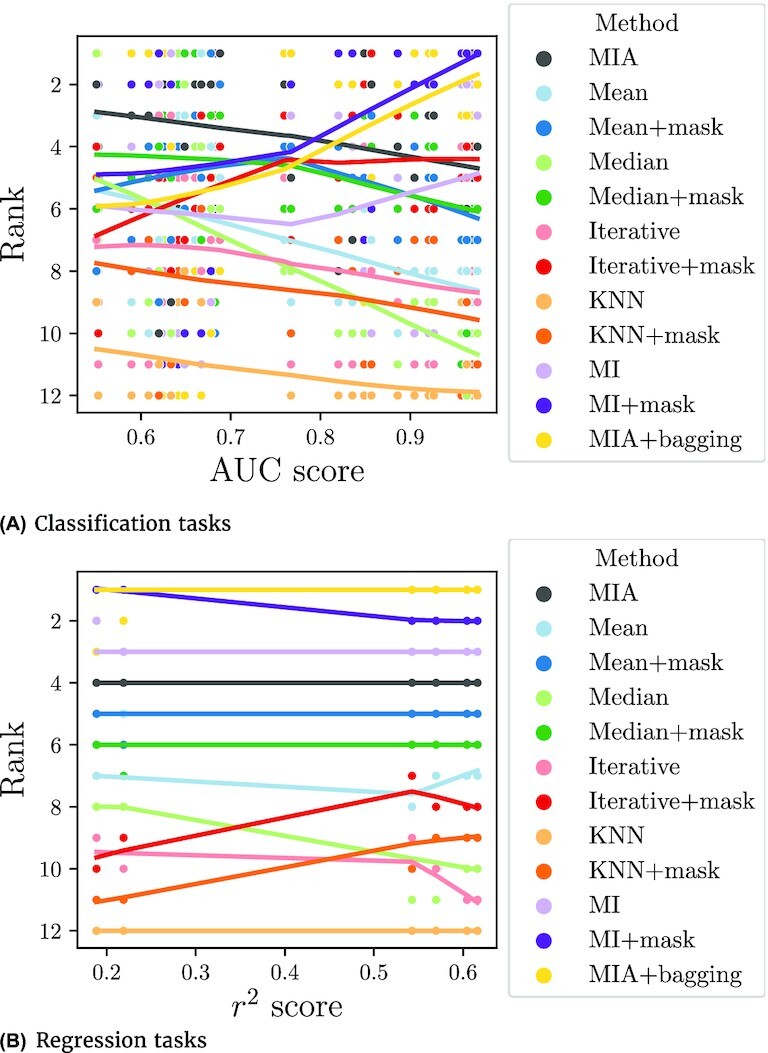
**Effect of difficulty on the ranks of the methods**. For each task and size, the average score obtained by the methods is taken as a proxy of its difficulty. Local regressions (LOWESS) are plotted for each method to better visualize trends.

**Figure A6.**
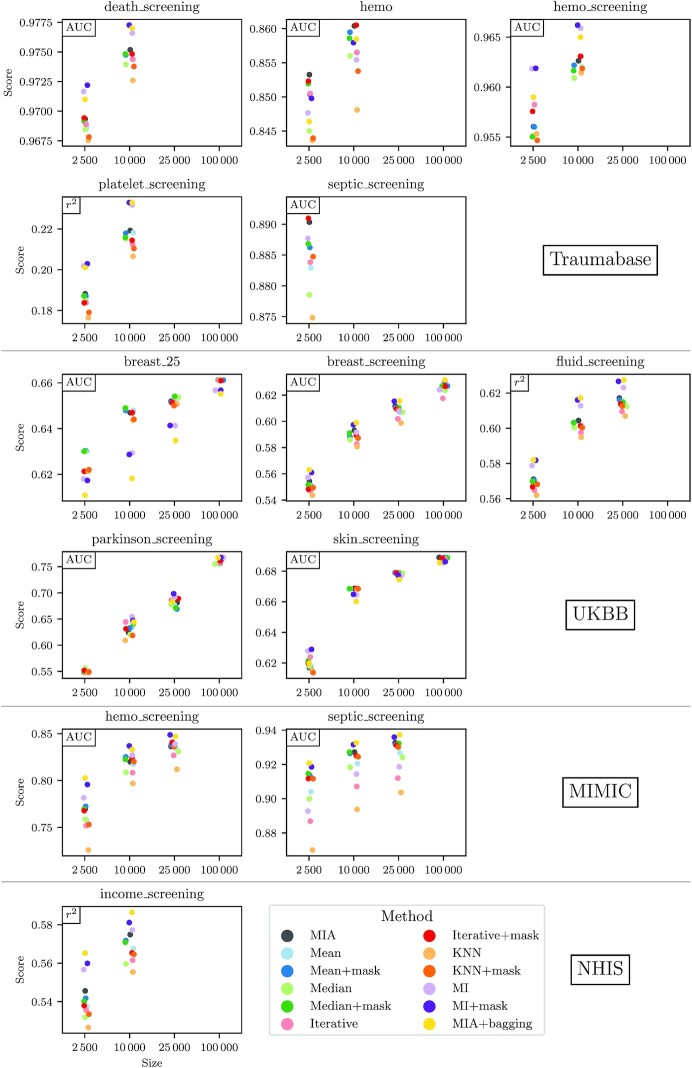
**Scores of the tree-based methods as a function of the training size**. Detailed scores of Figure 1 broken out by task.

## Appendix

### Supplementary experiment: linear models or trees?

#### Protocol

This supplementary experiment uses the same pipeline as the main experiment except that imputation is paired with linear models instead of boosted trees as summarized in Table A1. We used ridge for regressions, and $\ell_2$-penalized logistic regression for classifications.

**Table A1:** Methods compared in the supplementary experiment

In-article name	Imputer	Mask	Predictive model
Boosted trees+MIA	–	–	Boosted trees
Linear+Mean	Mean	No	Ridge/Logit
Linear+Mean+mask	Mean	Yes	Ridge/Logit
Linear+Med	Median	No	Ridge/Logit
Linear+Med+mask	Median	Yes	Ridge/Logit
Linear+Iter	Iterative	No	Ridge/Logit
Linear+Iter+mask	Iterative	Yes	Ridge/Logit
Linear+KNN	KNN	No	Ridge/Logit
Linear+KNN+mask	KNN	Yes	Ridge/Logit

#### Findings: trees with MIA improve upon linear models

MIA with boosted trees outperforms all 8 combinations of imputers with linear models, on every size and every database. Fig.A1A shows that MIA obtained the best average rank of 1.3 far ahead of other methods. The following ones are Linear+mean+mask, Linear+med+mask and Linear+iter+mask with ranks of 3.9, 4.0 and 4.5 respectively. The 1-sided Wilcoxon signed-rank test confirms this claim. Table A3 shows that MIA with boosted trees is significantly better than every linear methods on the first 2 sizes even at the Bonferroni-corrected level. The null hypothesis of the Friedman test is rejected below the 0.05 level except for the last size as shown on Table A2B. Thus methods are not equivalent for the first three sizes. The Nemenyi test on Fig. A2B confirms that results are not significant for the larger size.

**Table A2:** **Friedman test, correction by Iman and Davenport and Nemenyi test**. CD is the critical distance and N the number of tasks for each size

(a) Tree-based methods of Table 1.						
	$\chi ^2_F$	$\chi ^2_F$ p-value	*F_F_*	*F_F_* p-value	CD	N
Size						
2500	73	9.6e-11	12	7.4e-16	4.6	13
10000	76	2.3e-11	15	6.1e-18	4.8	12
25000	30	2.5e-03	3.9	2.4e-04	6.3	7
100000	10	0.43	1.2	0.35	6.8	4
**(b) Boosted-trees and linear methods of Table A1**.						
2500	41	5.1e-06	7.8	5.2e-08	3.3	13
10000	50	9.7e-08	12	1.7e-11	3.5	12
25000	23	5.6e-03	4.3	6.2e-04	4.5	7
100000	-19	1	-1.1	1	6	4

Moreover, we were expecting the mean and median imputations to give bad results being paired with linear models as shown in [48]. Not only these results confirm our expectations, but they also show that non-constant imputation models give similar results when paired with a linear model. As before, masked versions perform slightly better than their no-mask counterpart.

However, gradient-boosted trees with MIA are a lot slower than imputation with linear models. Fig. A1B shows that boosted trees with MIA is up to 500 times slower than constant imputations with linear models. Also, conditional imputation leads to slower computations than mean and median imputation. Given the low gain obtained against mean and median imputation, they are of limited interest.

The main takeaway is the outperformance in score of MIA with gradient-boosted trees over imputation with linear models when it comes to handling missing values. This outperformance comes with a cost: a much longer computation time.

### Significance tests

In the following paragraphs, we took the notations and formulations of Demšar [38]. We consider *k* algorithms and *N* datasets. We note $r_i^j$ the rank of the *j*-th algorithm on the *i*-th dataset. Note $R_j = \frac{1}{N}\sum_i r_i^j$ the average rank.

#### Friedman test

The Friedman statistic $\chi_F^2$ is distributed according to a chi-square distribution with $k-1$ degrees of freedom.

(1) \begin{eqnarray*}
\newcommand{\bra}[1]{\left[ #1 \right]}
\chi_F^2 = \frac{12N}{k(k+1)}\bra{\sum_jR_j^2 - \frac{k(k+1)^2}{4}}
\end{eqnarray*}

Iman and Davenport [49] derived a less conservative statistic $F_F$ which is distributed according to the F-distribution with $k-1$ and $(k-1)(N-1)$ degrees of freedom.

(2) \begin{eqnarray*}
\newcommand{\bra}[1]{\left[ #1 \right]}
F_F = \frac{(N-1)\chi^2_F}{N(k-1)-\chi^2_F}
\end{eqnarray*}

Both statistics (1) and (2) are given on Table A2 with their associated P-values for the 2 sets of methods and the 4 sizes of dataset.

#### Nemenyi test

Once the Friedman test is rejected, the Nemenyi test can be applied. It provides a critical difference $CD$ which is the minimal difference between the average ranks of two algorithms for them to be significantly different.

(3) \begin{eqnarray*}
CD = q_{\alpha}\sqrt{\frac{k(k+1)}{6N}}
\end{eqnarray*}

Values of $q_{\alpha}$ are given in Table 5 of Demšar [38]. Values of critical differences for the 2 sets of methods and the 4 sizes of dataset are given in Table A2.

#### Wilcoxon signed-rank test

To compute the 1-sided Wilcoxon signed-rank test, we used the wilcoxon function of the scipy.stats module between the 13 average scores of MIA against the ones of every other methods. Resulting P-values are given in Table A3 for the 4 sizes of dataset.

**Table A3:** **One-sided Wilcoxon signed-rank test**. p-values of the one-sided right test on the difference of score between MIA and every other method for Table A3a, and between gradient-boosted trees and linear models for Table A3b. p-values below the 0.05 level are marked with ^⋆^. p-values below the Bonferroni corrected level are marked with ^⋆⋆^. When the reversed test (i.e. one-sided left) is significant instead, p-values are marked with ^⋆( > )^ and ^⋆⋆( > )^ following the same rule

**(a)MIA vs imputation**. Bonferroni level: 0.05/19 = 2.6 × 10^−3^. Rejecting the null hypothesis means MIA performed better than the compared method				
Size	2500	10000	25000	100000
Method				
Mean	1.2e-03^⋆⋆^	4.6e-02^⋆^	2.3e-02^⋆^	6.2e-02
Mean+mask	4.0e-02^⋆^	2.3e-01	1.5e-01	6.2e-02
Median	5.2e-03^⋆^	1.7e-03^⋆⋆^	2.3e-02^⋆^	6.2e-02
Median+mask	4.0e-02^⋆^	2.1e-01	1.5e-01	1.2e-01
Iterative	5.2e-03^⋆^	3.2e-02^⋆^	3.9e-02^⋆^	6.2e-02
Iterative+mask	2.4e-02^⋆^	2.1e-01	4.7e-01	6.2e-02
KNN	1.2e-04^⋆⋆^	2.4e-04^⋆⋆^	3.1e-02^⋆^	
KNN+mask	1.2e-04^⋆⋆^	7.3e-04^⋆⋆^	3.1e-02^⋆^	
MI	8.1e-01	6.6e-01	3.4e-01	1.2e-01
MI+mask	9.9e-01^⋆( > )^	9.6e-01^⋆( > )^	9.2e-01	4.4e-01
MIA+bagging	9.7e-01^⋆( > )^	9.4e-01	7.7e-01	3.1e-01
Linear+Mean	6.1e-04^⋆⋆^	4.9e-04^⋆⋆^	7.8e-03^⋆^	6.2e-02
Linear+Mean+mask	8.5e-04^⋆⋆^	7.3e-04^⋆⋆^	1.6e-02^⋆^	6.2e-02
Linear+Med	6.1e-04^⋆⋆^	4.9e-04^⋆⋆^	7.8e-03^⋆^	6.2e-02
Linear+Med+mask	6.1e-04^⋆⋆^	4.9e-04^⋆⋆^	1.6e-02^⋆^	6.2e-02
Linear+Iter	3.1e-03^⋆^	1.2e-03^⋆⋆^	1.6e-02^⋆^	6.2e-02
Linear+Iter+mask	2.3e-03^⋆⋆^	1.2e-03^⋆⋆^	1.6e-02^⋆^	6.2e-02
Linear+KNN	1.2e-04^⋆⋆^	2.4e-04^⋆⋆^	1.6e-02^⋆^	5.0e-01
Linear+KNN+mask	1.2e-04^⋆⋆^	2.4e-04^⋆⋆^	3.1e-02^⋆^	5.0e-01
**(b) Gradient-boosted trees vs linear models**. Bonferroni level: 0.05/8 = 6.25 × 10^−3^. Rejecting the null hypothesis means gradient-boosted trees performed better than linear models for the given imputer				
Size	2500	10000	25000	100000
Imputer				
Mean	1.2e-03^⋆⋆^	4.9e-04^⋆⋆^	1.6e-02^⋆^	6.2e-02
Mean+mask	1.7e-03^⋆⋆^	4.9e-04^⋆⋆^	1.6e-02^⋆^	6.2e-02
Median	6.1e-04^⋆⋆^	7.3e-04^⋆⋆^	1.6e-02^⋆^	6.2e-02
Median+mask	8.5e-04^⋆⋆^	2.4e-04^⋆⋆^	1.6e-02^⋆^	6.2e-02
Iterative	4.0e-03^⋆⋆^	1.2e-03^⋆⋆^	2.3e-02^⋆^	6.2e-02
Iterative+mask	2.3e-03^⋆⋆^	1.2e-03^⋆⋆^	1.6e-02^⋆^	6.2e-02
KNN	8.5e-04^⋆⋆^	7.3e-04^⋆⋆^	3.1e-02^⋆^	
KNN+mask	8.5e-04^⋆⋆^	4.9e-04^⋆⋆^	3.1e-02^⋆^	

**Table A4:** Scikit-learn’s implementations of the methods

In-article name	Scikit-learn’s method
Boosted trees	HistGradientBoostingRegressor,
	HistGradientBoostingClassifier
Linear model	Ridge, LogisticRegression
Mean, Mean+mask	SimpleImputer
Median, Median+mask	SimpleImputer
Iterative, Iterative+mask	IterativeImputer
KNN, KNN+mask	KNNImputer
ANOVA selection	f_regression, f_classif
Permutation importance	permutation_importance
Bagging	BaggingRegressor, BaggingClassifier

**Table A5:** **Correlation between features**. Average number of ordinal and numerical features correlated to other ordinal or numerical features with an absolute correlation coefficient larger than thresholds {0.1, 0.2, 0.3}, averaged on all ordinal and numerical features of the task and expressed in percentage of the number of ordinal and numerical features in the task. For example in the task “death_screening”, a numerical or ordinal feature has an absolute correlation value greater than 0.01 with 68% of the ordinal and numerical features of the task in average

			Threshold		
			0.1	0.2	0.3
Database	Task	# features			
Traumabase	death_screening	92	68%	41%	22%
	hemo	12	50%	23%	12%
	hemo_screening	76	65%	36%	20%
	platelet_screening	90	67%	40%	22%
	septic_screening	76	68%	37%	18%
UKBB	breast_25	11	40%	20%	19%
	breast_screening	100	26%	12%	8%
	fluid_screening	100	21%	10%	6%
	parkinson_screening	100	28%	16%	11%
	skin_screening	100	24%	11%	8%
MIMIC	hemo_screening	100	22%	6%	3%
	septic_screening	100	21%	6%	2%
NHIS	income_screening	78	15%	6%	4%
Average		79	40%	20%	12%

**Table A6:** **Overview of the prediction tasks used in this article**. For selection, ‘A’ means ANOVA and ‘M’ means manual. Type ‘C’ is classification and type ‘R’ is regression. The number of features is given after encoding and selection. Since this number may vary between trials, we average it on the 5 trials for the ANOVA selection. Target is the name of the feature to predict in the original database or a formula to build a new feature to predict from the existing ones

		Score	Scorer	Selection	Number of samples	Number of features	Type	Target	Description
Database	Task								
Traumabase	death_screening	0.96	AUC	A	12341	98	C	Décès == Oui	Predict the death of patients.
	hemo	0.85	AUC	M	19569	12	C	Choc hémorragique (? 4 CGR sur 6h) == Oui	Predict the hemorrhagic shock using features defined in [29].
	hemo_screening	0.95	AUC	A	13047	89	C	Choc hémorragique (? 4 CGR sur 6h) == Oui	Predict the hemorrhagic shock using ANOVA selection.
	platelet_screening	0.17	R2	A	12696	96	R	Plaquettes	Predict the level of platelet on arrival at the hospital using ANOVA selection.
	septic_screening	0.86	AUC	A	6046	90	C	Choc septique == Oui	Predict septic shock.
UKBB	breast_25	0.60	AUC	M	273384	66	C	One of: C500, C501, C502, C503, C504, C505, C506, C508, C509; found in one of: 41270-0.0, 41202-0.0, 41204-0.0, 40006-0.0; or one of: 1740, 1743, 1744, 1745, 1748, 1749; found in one of: 41271-0.0, 41203-0.0, 41205-0.0, 40013-0.0	Predict malignant neoplasm of breast on female patients only using features defined in [31].
	breast_screening	0.59	AUC	A	182257	100	C	Same as breast_25	Predict malignant neoplasm of breast on female patients only using ANOVA selection.
	fluid_screening	0.56	R2	A	110308	100	R	20016-0.0	Predict the fluid intelligence score.
	parkinson_screening	0.65	AUC	A	335005	100	C	One of: G20, G210, G211, G212, G213, G214, G218, G219, G22, F023; found in one of: 41270-0.0, 41202-0.0, 41204-0.0, 40006-0.0; or one of: 3320, 3321; found in one of: 41271-0.0, 41203-0.0, 41205-0.0, 40013-0.0	Predict Parkinson’s disease.
	skin_screening	0.64	AUC	A	335005	100	C	One of: C430, C431, C432, C433, C434, C435, C436, C437, C438, C439, C440, C441, C442, C443, C444, C445, C446, C447, C448, C449; found in one of: 41270-0.0, 41202-0.0, 41204-0.0, 40006-0.0; or one of: 1720, 1723, 1725, 1726, 1727, 1729, 1730, 1731, 1732, 1733, 1734, 1735, 1736, 1737, 1739; found in one of: 41271-0.0, 41203-0.0, 41205-0.0, 40013-0.0	Predict melanoma and other malignant neoplasms of skin.
MIMIC	hemo_screening	0.74	AUC	A	30836	100	C	One of: 78559, 99809, 9584; found in ICD9_CODE	Predict the hemorrhagic shock from the LABEVENTS table only.
	septic_screening	0.87	AUC	A	30836	100	C	ICD9_CODE == 78552	Predict the septic shock from the LABEVENTS table only.
NHIS	income_screening	0.52	R2	A	20987	96	R	ERNYR-P	Predict the income earned on the previous year with information from tables: household, family, person and adult.

**Table A7:** Scoresand ranks of the tree based methods described in Table 1

**(a) Scores relative to the absolute reference score plotted in Figure 1a**. Values in bold are the reference scores and are absolute. Other scores are given relative to the reference score of their task and size.														
	Database	Traumabase					UKBB					MIMIC		NHIS
	Task	death_screening	hemo	hemo_screening	platelet_screening	septic_screening	breast_25	breast_screening	fluid_screening	parkinson_screening	skin_screening	hemo_screening	septic_screening	income_screening
Size	Method													
2500	MIA	-8e-5	+5e-3	-1e-3	-4e-4	+4e-3	+7e-3	+2e-3	+5e-5	+2e-3	-3e-3	+3e-3	+8e-3	+3e-3
	Mean	-8e-4	+1e-3	-1e-3	-2e-3	-3e-3	+6e-3	-1e-3	-2e-3	+2e-3	-2e-3	-1e-2	-6e-4	-5e-3
	Mean+mask	-5e-4	+2e-3	-1e-3	-1e-3	+2e-4	+7e-3	-8e-4	-5e-4	-2e-3	-3e-3	+5e-3	+9e-3	-1e-3
	Median	-1e-3	-4e-3	-2e-3	-3e-3	-8e-3	+6e-3	-1e-3	-4e-3	+6e-3	-2e-3	-8e-3	-5e-3	-1e-2
	Median+mask	-2e-4	+3e-3	-2e-3	-1e-3	+7e-4	+7e-3	-9e-4	-1e-3	-2e-3	+1e-3	+2e-3	+1e-2	-3e-3
	Iterative	-5e-4	+2e-3	+1e-3	-5e-3	-2e-3	-2e-3	-5e-3	-6e-3	-2e-3	+4e-3	-2e-2	-2e-2	-7e-3
	Iterative+mask	+4e-6	+4e-3	+3e-4	-5e-3	+5e-3	-2e-3	-4e-3	-4e-3	+1e-3	-4e-4	+6e-4	+7e-3	-5e-3
	KNN	-2e-3	-5e-3	-2e-3	-1e-2	-1e-2	-2e-3	-9e-3	-9e-3	-3e-3	-5e-3	-4e-2	-3e-2	-2e-2
	KNN+mask	-2e-3	-5e-3	-3e-3	-1e-2	-1e-3	-2e-3	-3e-3	-3e-3	-1e-3	-6e-3	-1e-2	+7e-3	-9e-3
	Iterative+Bagging	+2e-3	-1e-3	+5e-3	+1e-2	+2e-3	-6e-3	+5e-3	+8e-3		+8e-3	+1e-2	-1e-2	+1e-2
	Iterative+mask+Bagging	+3e-3	+1e-3	+5e-3	+1e-2	+7e-3	-6e-3	+9e-3	+1e-2		+9e-3	+3e-2	+1e-2	+2e-2
	MIA+Bagging	+2e-3	-2e-3	+2e-3	+1e-2	+6e-3	-1e-2	+1e-2	+1e-2		-4e-4	+4e-2	+2e-2	+2e-2
	Reference score	**0.97**	**0.85**	**0.96**	**0.19**	**0.89**	**0.62**	**0.55**	**0.57**	**0.55**	**0.62**	**0.77**	**0.90**	**0.54**
10000	MIA	+2e-4	+4e-3	-4e-4	+3e-4		+5e-3	+4e-3	-1e-5	-9e-3	+1e-3	-1e-4	+6e-3	+5e-3
	Mean	-6e-4	-3e-4	-1e-3	-8e-4		+6e-3	-3e-3	-3e-3	+3e-4	+2e-3	-3e-3	-2e-4	-2e-3
	Mean+mask	-2e-4	+3e-3	-8e-4	-1e-3		+6e-3	-1e-3	-1e-3	-6e-4	+1e-3	+5e-3	+6e-3	+2e-3
	Median	-1e-3	-8e-4	-2e-3	-4e-3		+8e-3	-3e-3	-4e-3	-1e-2	+1e-3	-1e-2	-2e-3	-1e-2
	Median+mask	-1e-4	+2e-3	-1e-3	-3e-3		+7e-3	+2e-3	-1e-3	+8e-3	+1e-3	+3e-3	+6e-3	+1e-3
	Iterative	-6e-4	-3e-4	+8e-5	-6e-3		+6e-3	-7e-3	-7e-3	+1e-2	+1e-3	-1e-2	-1e-2	-8e-3
	Iterative+mask	-1e-4	+4e-3	+9e-5	-5e-3		+5e-3	-1e-4	-3e-3	-2e-3	+1e-3	+4e-3	+4e-3	-4e-3
	KNN	-2e-3	-9e-3	-2e-3	-1e-2		+2e-3	-9e-3	-9e-3	-2e-2	+1e-3	-2e-2	-3e-2	-1e-2
	KNN+mask	-1e-3	-3e-3	-1e-3	-9e-3		+2e-3	-2e-3	-4e-3	-1e-2	+1e-3	+1e-4	+4e-3	-5e-3
	Iterative+Bagging	+2e-3	-1e-3	+3e-3	+1e-2		-1e-2	+2e-3	+8e-3	+2e-2	-3e-3	+7e-3	-6e-3	+7e-3
	Iterative+mask+Bagging	+2e-3	+1e-3	+3e-3	+1e-2		-1e-2	+8e-3	+1e-2	+1e-2	-2e-3	+2e-2	+1e-2	+1e-2
	MIA+Bagging	+2e-3	+2e-3	+2e-3	+1e-2		-2e-2	+1e-2	+1e-2	+1e-2	-7e-3	+1e-2	+1e-2	+2e-2
	Reference score	**0.97**	**0.86**	**0.96**	**0.22**		**0.64**	**0.59**	**0.60**	**0.63**	**0.67**	**0.82**	**0.92**	**0.57**
25000	MIA						+3e-3	+3e-3	+9e-4	-1e-3	+9e-4	+8e-4	+6e-3	
	Mean						+4e-3	-2e-3	-2e-3	-5e-3	+9e-4	-1e-3	+5e-4	
	Mean+mask						+3e-3	+2e-3	-1e-4	-1e-2	+1e-3	+2e-3	+5e-3	
	Median						+5e-3	-2e-3	-4e-3	-5e-3	+5e-4	-5e-3	-2e-3	
	Median+mask						+5e-3	+2e-3	-1e-3	-1e-2	+9e-4	+1e-3	+6e-3	
	Iterative						+3e-3	-7e-3	-7e-3	+4e-3	+5e-4	-9e-3	-1e-2	
	Iterative+mask						+3e-3	+8e-4	-2e-3	+6e-3	+8e-4	+5e-3	+5e-3	
	KNN						+2e-3	-1e-2	-9e-3			-2e-2	-2e-2	
	KNN+mask						+1e-3	-1e-4	-4e-3			+6e-4	+4e-3	
	Iterative+Bagging						-7e-3	-8e-4	+7e-3	+1e-2	-1e-3	+3e-3	-8e-3	
	Iterative+mask+Bagging						-7e-3	+7e-3	+1e-2	+2e-2	-7e-4	+1e-2	+1e-2	
	MIA+Bagging						-1e-2	+7e-3	+1e-2	+4e-4	-4e-3	+1e-2	+1e-2	
	Reference score						**0.65**	**0.61**	**0.62**	**0.68**	**0.68**	**0.84**	**0.93**	
100000	MIA						+2e-3	+2e-3		+3e-3	+1e-3			
	Mean						+1e-3	-2e-3		-5e-3	+7e-4			
	Mean+mask						+2e-3	+9e-4		-2e-3	+1e-3			
	Median						+2e-3	-2e-3		-6e-3	+5e-4			
	Median+mask						+2e-3	+9e-4		-5e-3	+1e-3			
	Iterative						+1e-3	-9e-3		-4e-3	+2e-4			
	Iterative+mask						+1e-3	+6e-4		+1e-4	+8e-4			
	KNN													
	KNN+mask													
	Iterative+Bagging						-3e-3	-2e-3		+6e-3	-2e-3			
	Iterative+mask+Bagging						-3e-3	+5e-3		+6e-3	-2e-3			
	MIA+Bagging						-4e-3	+5e-3		+6e-3	-2e-3			
	Reference score						**0.66**	**0.63**		**0.76**	**0.69**			
Average	Reference score	**0.97**	**0.85**	**0.96**	**0.20**	**0.89**	**0.64**	**0.59**	**0.60**	**0.66**	**0.66**	**0.81**	**0.92**	**0.56**
														

**(b) Ranks computed from the relative scores in Table A7a**. Best average ranks are in bold.																			
	Database	Traumabase					UKBB					MIMIC		NHIS	Average				
	Task	death_screening	hemo	hemo_screening	platelet_screening	septic_screening	breast_25	breast_screening	fluid_screening	parkinson_screening	skin_screening	hemo_screening	septic_screening	income_screening	Traumabase	UKBB	MIMIC	NHIS	All
Size	Method																		
2500	MIA	5	1	6	4	4	1	4	4	2	10	5	5	4	4.0	4.2	5.0	4.0	4.3
	Mean	9	6	8	7	10	5	8	7	3	7	9	8	8	8.0	6.0	8.5	8.0	7.6
	Mean+mask	7	5	7	5	7	2	5	5	8	9	4	4	5	6.2	5.8	4.0	5.0	5.2
	Median	10	10	10	8	11	4	7	9	1	8	8	9	11	9.8	5.8	8.5	11.0	8.8
	Median+mask	6	3	11	6	6	3	6	6	6	4	6	3	6	6.4	5.0	4.5	6.0	5.5
	Iterative	8	4	4	9	9	9	11	11	7	3	11	11	9	6.8	8.2	11.0	9.0	8.8
	Iterative+mask	4	2	5	10	3	8	10	10	4	5	7	6	7	4.8	7.4	6.5	7.0	6.4
	KNN	12	12	9	12	12	7	12	12	9	11	12	12	12	11.4	10.2	12.0	12.0	11.4
	KNN+mask	11	11	12	11	8	6	9	8	5	12	10	7	10	10.6	8.0	8.5	10.0	9.3
	Iterative+Bagging	2	8	2	2	5	10	3	3		2	3	10	3	3.8	4.5	6.5	3.0	4.4
	Iterative+mask+Bagging	1	7	1	1	1	11	2	2		1	2	2	2	**2.2**	**4.0**	2.0	2.0	**2.6**
	MIA+Bagging	3	9	3	3	2	12	1	1		6	1	1	1	4.0	5.0	**1.0**	**1.0**	2.8
10000	MIA	4	2	6	4		6	3	4	9	2	8	3	4	4.0	**4.8**	5.5	4.0	4.6
	Mean	8	7	8	5		3	9	7	6	1	9	8	7	7.0	5.2	8.5	7.0	6.9
	Mean+mask	7	3	7	6		4	7	5	7	5	4	5	5	5.8	5.6	4.5	5.0	5.2
	Median	10	9	12	8		1	10	10	10	4	10	9	11	9.8	7.0	9.5	11.0	9.3
	Median+mask	6	4	10	7		2	5	6	5	7	6	4	6	6.8	5.0	5.0	6.0	5.7
	Iterative	9	8	5	10		5	11	11	3	8	11	11	10	8.0	7.6	11.0	10.0	9.2
	Iterative+mask	5	1	4	9		7	6	8	8	3	5	6	8	4.8	6.4	5.5	8.0	6.2
	KNN	12	12	11	12		9	12	12	12	9	12	12	12	11.8	10.8	12.0	12.0	11.6
	KNN+mask	11	11	9	11		8	8	9	11	6	7	7	9	10.5	8.4	7.0	9.0	8.7
	Iterative+Bagging	3	10	2	3		10	4	3	1	11	3	10	3	4.5	5.8	6.5	3.0	5.0
	Iterative+mask+Bagging	1	6	1	1		11	2	2	2	10	1	2	2	**2.2**	5.4	**1.5**	2.0	**2.8**
	MIA+Bagging	2	5	3	2		12	1	1	4	12	2	1	1	3.0	6.0	1.5	**1.0**	2.9
25000	MIA						4	3	4	6	2	7	3			**3.8**	5.0		4.4
	Mean						3	10	7	8	4	9	8			6.4	8.5		7.4
	Mean+mask						6	4	5	10	1	5	5			5.2	5.0		5.1
	Median						2	9	10	7	6	10	9			6.8	9.5		8.2
	Median+mask						1	5	6	9	3	6	4			4.8	5.0		4.9
	Iterative						5	11	11	4	7	11	11			7.6	11.0		9.3
	Iterative+mask						7	6	8	3	5	3	6			5.8	4.5		5.2
	KNN						8	12	12			12	12			10.7	12.0		11.3
	KNN+mask						9	7	9			8	7			8.3	7.5		7.9
	Iterative+Bagging						11	8	3	2	9	4	10			6.6	7.0		6.8
	Iterative+mask+Bagging						10	2	2	1	8	1	2			4.6	**1.5**		**3.0**
	MIA+Bagging						12	1	1	5	10	2	1			5.8	1.5		3.6
100000	MIA						2	3		4	1					**2.5**			**2.5**
	Mean						7	8		9	5					7.2			7.2
	Mean+mask						3	5		6	2					4.0			4.0
	Median						4	9		10	6					7.2			7.2
	Median+mask						1	4		8	3					4.0			4.0
	Iterative						5	10		7	7					7.2			7.2
	Iterative+mask						6	6		5	4					5.2			5.2
	KNN																		
	KNN+mask																		
	Iterative+Bagging						9	7		1	10					6.8			6.8
	Iterative+mask+Bagging						8	2		2	8					5.0			5.0
	MIA+Bagging						10	1		3	9					5.8			5.8
Average	MIA	4.5	**1.5**	6.0	4.0	4.0	3.2	3.2	4.0	5.2	**3.8**	6.7	3.7	4.0	4.0	**3.9**	5.2	4.0	4.3
	Mean	8.5	6.5	8.0	6.0	10.0	4.5	8.8	7.0	6.5	4.2	9.0	8.0	7.5	7.8	6.2	8.5	7.5	7.5
	Mean+mask	7.0	4.0	7.0	5.5	7.0	3.8	5.2	5.0	7.8	4.2	4.3	4.7	5.0	6.1	5.2	4.5	5.0	5.2
	Median	10.0	9.5	11.0	8.0	11.0	2.8	8.8	9.7	7.0	6.0	9.3	9.0	11.0	9.9	6.8	9.2	11.0	9.2
	Median+mask	6.0	3.5	10.5	6.5	6.0	**1.8**	5.0	6.0	7.0	4.2	6.0	3.7	6.0	6.5	4.8	4.8	6.0	5.5
	Iterative	8.5	6.0	4.5	9.5	9.0	6.0	10.8	11.0	5.2	6.2	11.0	11.0	9.5	7.5	7.8	11.0	9.5	9.0
	Iterative+mask	4.5	1.5	4.5	9.5	3.0	7.0	7.0	8.7	5.0	4.2	5.0	6.0	7.5	4.6	6.4	5.5	7.5	6.0
	KNN	12.0	12.0	10.0	12.0	12.0	8.0	12.0	12.0	10.5	10.0	12.0	12.0	12.0	11.6	10.5	12.0	12.0	11.5
	KNN+mask	11.0	11.0	10.5	11.0	8.0	7.7	8.0	8.7	8.0	9.0	8.3	7.0	9.5	10.3	8.3	7.7	9.5	8.9
	Iterative+Bagging	2.5	9.0	2.0	2.5	5.0	10.0	5.5	3.0	**1.3**	8.0	3.3	10.0	3.0	4.2	5.6	6.7	3.0	4.9
	Iterative+mask+Bagging	**1.0**	6.5	**1.0**	**1.0**	**1.0**	10.0	2.0	2.0	1.7	6.8	**1.3**	2.0	2.0	**2.1**	4.5	1.7	2.0	**2.6**
	MIA+Bagging	2.5	7.0	3.0	2.5	2.0	11.5	**1.0**	**1.0**	4.0	9.2	1.7	**1.0**	**1.0**	3.4	5.4	**1.3**	**1.0**	2.8

**Table A8:** **Scores and ranks of gradient-boosted trees+MIA compared to linear methods described in TableA1**. We removed one outlier fold from one trial for the methods Linear+Iter and Linear+Iter+mask for the “task platelet_screening” at size *n*=2 500. Others are unchanged.

**(a) Scores relative to the absolute reference score and plotted in Fig. A1a**. Values in bold are the reference scores and are absolute. Other scores are given relative to the reference score of their task and size.														
	Database	Traumabase					UKBB					MIMIC		NHIS
	Task	death_screening	hemo	hemo_screening	platelet_screening	septic_screening	breast_25	breast_screening	fluid_screening	parkinson_screening	skin_screening	hemo_screening	septic_screening	income_screening
Size	Method													
2500	Boosted trees+MIA	+1e-2	+9e-3	+2e-2	+1e-1	+5e-2	+8e-2	-2e-3	+6e-2	-8e-3	+2e-2	+1e-1	+1e-1	+5e-2
	Linear+Mean	+8e-4	-3e-3	-3e-3	+8e-3	-4e-3	-6e-3	+2e-4	-1e-2	+2e-3	-6e-3	-1e-2	-2e-2	-2e-2
	Linear+Mean+mask	+6e-4	+2e-4	-2e-3	-2e-3	-4e-3	-5e-4	+1e-3	+8e-4	+2e-3	-4e-3	-2e-2	-1e-2	+7e-3
	Linear+Med	-7e-4	-8e-3	-3e-3	+6e-3	-4e-3	+2e-3	+3e-4	-1e-2	-5e-4	-5e-3	-2e-2	+1e-2	-2e-2
	Linear+Med+mask	-3e-4	-7e-3	-3e-3	-2e-3	-4e-3	+3e-3	+8e-4	+8e-4	-3e-4	-3e-3	-1e-2	+1e-2	+7e-3
	Linear+Iter	+2e-3	+4e-3	+8e-5	-5e-2	-6e-6	-1e-2	+3e-3	-2e-2	+2e-2	+9e-3	-1e-2	-3e-2	-2e-2
	Linear+Iter+mask	-2e-2	+7e-3	-5e-4	-8e-2	+1e-3	-1e-2	+2e-3	-7e-3	+2e-2	+1e-2	-2e-2	-3e-2	+3e-3
	Linear+KNN	+1e-3	-3e-3	-5e-3	+9e-3	-2e-2	-3e-2	-2e-3	-8e-3	-1e-2	-1e-2	-2e-2	-2e-2	-2e-2
	Linear+KNN+mask	+7e-4	+8e-4	-4e-3	+5e-3	-2e-2	-3e-2	-3e-3	+2e-3	-1e-2	-1e-2	-2e-2	-2e-2	+3e-3
	Reference score	**0.96**	**0.84**	**0.94**	**0.09**	**0.84**	**0.55**	**0.56**	**0.51**	**0.56**	**0.59**	**0.64**	**0.81**	**0.49**
10000	Boosted trees+MIA	+2e-2	+1e-2	+3e-2	+4e-2		+9e-2	+1e-2	+7e-2	-1e-2	+6e-2	+1e-1	+8e-2	+7e-2
	Linear+Mean	+1e-2	-9e-4	+8e-4	-4e-3		-1e-2	+8e-4	-2e-2	+2e-4	-1e-2	-1e-2	-2e-2	-2e-2
	Linear+Mean+mask	+1e-2	+4e-3	+9e-4	+1e-3		-1e-2	-2e-4	+1e-3	+7e-4	-7e-3	-2e-2	-1e-2	+1e-2
	Linear+Med	+9e-3	-6e-3	+5e-4	-6e-3		-1e-2	-7e-4	-2e-2	-5e-3	-9e-3	-8e-3	+8e-3	-2e-2
	Linear+Med+mask	+9e-3	+2e-3	+5e-4	+1e-3		-1e-2	+9e-4	+1e-3	-5e-3	-1e-2	-9e-3	+1e-2	+1e-2
	Linear+Iter	-3e-2	-1e-3	-2e-2	-1e-2		-2e-3	-3e-3	-3e-2	+3e-2	+1e-2	-2e-2	-2e-2	-4e-2
	Linear+Iter+mask	-5e-2	+5e-3	-1e-2	-6e-3		-1e-3	-3e-3	-7e-3	+3e-2	+9e-3	-7e-3	-2e-2	-6e-4
	Linear+KNN	+1e-2	-9e-3	-1e-3	-8e-3		-2e-2	-3e-3	-9e-3	-2e-2	-2e-2	-2e-2	-2e-2	-2e-2
	Linear+KNN+mask	+1e-2	-5e-3	-9e-4	-4e-3		-2e-2	-3e-3	+4e-3	-1e-2	-2e-2	-2e-2	-2e-2	+5e-3
	Reference score	**0.95**	**0.85**	**0.94**	**0.18**		**0.56**	**0.58**	**0.53**	**0.63**	**0.61**	**0.70**	**0.84**	**0.51**
25000	Boosted trees+MIA						+8e-2	+2e-2	+8e-2	-7e-3	+6e-2	+1e-1	+8e-2	
	Linear+Mean						-1e-2	+9e-4	-2e-2	-7e-3	-1e-2	-2e-2	-2e-2	
	Linear+Mean+mask						-1e-2	+1e-3	+1e-3	-7e-3	-7e-3	-1e-2	-1e-2	
	Linear+Med						-1e-2	-9e-4	-2e-2	-9e-3	-2e-2	-4e-3	+6e-3	
	Linear+Med+mask						-7e-3	-1e-3	+1e-3	-6e-3	-1e-2	-1e-2	+9e-3	
	Linear+Iter						-5e-3	-8e-3	-3e-2	+2e-2	+1e-2	-1e-4	-1e-2	
	Linear+Iter+mask						-9e-4	-8e-3	-8e-3	+2e-2	+1e-2	+1e-3	-1e-2	
	Linear+KNN						-2e-2	-2e-3	-1e-2		-4e-2	-3e-2	-2e-2	
	Linear+KNN+mask						-2e-2	-2e-3	+3e-3			-3e-2	-2e-2	
	Reference score						**0.57**	**0.59**	**0.54**	**0.69**	**0.62**	**0.73**	**0.85**	
100000	Boosted trees+MIA						+6e-2	+3e-2		+2e-2	+5e-2			
	Linear+Mean						-1e-2	-8e-4		-5e-3	-2e-2			
	Linear+Mean+mask						-8e-3	-1e-3		-3e-3	-2e-2			
	Linear+Med						-9e-3	-2e-3		-7e-3	-2e-2			
	Linear+Med+mask						-6e-3	-4e-3		-6e-3	-2e-2			
	Linear+Iter						-1e-2	-8e-3		-4e-4	+9e-3			
	Linear+Iter+mask						-1e-2	-7e-3		+4e-4	+9e-3			
	Linear+KNN							-3e-3						
	Linear+KNN+mask							-1e-3						
	Reference score						**0.60**	**0.60**		**0.74**	**0.64**			
Average	Reference score	**0.96**	**0.85**	**0.94**	**0.14**	**0.84**	**0.57**	**0.58**	**0.53**	**0.66**	**0.62**	**0.69**	**0.83**	**0.50**
														

**(b) Ranks computed from relative scores in Table A8a**. Best average ranks are in bold.																			
	Database	Traumabase					UKBB					MIMIC		NHIS	Average				
	Task	death_screening	hemo	hemo_screening	platelet_screening	septic_screening	breast_25	breast_screening	fluid_screening	parkinson_screening	skin_screening	hemo_screening	septic_screening	income_screening	Traumabase	UKBB	MIMIC	NHIS	All
Size	Method																		
2500	Boosted trees+MIA	1	1	1	1	1	1	7	1	7	1	1	1	1	**1.0**	**3.4**	**1.0**	**1.0**	**1.6**
	Linear+Mean	4	7	6	3	6	5	6	7	3	7	3	6	6	5.2	5.6	4.5	6.0	5.3
	Linear+Mean+mask	6	5	4	7	4	4	3	3	4	5	5	4	2	5.2	3.8	4.5	2.0	3.9
	Linear+Med	8	9	7	4	5	3	5	8	6	6	7	3	8	6.6	5.6	5.0	8.0	6.3
	Linear+Med+mask	7	8	5	6	7	2	4	4	5	4	4	2	3	6.6	3.8	3.0	3.0	4.1
	Linear+Iter	2	3	2	8	3	7	1	9	1	3	2	9	7	3.6	4.2	5.5	7.0	5.1
	Linear+Iter+mask	9	2	3	9	2	6	2	5	2	2	6	8	4	5.0	3.4	7.0	4.0	4.8
	Linear+KNN	3	6	9	2	9	9	8	6	9	9	9	5	9	5.8	8.2	7.0	9.0	7.5
	Linear+KNN+mask	5	4	8	5	8	8	9	2	8	8	8	7	5	6.0	7.0	7.5	5.0	6.4
10000	Boosted trees+MIA	1	1	1	1		1	1	1	7	1	1	1	1	**1.0**	**2.2**	**1.0**	**1.0**	**1.3**
	Linear+Mean	4	5	3	4		7	3	7	4	6	5	6	6	4.0	5.4	5.5	6.0	5.2
	Linear+Mean+mask	5	3	2	3		6	4	4	3	4	7	4	2	3.2	4.2	5.5	2.0	3.7
	Linear+Med	7	8	4	6		5	5	8	6	5	3	3	7	6.2	5.8	3.0	7.0	5.5
	Linear+Med+mask	6	4	5	2		4	2	3	5	7	4	2	3	4.2	4.2	3.0	3.0	3.6
	Linear+Iter	8	6	9	9		3	7	9	2	2	6	8	9	8.0	4.6	7.0	9.0	7.2
	Linear+Iter+mask	9	2	8	7		2	8	5	1	3	2	5	5	6.5	3.8	3.5	5.0	4.7
	Linear+KNN	2	9	7	8		9	6	6	9	8	9	9	8	6.5	7.6	9.0	8.0	7.8
	Linear+KNN+mask	3	7	6	5		8	9	2	8	9	8	7	4	5.2	7.2	7.5	4.0	6.0
25000	Boosted trees+MIA						1	1	1	5	1	1	1			**1.8**	**1.0**		**1.4**
	Linear+Mean						7	3	7	6	5	7	8			5.6	7.5		6.6
	Linear+Mean+mask						5	2	4	4	4	6	6			3.8	6.0		4.9
	Linear+Med						6	4	8	7	7	4	3			6.4	3.5		5.0
	Linear+Med+mask						4	5	3	3	6	5	2			4.2	3.5		3.8
	Linear+Iter						3	9	9	1	3	3	5			5.0	4.0		4.5
	Linear+Iter+mask						2	8	5	2	2	2	4			3.8	3.0		3.4
	Linear+KNN						9	6	6		8	9	9			7.2	9.0		8.1
	Linear+KNN+mask						8	7	2			8	7			5.7	7.5		6.6
100000	Boosted trees+MIA						1	1		1	1					**1.0**			**1.0**
	Linear+Mean						6	2		5	5					4.5			4.5
	Linear+Mean+mask						3	4		4	4					3.8			3.8
	Linear+Med						4	5		7	7					5.8			5.8
	Linear+Med+mask						2	7		6	6					5.2			5.2
	Linear+Iter						5	9		3	3					5.0			5.0
	Linear+Iter+mask						7	8		2	2					4.8			4.8
	Linear+KNN							6								6.0			6.0
	Linear+KNN+mask							3								3.0			3.0
Average	Boosted trees+MIA	**1.0**	**1.0**	**1.0**	**1.0**	**1.0**	**1.0**	**2.5**	**1.0**	5.0	**1.0**	**1.0**	**1.0**	**1.0**	**1.0**	**2.1**	**1.0**	**1.0**	**1.3**
	Linear+Mean	4.0	6.0	4.5	3.5	6.0	6.2	3.5	7.0	4.5	5.8	5.0	6.7	6.0	4.8	5.4	5.8	6.0	5.5
	Linear+Mean+mask	5.5	4.0	3.0	5.0	4.0	4.5	3.2	3.7	3.8	4.2	6.0	4.7	2.0	4.3	3.9	5.3	2.0	3.9
	Linear+Med	7.5	8.5	5.5	5.0	5.0	4.5	4.8	8.0	6.5	6.2	4.7	3.0	7.5	6.3	6.0	3.8	7.5	5.9
	Linear+Med+mask	6.5	6.0	5.0	4.0	7.0	3.0	4.5	3.3	4.8	5.8	4.3	2.0	3.0	5.7	4.3	3.2	3.0	4.0
	Linear+Iter	5.0	4.5	5.5	8.5	3.0	4.5	6.5	9.0	**1.8**	2.8	3.7	7.3	8.0	5.3	4.9	5.5	8.0	5.9
	Linear+Iter+mask	9.0	2.0	5.5	8.0	2.0	4.2	6.5	5.0	1.8	2.2	3.3	5.7	4.5	5.3	4.0	4.5	4.5	4.6
	Linear+KNN	2.5	7.5	8.0	5.0	9.0	9.0	6.5	6.0	9.0	8.3	9.0	7.7	8.5	6.4	7.8	8.3	8.5	7.8
	Linear+KNN+mask	4.0	5.5	7.0	5.0	8.0	8.0	7.0	2.0	8.0	8.5	8.0	7.0	4.5	5.9	6.7	7.5	4.5	6.2

### Effect of tasks' difficulty on the performance of the methods

For classification tasks, Fig. A4A shows the relative performance of the methods as a function of the tasks' difficulty. Bagged methods iterative+mask+bagging and MIA+bagging show a clear trend with lower (respectively higher) ranks for easier (harder) methods. Also, MIA is the best performing one for harder tasks (for AUC < 0.8). Thus, the interest of MIA seems more pronounced for harder tasks. There is not enough regression tasks to observe exploitable trends on Fig. A4B.
