# Supplementary material for: Benchmarking missing-values approaches for predictive models on health databases
Source: Gigascience. 2022 Apr 15;11:giac013. doi: 10.1093/gigascience/giac013 (PMC9012100; doi:10.1093/gigascience/giac013)
Supplement: giac013_GIGA-D-21-00180_Revision_1 [file giac013_giga-d-21-00180_revision_1.pdf]

GigaScience

Benchmarking missing-values approaches for predictive models on health databases.

--Manuscript Draft--

Manuscript Number:	GIGA-D-21-00180R1
Full Title:	Benchmarking missing-values approaches for predictive models on health databases.
Article Type:	Research
Funding Information:	agence nationale de la recherche (ANR-17-CE23-0018) — Dr. Gaël Varoquaux, Dr. Marine Le Morvan<br>mitacs — Mr. Alexandre Perez-Lebel<br>national institutes of health (NIH-NIBIB P41 EB019936) — Dr. Jean-Baptiste Poline<br>national institutes of health (NIH-NIMH R01 MH083320) — Dr. Jean-Baptiste Poline<br>national institutes of health (NIH RF1 MH120021) — Dr. Jean-Baptiste Poline<br>national institute of mental health (R01MH096906) — Dr. Jean-Baptiste Poline<br>canada first research excellence fund — Dr. Jean-Baptiste Poline<br>dataai — Dr. Gaël Varoquaux, Dr. Marine Le Morvan, Dr. Julie Josse

Abstract:

BACKGROUND
As databases grow larger, it becomes harder to fully control their collection, and they frequently come with missing values: incomplete observations. These large databases are well suited to train machine-learning models, for instance for forecasting or to extract biomarkers in biomedical settings. Such predictive approaches can use discriminative --rather than generative-- modeling, and thus open the door to new missing-values strategies. Yet existing empirical evaluations of strategies to handle missing values have focused on inferential statistics.
RESULTS
Here we conduct a systematic benchmark of missing-values strategies in predictive models with a focus on large health databases: four electronic health record datasets, a population brain imaging one, a health survey and two intensive care ones. Using gradient-boosted trees, we compare native support for missing values with simple and state-of-the-art imputation prior to learning. We investigate prediction accuracy and computational time. For prediction after imputation, we find that adding an indicator to express which values have been imputed is important, suggesting that the data are missing not at random. Elaborate missing values imputation can improve prediction compared to simple strategies but requires longer computational time on large data. Learning trees that model missing values --with missing incorporated attribute-- leads to robust, fast, and well-performing predictive modeling.
CONCLUSIONS
Native support for missing values in supervised machine learning predicts better than state-of-the-art imputation with much less computational cost. When using imputation, it is important to add indicator columns expressing which values have been imputed.

Corresponding Author:	Alexandre Perez-Lebel<br>Inria Saclay-Île-de-France: Inria Centre de Recherche Saclay-Ile-de-France<br>Palaiseau, FRANCE
Corresponding Author Secondary Information:	
Corresponding Author's Institution:	Inria Saclay-Île-de-France: Inria Centre de Recherche Saclay-Ile-de-France
Corresponding Author's Secondary Institution:	
First Author:	Alexandre Perez-Lebel

Powered by Editorial Manager® and ProduXion Manager® from Aries Systems Corporation

**First Author Secondary Information:**	
**Order of Authors:**	Alexandre Perez-Lebel
	Gaël Varoquaux
	Marine Le Morvan
	Julie Josse
	Jean-Baptiste Poline
**Order of Authors Secondary Information:**	
**Response to Reviewers:**	Response to Reviewers

Alexandre Perez-Lebel, Gaël Varoquaux, Marine Le Morvan, Julie Josse, Jean-Baptiste Poline

We sincerely thank the reviewers for their time and feedback. This feedback led us to add significant material to the manuscript, and we believe that the resulting findings are interesting and add value to the study. Below we reply to the various points raised by the reviewers, quoting the questions and suggestions, and provide answers to complement and explain the changes that we made to the manuscript.

Response to Reviewer #1:

> Many of the imputation methods used to compare to MIA aren't commonly used in practice, such as what's called here "constant imputation", using the mean or median across all of a measured feature to substitute for those missing. Also, the current standard in the field (at least for inference) is multiple imputation, which doesn't appear to have been used. It's not clear if this is one of the methods described as "conditional imputation", but it would make sense to include this method, given its wide use, despite the computational burden.

This comment echoes comment #2 of the other reviewer. Thus for this important question, we kindly ask the reviewer to look at our answer to comment #2 of the other reviewer.

> In figure 1, MIA appears favorable at n=100000, but not so much at n=25000 or n=10000. Can the authors explain this? The imputation methods with their evaluation metrics should be evaluated separately at each n, not just overall.

The Wilcoxon one-sided signed-rank test given in Supplementary Figure 4 shows that MIA is significantly favorable at level 0.05 against all other non-bagged methods at size n=2500. The higher the number of samples, the less significant are the results because the less tasks are available (indeed, only a small number of our datasets have more than 100000 samples). As the reviewer pointed out in the next comment, this is not clear to the reader when looking at Figure 1 so we added the results of the Wilcoxon test directly on Figure 1, as explained in the next comment.

> Since some tasks are only examined at certain n, the different n are not comparable although they are shown side by side. This is very misleading.

As each bar represents the difference to the mean for a given task (and given n), the fact that there are different tasks for different n does not lead to a systematic difference. Yet, as the reviewer points out, an important difference is that statistical power is not the same across sizes due to the varying number of tasks available at each size. To make it clearer for the reader, we added the results of the Wilcoxon one-sided signed-rank test directly on the results figures (Figure 1 and Supplementary Figure 2). Methods which performed significantly poorer (resp. better) at level 0.05 are marked with "*" (resp. "*(>)") and "**" (resp. "**(>)") for Bonferroni-corrected levels. By doing so, the reader can qualify his or her analysis of the results by seeing that some results are or are not significant at the considered size.

> It seems that the info in figure 1 is an aggregate across all the datasets and tasks that were completed. Can it be shown broken out by data and task? Or can it be

tested whether there were differences across data and task? The best method may vary by task. And if there were one very difficult task it would likely not be well represented here. A difficult task might merit more careful imputation, or not. Even though these are shown in the appendix, the tables are very dense and painful to examine.

The reviewer is correct that there is “no free lunch” in machine learning, and it is well known that there is no universally optimal predictor. Following the reviewer’s advice, we added Supplementary Figure 7 to better visualize scores obtained by the methods across tasks, showing scores of Figure 1 broken out by task. By doing so, we can see how each method compares to each other and observe the variabilities of the methods across tasks.
Regarding difficulties of the tasks, the reviewer highlights an interesting point: performances of the methods may vary depending on the difficulty of the task. To investigate this point, we added Supplementary Figure 5 showing the rank of each method as a function of the mean score obtained by all the methods on the task. The mean score is taken here as a proxy of the difficulty of the task: we assume that easier (resp. difficult) tasks have a high (resp. low) mean score. Local regressions (LOWESS) curves are plotted to spot trends. This figure highlights interesting trends : for some methods, the relative performance does not depend much on the difficulty of the task while others have strong trends. We added a paragraph highlighting these trends next to Supplementary Figure 5 in the appendix.

> I find the relative prediction score difficult to interpret, the actual quality of any prediction isn't recoverable. Also if most of these data are MNAR, as the authors suggest, there might be very little predictive accuracy for any of these methods.
If the results were broken out by task, then more standard measures of prediction accuracy like the AUC could be used instead of the relative prediction score, which is hard to interpret. Regardless of which metric is used (relative prediction score or AUC or coefficient of determination), the variability should be shown somehow, not just the average.

Supplementary Figure 7 added in response to the previous comment that shows scores broken out by task also partly answers this comment.
The reviewer raises the important point that for results to be relevant, the methods should have a reasonable predictive accuracy. As visible on Supplementary Table 7, and newly added Supplementary Figure 7, we were careful not to benchmark tasks with predictive accuracies close to chance. 5 of the 10 classification tasks have an AUC above 0.8, 9 are above 0.6 and one, the worst performer has an AUC of 0.59 which is poor but still way above chance. 2 of the 3 regression tasks have a R2 above 0.5 and one, the worst performer has a R2 of 0.17.
Variability can now be read on newly added Supplementary Figure 7. Absolute predictive accuracies can also be read in Supplementary Table 8a.

> The description of the nested cross-validations is a bit confusing. What exactly is the outer cross validation that "averages the validations of the tuned model"? Do they mean averaging the predictions? This is unclear.

We rephrased and added more details in the second paragraph of the “Experiment” section page 7.

> Presumably the same cross-validation and training sets were used across methods, but this should be stated explicitly.

We added a sentence at the end of the first paragraph of the “Experiment” section page 7 to clarify.

> Judging from figure 5, there are many features incorporated that have 80% or more incompleteness. That is a very difficult situation for extracting value from these features, did the authors look at whether it is worth the effort to include such data?

To measure the feature importance and its relation with the proportion of missing values in the features, we ran permutation feature importance for each task for the method MIA. Once the method is fitted, to evaluate the importance of a feature, we

Powered by Editorial Manager® and ProduXion Manager® from Aries Systems Corporation

randomly permute this feature 10 times and each time compute the drop in score which is then averaged. The higher the score drop the more important is the feature. We added Supplementary Figure 8 in which we plotted the feature importance (in terms of average score drop) as a function of the proportion of missing values in the feature. We observe no marked association between the proportion of missing values in the feature and its importance, arguing that all features are important for the prediction, even the ones with a high proportion of missing values (e.g 80%).

Minor comments
> Page 3- "exposed in the appendices"- should this be "described in the appendices"?

Indeed, we fixed the typo.

> Figure 1. Why aren't KNN and KNN+mask shown for n=100000?

KNN and KNN+mask were intractable at n=100000 in terms of memory usage: KNN has a $O(n^2)$ memory footprint (with 64-bit floats, 80GB are required).
To make it clearer, we added this information in Figure 1 and in its caption.

Response to Reviewer #2:

Summary:
The paper provides a rigorous empirical evaluation of imputation methods and native support of missing values via decision trees for downstream prediction tasks (classification and regression) on large-scale health datasets. The strengths of the paper include its originality in evaluating methods on large-scale health data, whereas previous evaluations focus on smaller-scale benchmark datasets and a principled evaluation based on significance tests on the relative rank of methods. As discussed more fully below, the paper does not appropriately discuss in the introductory section previous empirical evaluations of imputation methods for supervised learning. The paper should address in the section entitled 'Limitations and further work' two limitations of the study: (1) the evaluation metric focuses on accuracy in individual predictions rather than the capturing the multivariate distributional features of the data; and (2) the evaluations do not capture uncertainty of imputing missing values conditional on the observed values.

We thank the reviewer for recognizing that a major strength of this work is its evaluation of methods on large scale and real-world health databases whereas previous works have focused on much smaller datasets. We truly believe that this is what makes this work different and useful for the community.

Major recommendations:
> 1. It is claimed in the abstract and introduction that there is little or no existing empirical evaluations of imputation methods on downstream predictive tasks. There are in fact several existing evaluations of imputation methods based on downstream predictive tasks:
• Bertsimas et al. (2017, Journal of Machine Learning Research)
• Poulos and Valle, (2018, Applied Artificial Intelligence)
• J¨ager et al. (2021, Frontiers in Big Data)
The first and third papers listed also evaluates in terms of imputation quality. The second paper compares different combinations of imputation methods and predictive models in terms of downstream predictive performance, focusing on classification tasks only. The authors should discuss in the introductory section the differences between these evaluations and their work. I suspect a major difference is that the present study focuses on larger-scale medical data with many weakly correlated features, whereas the previous studies use smaller-scale machine learning "benchmark" datasets with varying average correlations between features.

We thank the reviewer for pointing us to these works which we were unaware of. We have taken them into account in the revised version of our manuscript and cited them as prior works in the introduction. Indeed, one of the major differences between these

Powered by Editorial Manager® and ProduXion Manager® from Aries Systems Corporation

works and ours is the type of data used for benchmarking. Some of these works use rather small scale benchmark datasets with simulated (or artificially perturbed) missing values while we use large scale health databases with ‘real’ missingness (i.e missing values already present in the real datasets). Another important difference with some of these works is that we consider predictive models directly handling missing values.

> 2. There are two additional limitations that should be discussed in ‘Limitations and further work’: the evaluations are based on a single 1 performance metric that focuses on the accuracy of individual predictions rather than (1) distributional features of multivariate data or (2) the repeating sampling properties of the imputation methods. Regarding the first limitation, Little and Rubin (2014) argue that imputation methods should be evaluated on their ability to preserve the distributional characteristics of the data. Regarding the second limitation, Rubin (1996) argues that comparing imputed values to a single ground truth value fails to account for the uncertainty in imputing the missing values conditional on the observed data. A working paper by Wang et al. (2021) demonstrates why relying on a single performance metric and a single dataset (rather than repeatedly drawn samples of a dataset) may be misleading.

The reviewer touches on two crucial points here, which we address below.
The first point mentions that “imputation methods should be evaluated on their ability to preserve the distributional characteristics of the data”. In our paper, we do not assess the imputation quality of the various imputation techniques used. However, this is intended as the goal of the paper is to assess different schemes to predict in the presence of missing values, which is measured in terms of regression performance and classification accuracy. In particular, assessing the ability of an imputation method to preserve the distributional characteristics of the data does not provide a proxy for assessing its quality in prediction settings. Indeed, it has been shown recently that Impute-then-Predict procedures are Bayes optimal whatever the missing data mechanism, and whatever the choice of imputation function (reference LeMorvan 2021 in the manuscript). In other words, if the train and test sets are imputed using the same imputation functions, then a flexible predictor will provide optimal performances asymptotically. This result highlights the fact that “good” imputation (in terms of preservation of the moments of the distribution for example) is not necessary for good prediction, at least asymptotically. Moreover, it also highlights the fact that using Multiple Imputation to reflect the uncertainty in imputed values is not necessary either, which brings us to the second point. This is at odds with the results of Little and Rubin, because the settings of Little and Rubin are quite different: Little and Rubin formulate a probabilistic model of the fully-observed data and ask how to recover this probabilistic model in the presence of missing values. However, here 1) we make no hypotheses with regards to the form of a probabilistic model of the data 2) the best predictor in the presence of missing values may not be the same as that for fully-observed data (theoretical discussions of this last point are made in Josse 2019 and LeMorvan 2021).
The second point mentions that using single imputation instead of Multiple Imputation results in a failure of taking into account the uncertainty in the imputed values. This is true and it is indeed important for inference purposes to use Multiple Imputation or Bootstrapping to capture the uncertainty in the estimates due to missing values. However, again, the theoretical result mentioned above highlights the fact that it is not necessary to reflect the uncertainty in the imputed values to obtain optimal prediction performances.

Since the problem of imputation or inference is much more studied than that of supervised learning with missing values, it is natural to wonder whether the good practices in inference are also the ones that should be used for predictions. We therefore added a discussion about these points in the paper (paragraph “Multiple Imputation” page 3 and first paragraph of the “Interpretation” section page 6).

Moreover, as the previously mentioned theoretical results are true asymptotically, the question of whether or not Multiple Imputation is beneficial in finite-samples regimes remains. Thus, following the question of the two reviewers, we did additional experiments with Multiple imputation in the context of predictions. One challenge is that the notion of Multiple Imputation is not clearly defined in the context of predictive models (supervised learning), because it needs to be applicable in an out-of-sample setting: applying the predictive models on new data. Theoretically, the only result that we are aware of for Multiple Imputation in the context of prediction requires access to

Powered by Editorial Manager® and ProduXion Manager® from Aries Systems Corporation

an oracle predictor for fully observed data and is valid only in MAR (Josse 2019, theorem 3). A tool closely-related to Multiple Imputation is that of the Bootstrap, which is used to obtain variances and gauge uncertainty with complicated estimators, including for Multiple Imputation. In predictive modeling, Bootstrap is classically used in the context of “bagging”: Bootstrap aggregating, where multiple predictors are learned on bootstrap replicates and the prediction is an average of the prediction of each predictor. From the practical side, Khan et al 2019 review and compare a number of methods for using the Bootstrap and Multiple Imputation in the context of Ensemble approaches. It comprises the following methods: Learning on an averaged version of a multiply imputed dataset, Bagging single imputations, Bagging multiple imputations, constructing ensembles based on predictors that were each learned on a version of a multiply imputed dataset, etc. Since it is neither possible, nor the goal of this paper to compare all the ways to use Multiple Imputation in the context of predictions instead of inference, we chose one option: Bagging single imputation.

We thus added 3 new methods in the main experiment : Iterative+Bagging, Iterative+mask+Bagging and MIA+Bagging. By seeing the improvement brought by the two first methods, we suspected it was rather the effect of the Bagging than the effect of Multiple Imputation. To assess this claim, we ran MIA+Bagging and got as good results as Iterative+mask+Bagging, which confirmed the claim (as shown in Figure 1). To be sure that it was not an effect of an implicit multiple imputation when using MIA+Bagging, we ran a fourth method Mean+Mask+Bagging, which obtained as good results and confirmed the fact that the improvement was an effect of the Bagging and not the effect of Multiple Imputation. Supplementary Figure 9 in the supplementary materials best summarizes these findings.
Note that these methods were very costly and this addition increased our total CPU time from 12 000 hours to 500 000 CPU hours, i.e 1 CPU for 60 years, which is a strong limitation of this part of our study. Not every lab could have afforded to run such a study.

We thoroughly rewrote the findings paragraph to integrate the new results coming from the experiments with multiple imputation and bagging. The new experiments also increased the correction for multiple comparisons in some of the statistical tests, which led to some differences no longer being significant.

Minor recommendations
> 1. pg. 2, middle left: the list of references starting with Miotto et al. 2016, should be in parentheses

Fixed.

> 2. pg. 3, top left: clarify that missing values in the test set should be imputed with the training set mean or median

We added a sentence to clarify.

> 3. pg. 3, top right: clarify that the feature sets should be the same within each predictive task. The concern is that if the ANOVA variable selection procedure yields different feature sets within a task, it would bias the comparison between imputation methods.

We added a sentence to clarify.

> 4. pg. 4, top right: “train datasets” → “training datasets”

Fixed.

> 5. pg. 8, bottom right: more information on how to access protocols.io

We added a link to the protocol in the “Availability of supporting data and material” section and added a reference to this link in the “Experiment” section.

> 6. pg. 11, Table 6 caption: typo: “larger that”

Powered by Editorial Manager® and ProduXion Manager® from Aries Systems Corporation

	Fixed.
**Additional Information:**	
**Question**	**Response**
Are you submitting this manuscript to a special series or article collection?	No
**Experimental design and statistics**<br><br>Full details of the experimental design and statistical methods used should be given in the Methods section, as detailed in our Minimum Standards Reporting Checklist. Information essential to interpreting the data presented should be made available in the figure legends.<br><br>Have you included all the information requested in your manuscript?	Yes
**Resources**<br><br>A description of all resources used, including antibodies, cell lines, animals and software tools, with enough information to allow them to be uniquely identified, should be included in the Methods section. Authors are strongly encouraged to cite Research Resource Identifiers (RRIDs) for antibodies, model organisms and tools, where possible.<br><br>Have you included the information requested as detailed in our Minimum Standards Reporting Checklist?	Yes
**Availability of data and materials**<br><br>All datasets and code on which the conclusions of the paper rely must be either included in your submission or deposited in publicly available repositories (where available and ethically appropriate), referencing such data using a unique identifier in the references and in the "Availability of Data and Materials" section of your manuscript.	Yes

Powered by Editorial Manager® and ProduXion Manager® from Aries Systems Corporation

Have you have met the above requirement as detailed in our Minimum Standards Reporting Checklist?	

Manuscript Click here to access/download;Manuscript;manuscript.pdf

OXFORD (GIGA)^n SCIENCE

GigaScience, XXXX, 1–23

doi: xx.xxxx/xxxx
Manuscript in Preparation
Paper

PAPER

Benchmarking missing-values approaches for predictive models on health databases.

Alexandre Perez-Lebel[1,2,3,*], Gaël Varoquaux[1,2,3], Marine Le Morvan[2], Julie Josse[4] and Jean-Baptiste Poline[1]

[1]McConnell Brain Imaging Centre, The Neuro (Montreal Neurological Institute-Hospital), Faculty of Medicine, McGill University, Montreal, QC, Canada and [2]Inria, Palaiseau, France and [3]Mila, Montreal, QC, Canada and [4]Inria, Montpellier, France

[*]alexandre.perez@inria.fr

Abstract

BACKGROUND As databases grow larger, it becomes harder to fully control their collection, and they frequently come with missing values: incomplete observations. These large databases are well suited to train machine-learning models, for instance for forecasting or to extract biomarkers in biomedical settings. Such predictive approaches can use discriminative –rather than generative– modeling, and thus open the door to new missing-values strategies. Yet existing empirical evaluations of strategies to handle missing values have focused on inferential statistics. **RESULTS** Here we conduct a systematic benchmark of missing-values strategies in predictive models with a focus on large health databases: four electronic health record datasets, a population brain imaging one, a health survey and two intensive care ones. Using gradient-boosted trees, we compare native support for missing values with simple and state-of-the-art imputation prior to learning. We investigate prediction accuracy and computational time. For prediction after imputation, we find that adding an indicator to express which values have been imputed is important, suggesting that the data are missing not at random. Elaborate missing values imputation can improve prediction compared to simple strategies but requires longer computational time on large data. Learning trees that model missing values –with missing incorporated attribute– leads to robust, fast, and well-performing predictive modeling. **CONCLUSIONS** Native support for missing values in supervised machine learning predicts better than state-of-the-art imputation with much less computational cost. When using imputation, it is important to add indicator columns expressing which values have been imputed.

Key words: Missing values; machine learning; supervised learning; benchmark; imputation; multiple imputation; bagging

Background: missing values in databases

Missing values are pervasive in many application domains. This is particularly true on health data, where missing values arise for a multitude of reasons: two patients rarely follow the same medical path and take the exact same set of exams; measurements are omitted because of lack of time or because the patient's condition does not allow it; hospitals do not collect exactly the same information because of diverging practices and the use of different devices; etc. This problem is exacerbated when the data are aggregated across multiple sources or when each individual sample comprises many features. The more data there is, the more data is missing.

There is a rich and established statistical literature for the treatment of missing data (Little and Rubin; 2019; Wells et al.; 2013), which has so far been mostly focused on inferential purposes, *i.e.* estimating parameters of a probabilistic model with their confidence intervals. For such problem, an important distinction between missing data mechanisms was introduced by Rubin (1976): Missing Completely At Random (MCAR) where the probability of having missing data does not depend on the covariates, Missing At Random (MAR), where the probability of

Compiled on: November 30, 2021.
Draft manuscript prepared by the author.

Key Points

- Benchmarks on health databases highlight the challenges that they represent for statistical learning: non-ignorable missing values (Missing Not At Random – MNAR), non-linear relationships between covariates and outcomes.
- Native missing-values support in supervised machine learning gives better prediction than state-of-the-art imputation with significantly less computational cost.
- With linear models, conditional imputation is to be preferred.
- When using imputation, concatenating the missingness indicator with the input features significantly improves predictions.
- Bagging, as sometimes used for multiple-imputation, improves prediction performance but with a prohibitive time cost.

a missing value only depends on the observed values of other variables; and Missing Not At Random (MNAR) which covers all other cases. MNAR corresponds to cases where the missingness carries information. For example, if heartbeat measures are not reported when the values are too low, it creates a MNAR situation. Most available methods for inference in the presence of missing values are only valid under the MAR assumption, including maximum likelihood approaches with the Expectation Maximization algorithm (Dempster et al.; 1977), as well as Multiple Imputation (Van Buuren; 2018). The latter is a two-step approach where the data is first imputed multiple times to create multiple completed datasets, and then the analysis is performed on each imputed dataset separately before combining the results to take into account the uncertainty due to missing values.

Supervised learning to build models that predict best a response using covariates with missing values can lead to different tradeoff than inference models (Sperrin et al.; 2020; Josse et al.; 2019). In health, such predictive models are central to building complex biomarkers or risk scores, to forecasting an epidemic, and they can even underlie causal inference for policy evaluation (Rose and Rizopoulos; 2020). They are increasingly used on electronic health records (Miotto et al.; 2016; Zheng et al.; 2017; Steele et al.; 2018), where the choice of strategy to handle missing values remains a challenge (Jarrett et al.; 2021). Indeed, unlike with inference, little work to date has focused on the systematic evaluation of supervised learning with missing values. Existing works focus on benchmarking imputation quality (Jäger et al.; 2021; Bertsimas et al.; 2018) – which, as our study points out, is a different goal than prediction quality – or only focus on imputation-based methods (Poulos and Valle; 2018).

In practice, a number of options are commonly used to learn predictive models with missing values. The simplest one is to delete all observations containing missing values. However, leaving aside the possible biases that this practice may induce, it often leads to considerable loss of information in high and even moderate dimensions. Indeed, when there are many variables, it is common that only a few observations are completely observed.

In order to deal with arbitrary subsets of input features, the most common practice currently consists in first imputing the missing values, and then learning a predictive model (e.g regression or classification) on the completed data. The popularity of this approach is mainly due to its simplicity and ease of implementation. After imputation, off-the-shelf learners can be applied on the completed dataset. Recent theoretical results show that applying a supervised-learning regression on imputed data can asymptotically recover the optimal prediction function; however most imputation strategies, including the common imputation by the conditional expectation, create discontinuities in the regression function to learn (Le Morvan et al.; 2021).

A small number of machine learning models can natively handle missing values, in particular popular tree-based methods. Trees greedily partition the input space into subspaces in order to minimize a risk. This non-smooth optimization scheme enables them to be easily adapted to directly learn from incomplete data. Several adaptations of trees to missing values have been proposed (see Josse et al.; 2019, for a short review). Missing Incorporated in Attributes (MIA, Twala et al.; 2008) is the most promising strategy (Josse et al.; 2019), described below in the experiment section.

In this work, we benchmark the most popular methods for supervised learning with missing values on multiple large real-world health databases. In contrast to most simulations, real health databases combine a number of challenges: unknown data distributions (not necessarily Gaussian), uncontrolled missing data mechanism (not necessarily MAR), mixed quantitative and categorical data, and often a high level of noise. In such a challenging setting, we compare existing approaches to make recommendations that are directly relevant for the practitioner. To establish general recommendations, we study a total of 13 prediction *real-world* tasks (10 classification and 3 regression tasks) across four publicly-available health databases of very different nature. For each of these tasks, we compare methodologies based on imputation followed by regression or classification, to tree-based models that can natively handle missing values with a MIA strategy. These methods are chosen from the common practice as well as theoretical work on supervised learning with missing values (Josse et al.; 2019).

The present study has several strengths in terms of benchmarking methodology, avoiding common limitations. It uses real data *and* real missingness; multiple draws of a cross-validation loops are used; the imputation procedure is not fitted on the whole dataset but rather on the training set to prevent leaks from the training set to the out-of-sample test set; hyper-parameters of the predictive model are tuned for each method to reduce bias in the hyper-parameters selection; and finally the study benchmarks imputation methods *and* predictive models that handles missing values. As a result, our benchmark is very computation-intensive: the whole study costed approximately 520 000 CPU hours, *i.e.* 60 years on a single CPU, revealing the need to also account for compute cost in recommendations.

After briefly exposing our benchmarking methodology, we give a synthetic view of the findings and discuss observed trends. Overall, the benchmarks reveal the presence of missing not at random (MNAR) values and non-linear mechanisms. High-quality conditional imputation gives good prediction provided that a variable indicating which entries were imputed is added to the completed data. However, its algorithmic complexity makes it prohibitively costly on large data. Rather, tree-based methods with integrated support for missing val-

ues (missing incorporated attribute – MIA) perform as well or better, at a fraction of the computational cost.

Empirical study

Benchmarking the imputation and MIA methods

Our experiments compare two-step procedures based on imputation followed by regression or classification, as well as tree-based models with an intrinsic support for missing values thanks to MIA. The 12 methods compared are summarized in Table 1: MIA, 8 methods based on single imputation and 3 methods using Multiple Imputation via Bagging. Below, we describe further the imputation strategies benchmarked as well as MIA.

Single Imputation

Constant imputation: mean and median. The simplest approach to imputation is to replace missing values by a constant such as the mean, the median or the mode of the corresponding feature. This is frowned upon in classical statistical practice, as the resulting data distribution is severely distorted compared to that of fully-observed data. Yet, in a supervised setting, the goal is different from that of inferential tasks. Recent theoretical results have established that powerful learners such as ones based on trees can learn to recognize such imputed values and give the best possible predictions (Josse et al.; 2019). The key to the success of this strategy is to impute the training and the test set with the same constant: missing values of the test set are imputed with the constants learned on the training set (mean, median, etc).

Conditional imputation: MICE and KNN. Powerful imputation approaches rely on conditional dependencies between features to fill in the missing values. Adapting machine-learning techniques gives flexible estimators of these dependencies. Classical approaches include k-nearest neighbor regressors (Chen and Shao; 2000), and iterative conditional imputers that predict one feature as a function of others, as with the MICE imputer (Buuren and Groothuis-Oudshoorn; 2010). In our experiments, we benchmark their implementation in scikit-learn (Pedregosa et al.; 2011): the `KNNImputer` as well as the `IterativeImputer`, using linear models to impute missing values.

Adding the mask. Conditional imputation can make it hard for the learner to retrieve which entries were originally observed and which were originally missing. However, the information of missingness can be relevant for predicting the outcome in cases where it depends on missingness, or in *missing not at random* settings where the missingness carries information. For these reasons, it can be useful after imputation to add new binary features that encode whether a value was originally missing or not: the *mask* or *missingness indicator* (Josse et al.; 2019; Sharafoddini et al.; 2019; Sperrin et al.; 2020).

Table 1. Methods compared in the main experiment.
All use gradient-boosted trees as predictive model. 10 use imputation and 2 uses MIA. Bagging uses 100 estimators in the ensemble.

In-article name	Imputer	Mask	Bagging	Predictive model
MIA	–	No	No	Boosted trees
Mean	Mean	No	No	Boosted trees
Mean+mask	Mean	Yes	No	Boosted trees
Median	Median	No	No	Boosted trees
Median+mask	Median	Yes	No	Boosted trees
Iterative	Iterative	No	No	Boosted trees
Iterative+mask	Iterative	Yes	No	Boosted trees
KNN	KNN	No	No	Boosted trees
KNN+mask	KNN	Yes	No	Boosted trees
Iterative +Bagging	Iterative	No	Yes (100)	Boosted trees
Iterative+mask +Bagging	Iterative	Yes	Yes (100)	Boosted trees
MIA+Bagging	–	No	Yes (100)	Boosted trees

Multiple Imputation

When estimating model parameters, it is of great importance to reflect the uncertainty due to the missing values. For this purpose, Multiple Imputation methods are widely used, often via Resampling methods such as the Bootstrap. However, for prediction (classification or regression) theoretical conditions differ from that of parameters estimation. Indeed, it has been shown recently that a sufficiently flexible learner reaches optimal performances asymptotically with Single Imputation, whatever the missing data mechanism and whatever the choice of imputation function (Le Morvan et al.; 2021). Still, this result holds in asymptotic regimes, and there is a need for empirical results on handling missing values with Multiple Imputation or Bootstrap in the context of supervised learning. Theoretically, the only result that we are aware of for Multiple Imputation in the context of prediction requires access to an oracle predictor for fully observed data and is valid only in MAR (Josse et al.; 2019, th. 3). In general, it is not clear how to use Multiple Imputation for supervised learning: sampling can be applied in different ways during training the model or applying their predictions to new data. Khan et al. (2019) review and compare a number of methods for using Multiple Imputation and Bootstrap: learning on an averaged version of a multiply imputed dataset, bagging single imputations, bagging Multiple Imputations, constructing ensembles based on predictors that were each learned on a version of a multiply imputed dataset (Friedman et al.; 2001, chap 16). As these methods all come with a significant computing cost, we focus on the most promising approach: bagging single imputation. More precisely, we draw for each task 100 bootstrap replicates. We then fit the single imputation and the predictive model on each of these replicates, to obtain 100 predictors. Final predictions are made either by voting or by averaging (see Supplementary Table 5).

Directly handling missing values with tree-based models: MIA

We also consider the MIA (Missing Incorporated in Attribute) strategy to readily model missing values in tree-based models. It has the benefit of using all samples, including incomplete ones, to produce the splits of the input space. More precisely for each split based on variable j, all samples with a missing value in variable j are either sent to the left or to the right child node depending on which option leads to the lowest risk. Note that the samples with an observed value in variable j can either be split between the left and right child node according to whether their values x_j is greater or smaller than a threshold, or either all be sent to the same child node so that they are separated from the samples with a missing value in variable j. That makes MIA particularly suited to Missing Not At Random (MNAR) settings, as it can harness the missingness information. Moreover, since trees with MIA directly learn with missing values, they provide a straightforward way of dealing with missing values in the test set. We use the implementation in scikit-learn's boosted trees (`HistGradientBoostingRegressor`).

Predictive model

For the supervised learning step, we focus on gradient-boosted trees –though we also benchmark linear models in a complementary analysis described in the appendices. We applied supervised learning to the imputed data for the imputation-based

methods. We also used the tree models with their support of MIA for a direct handling of missing values. Gradient-boosted trees are state-of-the art predictors for tabular data (Chen and Guestrin; 2016; Olson et al.; 2018; Shwartz-Ziv and Armon; 2021), and thus constitute a strong baseline. Moreover, using gradient-boosted trees enables us to keep the same predictive model for all approaches, thereby putting emphasis on the impact of the missing data treatment.

To define the input features we either use the choice of experts in prior studies, or feature screening, a classic machine-learning procedure using a simple ANOVA-based univariate test of the link of each feature to the outcome (Guyon and Elisseeff; 2003). In both cases, the same set of selected features is used for all methods within each predictive task. Selecting features is necessary because some of the imputation methods studied are not tractable with a large number of features.

Health databases

To reach conclusions as general as possible we used four real-world health-related databases. These databases vary in terms of location, size, purpose and time, to cover a wider data scope. These databases already existed and no data collection was made in this study. Below, we describe them briefly, giving the prediction tasks studied for each of them.

Traumabase

The Traumabase Group is a collaboration studying major trauma. The database gathers information from 20 French trauma centers on more than 20 000 trauma cases from admission until discharge from critical care. Data collection started in 2010 and is still ongoing in 2020. We used records spanning from 2010 to 2019. Data can be obtained by contacting the team on the Traumabase website (www.traumabase.eu).

We defined 5 prediction tasks on this database, 4 classifications and 1 regression. Outcomes are diverse: patient's death, hemorrhagic shock, septic shock, and platelet count. Features for the hemorrhagic shock prediction are taken from Jiang et al. (2020).

UK Biobank

UK Biobank (UKBB) (Sudlow et al.; 2015) is a major prospective epidemiology cohort with biomedical measurements. It provides health information on more than 500 000 United-Kingdom participants aged between 40 to 69 years from 2006 to 2010. The data are available upon application as detailed on the UK BioBank website (www.ukbiobank.ac.uk).

We defined 5 tasks on this database, 4 classifications and 1 regression. Outcomes are the diagnosis of three diseases - breast cancer, skin cancer, Parkinson's disease - as well as prediction of the fluid-intelligence score. Breast cancer prediction uses features defined in Läll et al. (2019).

MIMIC-III

The Medical Information Mart for Intensive Care (MIMIC) database (Johnson et al.; 2016) is an Intensive Care Unit (ICU) dataset developed by the MIT Lab for Computational Physiology. It comprises deidentified health data associated with about 60 000 ICU admissions recorded at the Beth Israel Deaconess Medical Center of Boston, United States, between 2001 and 2012. It includes demographics, vital signs, laboratory tests, medications, and more. The data can be accessed via an application described on the MIMIC website (mimic.physionet.org).

We defined 2 classification tasks on this database. Outcomes are septic shock and hemorrhagic shock.

NHIS

The National Health Interview Survey (NHIS) (National Center for Health Statistics; 2017) is a major data collection program of the National Center for Health Statistics (NCHS), part of the Centers for Disease Control and Prevention (CDC) in the United States. It aims to monitor the health of the population. Since 1957, it collects data from United-States population. We use the 2017 edition, summing up to approximately 35 000 households containing about 87 500 persons. The database is freely-accessible on the NHIS website (www.cdc.gov/nchs/nhis/).

We defined 1 regression task on this database. Outcome is the yearly income.

More details on each database and task can be found in the appendices, in particular in Supplementary Table 7 and Supplementary Figure 4 that detail the number of features available and their type (numerical, ordinal and categorical), and Supplementary Figure 6 giving the distribution of missing values across features.

Findings

Figure 1 summarizes the performances and computational times of the various methods across the 4 databases and 13 prediction tasks. To explore the importance of the amount of data, we created training datasets of 4 sizes: 2 500, 10 000, 25 000 and 100 000 samples. We report the general trends.

Bagging improves prediction, MIA performs well at limited cost

Iterative+mask+Bagging obtains the best overall average rank (2.6) across all tasks and sizes in terms of prediction score closely followed by MIA+Bagging (2.8) as shown on Figure 1a and Supplementary Table 8b. Overall, Bagging improves markedly all approaches (supplementary Figure 9). However the cost of these bagged methods can be prohibitive. At size n=100 000, Iterative+mask+Bagging and MIA+Bagging cost 369 and 117 CPU days per task respectively, about 100 to 200 times slower than non-bagged method such as MIA (1.9 CPU days per task).

MIA enables to navigate a trade off between prediction performance and computational tractability: With Bagging it comes close to Iterative+Mask with half the computational cost on large databases. Without Bagging, it is the best overall performer, with an overall average rank of 4.3, and up to 200 times faster. It is followed by Mean+mask, Median+mask and Iterative+mask with overall average ranks of 5.2, 5.5 and 6.0 respectively. Mean, KNN+mask, Iterative, Median and KNN performed the worst with overall average ranks of 7.5, 8.9, 9.0, 9.2 and 11.5 respectively. Supplementary Table 8a and 8b give more quantitative details about scores and ranks of each method.

Similar observations can be made on each size separately. MIA obtained the best prediction scores on every size with average ranks of 4.3, 4.6, 4.4 and 2.5 on sizes 2 500, 10 000, 25 000 and 100 000 respectively as shown on Supplementary Figure 3a.

In terms of computing time, beyond the fact that Bagging multiplies by 100 the cost of every method, MIA is almost always the fastest (Figure 1b), though it gives excellent prediction performance. It is on par with Mean, and Median imputations, but adding the mask to these methods –a key ingredient to prediction performance– doubles their computing times. At the other end of the spectrum, Iterative+mask and KNN+mask are the slowest non-bagged methods. The gaps between training times of the methods increase with the size of the database, revealing the difference in algorithmic scalability.

Statistical significance. To assess significance of the above results, we ran three statistical tests: the Friedman test (Friedman; 1937, 1940), the Nemenyi test (Nemenyi; 1963) and the

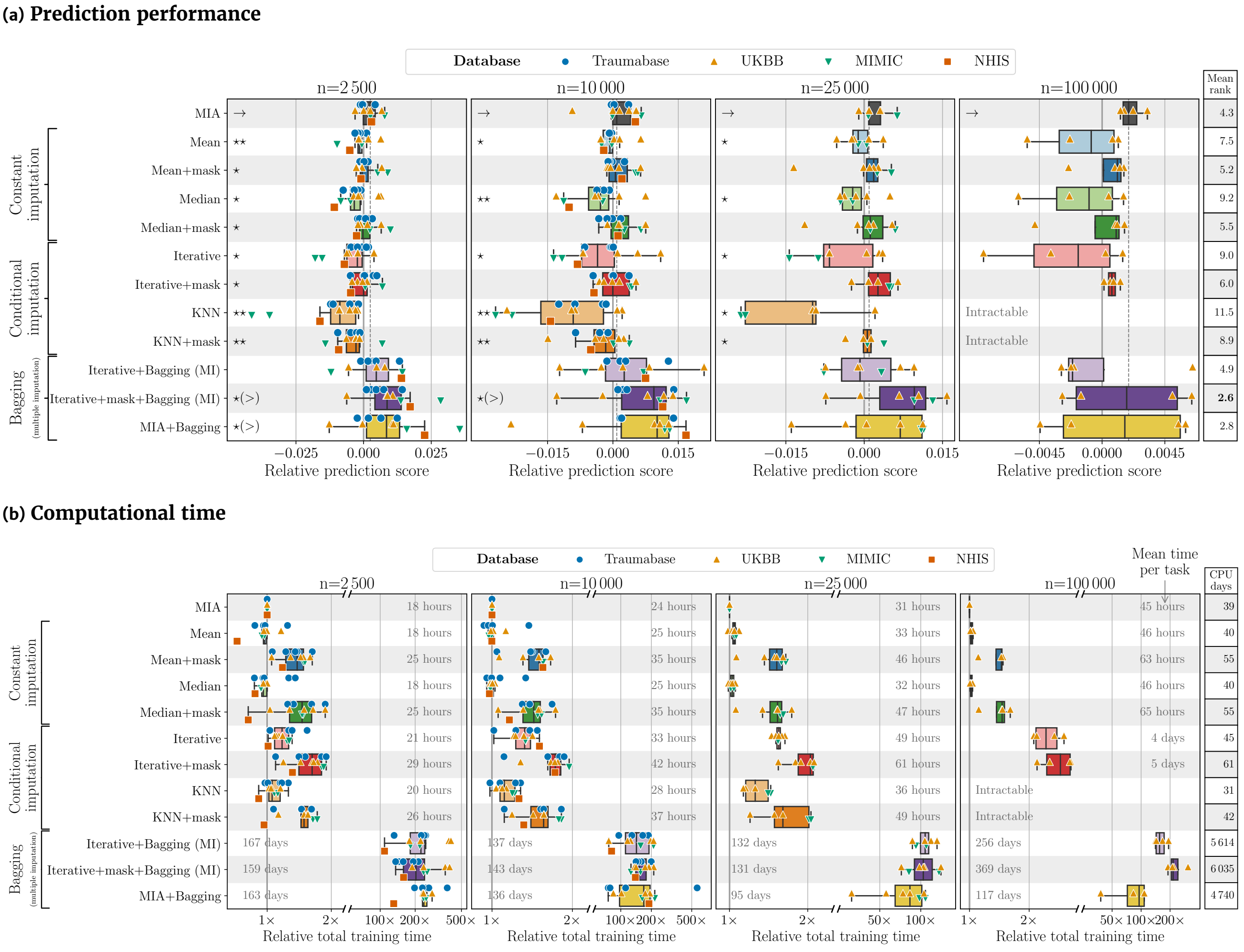

Figure 1. Gradient-boosted trees models. Comparison of prediction performance and training times across the 12 methods (see Table 1) for 13 prediction tasks spread over 4 databases, and for 4 sizes of dataset (2 500, 10 000, 25 000 and 100 000 samples). For each of the tasks and sizes, we computed a reference score by averaging the scores obtained by the 12 methods on the corresponding task and size. The relative prediction score of a method on a task and size is the deviation of the prediction score from the reference score of this task and size. For computational time, the total training time comprises imputation and tuning times and is given relative to the one of MIA for each task and size. More details on how these plots were created are given in the *Plotting method* section. The significance is assessed with a one-sided Wilcoxon signed-rank test with MIA taken as reference (see Supplementary Table 4a). Methods which performed significantly poorer (resp. better) at the 0.05 level are marked with "⋆" (resp. "⋆(>)") and "⋆⋆" (resp. "⋆ ⋆ (>)") for Bonferroni-corrected levels. Two tables give the overall average ranks and the total number of CPU days for each method, all tasks and sizes combined. The average number of CPU hours *per task* required to evaluate each method is given on each line. Detailed scores and ranks broken out by tasks are given in Supplementary Table 8 and Supplementary Figure 7. Notice that KNN and KNN+mask were intractable at $n = 100\,000$ due to their memory footprint of $\mathcal{O}(n^2)$.

one-sided Wilcoxon signed-rank test (Wilcoxon; 1945), all described in Demšar (2006).

The Friedman test compares the average ranks of several algorithms ran on several datasets. The null hypothesis assumes that all algorithms are equivalent, *i.e.* their rank should be equal. Table 3a shows that the null hypothesis is rejected with p-values way below the 0.05 level for the sizes 2 500, 10 000 and 25 000. This indicates that at least one algorithm has significantly different performances from one other on these sizes. Following Demšar (2006), we then proceed with a post-hoc analysis with the Nemenyi test, assessing the significance of the difference between two algorithms using a critical difference. Algorithms with a difference in ranks smaller than the critical difference are not significantly different. Unfortunately, there are many methods to compare (12) comparatively to the number of datasets (13). As a result, the critical difference is high as shown in equation (3) and Supplementary Table 3a and there is almost no significance when comparing the performance of MIA with the one of the other methods as shown on Supplementary Figure 3a. However, there are some significance when comparing bagged methods. For example at size n=2 500, Iterative+mask+Bagging and MIA+Bagging performed significantly better than Mean, Median, Iterative, KNN+mask and KNN.

We run a complementary analysis with a one-sided Wilcoxon signed-rank test, used for non-parametric tests comparing algorithms pairwise. We compare MIA to every other methods. The null hypothesis claims that the median of the score differences between the two methods is positive (resp. negative) for the one-sided right (resp. one-sided left) test. Results of the test is shown on Figure 1a and Supplementary Table 4b. At size n=2 500, MIA performed significantly better than every other non-bagged methods at the 0.05 level. MIA also performed significantly better than Mean, KNN and KNN+mask at the Bonferroni-corrected level. Bagged methods Iterative+mask+Bagging and MIA+Bagging performed significantly better than MIA at the 0.05 level. The bigger the size n, the less tasks are available and so the less significant are the results.

Adding the mask improves prediction

Imputations with the additional variable representing the mask perform systematically better in terms of average prediction score than their counterpart without mask (Figure 1a, Supplementary Table 8b).

In addition, MIA is not significantly better than the masked imputations yet it is for the non-masked imputations (Figure 1a, Supplementary Table 4). However, adding the mask leads to longer training times (Figure 1b). Indeed, adding the mask doubles the number of features for the supervised-learning step.

Conditional imputation is on par with constant imputation

Figure 1 shows that conditional imputation using Iterative or KNN imputers does not perform consistently better than constant imputation. The overall mean rank of Iterative and KNN are 9.0 and 11.5 versus 7.5 and 9.2 for Mean and Median respectively (Figure 1a and Supplementary Table 8b), and a similar delta is visible on the masked version.

Supplementary finding: Boosted-trees outperform linear methods

Imputation methods paired with a linear model performed poorer than when paired with boosted-trees (Supplementary Figure 2, Supplementary Table 9b). Additionally, boosted-trees paired with MIA are significantly better than every other method based on a linear model (Supplementary Table 4).

Discussion

Interpretation

Model aggregation drives the good performance of Multiple Imputation

As with standard multiple-imputation strategies used for parameter estimation, Bagging generates multiple bootstrap replicate training sets. Yet, the standard practice of Multiple Imputation strives to capture well the conditional distribution of the missing values given the observed one, while such conditional imputation is not needed for good prediction (as revealed by the good performance of MIA and Le Morvan et al.; 2021). Indeed, Bagging in itself is known to improve generalization. To answer whether the good performance of Multiple Imputation can be attributed to ensembling –averaging multiple predictors– or capturing the conditional distribution, we performed additional experiment with Mean+mask+Bagging (see Supplementary Figure 9). We observed that Mean+mask+Bagging is on par with Iterative+mask+Bagging, which suggests that the improved performances are rather due to the effect of Bagging itself rather than capturing the conditional distribution of the missing data given the observed ones.

Good imputation does not imply good prediction, even for Multiple Imputation

It may be surprising at first that a sophisticated conditional imputation does not outperform constant imputation. Indeed, it contradicts the intuition that better imputation should lead to better prediction. Theoretical work shows that this intuition is not always true (Le Morvan et al.; 2021): even in MAR settings, it may not hold for strongly non-linear mechanisms and little dependency across features. In the health databases that we studied, the features are weakly correlated: on average, only 12% of the features are correlated at more than 0.3 in absolute value (Supplementary Table 6). This low correlation among features may explain our findings. If features are mostly independent, there is little information on the unobserved values to be extracted from the observed ones. For supervised learning, constant imputation comes with the benefit that it creates a simple structure captured by the supervised-learning step, which can then adapt to the missingness (Josse et al.; 2019).

Boosted-trees with MIA give best predictive models at little cost

MIA, the missing-values support inside gradient-boosted trees, appears as a method of choice to deal with missing values. It was on average the best performing one in terms of performance in our extensive benchmark while having a low computational cost. Sophisticated conditional imputation such as the Iterative or KNN imputers are appealing because they may recover plausible values for missing entries, as discussed below. However, they are intractable with large datasets. Beyond the costs outlined by our experiments (Figure 1b), the broader problem is the algorithmic scalability: for a dataset of p features and n samples, the compute cost of a KNN imputer scales as n^2p^2 and the memory footprint as n^2, while the compute cost of an iterator imputer scales as $p^2n\min(n,p)$ when it is based on linear models, the cheapest alternative. If both p and n grow, these costs rapidly becomes prohibitive. They prevented us from exploring larger datasets, *e.g.* with more features. Note that to ground valid predictions, the imputation model must be learned only on the train set; hence it is recomputed many times in a cross-validation loop.

Regardless of missing-values handling, gradient-boosted trees predict significantly better than linear models (Supplementary Table 4b). Tree-based models excel on categorical or ordinal features, however these are only a minority of the features of the databases studied (Supplementary Figure 4). Hence the good performance of gradient-boosted trees probably reveals non-linear mechanisms in the data. Note that the smallest database that we explored has a sample size of n=2 500. For much smaller data, the simplest model –the linear model– may be the best choice.

The missingness is informative

For imputation-based pipelines, prediction significantly improves with the missingness mask added as input features. This suggests that the missingness is informative, which is often the case in health databases (Agniel et al.; 2018; Madden et al.; 2016). Hence for all health databases studied, either the covariates are Missing Not At Random (MNAR) or the outcome to predict depends on the missingness. Either cases fall outside of the theoretical framework that grounds the validity of statistical analysis using imputation (Rubin; 1976; Josse et al.; 2019). The empirical results also confirm that the practice of adding the mask as input allows to harness the predictive information in missing data patterns (Sperrin et al.; 2020), otherwise hidden in the imputed data and much more difficult to recover.

Features with high missing rates are also important.

Within each task, the missing rate per feature varies over a wide spectrum (see Supplementary Figure 6). We checked that features' missing rates and predictive importance were not associated. For this, we measured permutation features: the drop in a model score after shuffling a feature, thereby cancelling its contribution to the model performance. We ran this experiment for each task and each feature using scikit-learn's implementation (see Supplementary Table 5). We found no association between a feature's missing rate and its importance (Supplementary Figure 8). Predictions do not only rely on features with few missing values. Moreover, even features with a very high level of missing values (for example > 80%) seem to be as important as the others. This highlights the fact that it is worth making the effort of learning with incomplete features, even when they have a high missing rate.

Imputation may benefit robustness or interpretability

A good imputation may bring the benefit of recovering a meaningful missing value, reflecting a biological or clinical reality rather than operational constraints. For instance, the weight of a patient may be measured upon scheduled admission to a hospital but not at the emergency department. A predictive model based on an imputed underlying value may lend itself better to mechanistic interpretation than a model implicitly capturing missingness such as MIA. In addition, using missingness to drive prediction may be more fragile, *e.g.* to changes in the operational process. In such a case, shifts in the missing data patterns should be closely monitored (Sperrin et al.; 2020; van Smeden et al.; 2020; Groenwold; 2020) as they could seriously alter prediction performance. Indeed, machine-learning models building their predictions on “shortcuts” in the data –not directly related to outcome of interest but rather to the acquisition– sometimes generalize less well to new hospitals (DeGrave et al.; 2021). Nevertheless, in health the mere presence of a measure, such as a colonoscopy, is often an indication in itself.

Limitations and further work

Limitations: not all differences are significant

Relative performance of approaches varies across datasets, which is not surprising as no prediction model is expected to dominate on all data. The diversity of the datasets and the statistical analysis grounds the generality of the findings. Yet, not all differences are significant at large sample sizes. This lack of significance can simply be explained because of a small statistical power of the benchmark as only a few datasets are available to test these very large sample sizes settings (only 4 tasks at the n=100 000 size).

More datasets would probably have made more differences significant. Yet, the benchmarks presented here already incurred large computational costs, due to the nested cross-validation: about 520 000 CPU hours. Also, the findings build upon 13 different tasks, markedly more than the typical machine-learning benchmark: only 6% of empirical results published at NeurIPS and 8% ICLR (both leading machine-learning venues) build upon more than 10 datasets (Bouthillier and Varoquaux; 2020).

Limitations: imputation quality is not assessed

All the conclusions of this study pertain to prediction and do not allow us to conclude on imputation’s ability to accurately reconstruct missing values. The focus of our study is indeed on prediction.

Further work: more benchmarks would be interesting, and costly

To limit computation costs and mimic typical usage, no hyperparameters tuning was performed on the parameters of the imputers. Recently, software tools have been introduced to perform model selection on imputation jointly with the supervised step (Jarrett et al.; 2021; Borowski and Fic; 2021). Further evaluation could quantify how much gains are brought by such joint model selection, though it would need sizable computational resources.

Further work could test more supervised learning models. The motivation of the present study was not to find the absolute best pipeline, but rather to understand compromises that hold across datasets and are readily usable.

Conclusion

Extensive benchmarking on health databases reveals trends in the performance of methods to build predictive models handling missing values. First, directly incorporating missing values in tree-based models with MIA, gives a small but systematic improvement in prediction performance over prior imputation. Second, the computational cost of imputation using MICE or KNN becomes intractable for large datasets. Third, gradient-boosted trees give better predictions than linear models. Fourth, Bagging increases predictive performance but with a severe computational cost. Fifth, good imputation does not imply good prediction as both have different tradeoffs. Finally, the experiments reveal that the missingness is informative. Overall, a novel message of this benchmark is that for building predictive models, supervised learning directly handling missing values should be considered, beyond imputation.

Potential implications

This work suggests a departure from current practices: supervised learning directly handling missing values can be preferable to imputation. In particular, classic conditional expectation methods can be computationally intractable both in terms of time and memory on large datasets. Constant imputation with the mask also performs well with little costs.

Detailed benchmarking methodology

Experiment

We selected four real databases with missing values described in *Health databases*. From them we defined empirically 13 prediction tasks – that is a set of input features and an outcome to predict – with the intent of covering as diverse use cases as possible: regressions, classifications, diverse outcomes, diverse feature types (numerical, ordinal and categorical). We sub-sampled the datasets to study 4 sizes: 2 500, 10 000, 25 000 and 100 000 samples. We selected a subset of features from the databases for each prediction task using two approaches. Manually selecting or defining features based on articles or automatically selecting 100 encoded features using an univariate ANOVA selection. We often used the later because it has the advantage of not requiring expert knowledge to define the features. Manual selection keeps fewer features than our automated selection. Note that we one-hot encoded categorical features before selecting 100 encoded features with ANOVA. Less than 100 non-encoded features may thus be involved in the task. The ANOVA is fitted on one third of the samples and the two remaining thirds are kept for fitting and evaluating the methods. To reduce bias induced by the choice of subset on which is fit the ANOVA, we ran 5 trials in which the subset is each time redrawn and average the scores and times. Task having their features manually selected are given the whole samples and only 1 trial is performed. Each of the 12 methods is given the exact same features and cross-validation folds.
The next step consists in benchmarking the 12 methods of Table 1 on the defined prediction tasks. We used the implementation from scikit-learn (Pedregosa et al.; 2011) for all methods (see Supplementary Table 5). Two nested cross-validations are used. The outer one yields 5 training and test sets. On each training set, we perform a cross-validated hyper-parameter search –the inner cross-validation– and select the best hyper-parameters. We evaluate the best model on the respective test set. We assess the quality of the prediction with a coefficient of determination for regressions and the area under the ROC curve

for classification. We average the scores obtained on the 5 test sets of the outer cross-validation to give the final score. Finally, we compare averaged prediction scores one to each other.
We also monitored training and imputation times to add time concerns to our analysis. A very detailed description of the experimental method is available on protocols.io. A link to the protocol and to the code of the experiments is given in *Availability of supporting data and materials* and *Availability of source code and requirements*.

Plotting method

Figures on prediction scores

The experiment gives one prediction score per fold, per trial, per task, per method, per size. Cross-validations aggregate and average scores across the folds and trials resulting in an average score for each of the (task, method, size). For each one of the pairs (task, size), we computed a reference score by averaging the scores obtained by the 12 methods on the corresponding task and size. The plotted metric is what we called the relative prediction score - that is the deviation of the prediction score from the reference score - for each of the (task, method, size). We created one box plot for each of the 4 sizes with the same structure: the relative prediction score on the x-axis and the 12 methods on the y-axis. Each is overlaid with a scatter plot plotting the relative prediction score per (task, method, size). The scatter plot shares its x-axis with the box plot. On the y-axis however, each dot is given a y coordinate according to its method and database so that scores coming from a same method and database are plotted on the same horizontal line.

Figures on computation time

Computational time plots follow the same structure. The metric of interest is now the total training time. It includes imputation time and the full hyper-parameters tuning time. It is evaluated using computer's process time instead of wall-clock time. The total training time of MIA is taken as reference time for each (task, size). The relative total training time is computed by dividing by the reference time. The x-scale is logarithmic to better apprehend comparison on large scales.

Availability of source code and requirements

- Project name: Benchmarking missing-values approaches for predictive models on health databases.
- Project home page: https://github.com/alexprz/article-benchmark_mv_approaches
- Operating system: Platform independent
- Programming language: Python 3.7.6
- Other requirements: all requirements are listed in the `requirements.txt` file of the repository.
- License: MIT

Availability of supporting data and materials

The datasets supporting the results of this article are available at the following URL.

- **Traumabase**, by contacting the team at http://www.traumabase.eu/en_US/contact.
- **UKBB**: upon application at https://www.ukbiobank.ac.uk/register-apply/.
- **MIMIC-III**: upon application at https://mimic.physionet.org/gettingstarted/access/.
- **NHIS** freely available at https://www.cdc.gov/nchs/nhis/nhis_2017_data_release.htm.

A thorough description of the protocols of the experiments conducted in this article is available on protocols.io at: https://www.protocols.io/view/benchmarking-missing-values-approaches-for-predict-bmvgk63w.

Declarations

List of abbreviations

The following abbreviations were used in this article:

- ANOVA: Analysis Of Variance.
- AUC: Area Under the Curve.
- KNN: K-Nearest Neighbors.
- MAR: Missing At Random
- MIA: Missing Incorporated in Attribute.
- MNAR: Missing Not At Random
- MIMIC: Medical Information Mart for Intensive Care.
- NHIS: National Health Interview Survey.
- LOWESS : Locally Weighted Scatterplot Smoothing.
- UKBB: UK Biobank.

Ethical Approval

Only secondary data use was involved in this work. Each one of the databases has previously been approved by the Ethical Review Board. Access to each database was obtained according to the corresponding rules and authorization, when applicable (MIMIC, Traumabase, UKBB).

Consent for publication

Not applicable.

Competing Interests

The authors declare that they have no competing interests.

Funding

Gaël Varoquaux, Julie Josse, and Marine Le Morvan acknowledge funding from DataAI under the MissingBigData grant. Gaël Varoquaux and Marine Le Morvan acknowledge funding from ANR under the DirtyData grant (ANR-17-CE23-0018). Alexandre Perez-Lebel was partially funded by a Mitacs Globalink Research Award for research in Canada. Jean-Baptiste Poline is partially funded by the National Institutes of Health (NIH) NIH-NIBIB P41 EB019936 (ReproNim) NIH-NIMH R01 MH083320 (CANDIShare) and NIH RF1 MH120021 (NIDM), the National Institute Of Mental Health of the NIH under Award Number R01MH096906 (Neurosynth), as well as the Canada First Research Excellence Fund, awarded to McGill University for the Healthy Brains for Healthy Lives initiative and the Brain Canada Foundation with support from Health Canada.

Author's Contributions

Following the CRediT Contributor Roles Taxonomy:

- Alexandre Perez-Lebel: Investigation, Data curation, Formal Analysis, Writing - original draft, Writing - review & editing.

- Gaël Varoquaux: Funding acquisition, Conceptualization, Supervision, Writing - review & editing, Validation.
- Marine Le Morvan: Supervision, Writing - review & editing, Validation.
- Julie Josse: Writing - review & editing.
- Jean-Baptiste Poline: Funding acquisition, Supervision, Writing - review & editing, Validation.

Acknowledgements

Not applicable.

Authors' information

Alexandre Perez-Lebel is an engineering student specialized in applied mathematics, computer science and machine learning. He interned one year at the Montreal Neurological Institute of McGill University in Montreal and is currently pursuing a PhD program in machine learning at Inria.

References

Agniel, D., Kohane, I. S. and Weber, G. M. (2018). Biases in electronic health record data due to processes within the healthcare system: retrospective observational study, *Bmj* **361**.

Bertsimas, D., Pawlowski, C. and Zhuo, Y. D. (2018). From Predictive Methods to Missing Data Imputation: An Optimization Approach, *Journal of Machine Learning Research* **18**(196): 1–39.
URL: *http://jmlr.org/papers/v18/17-073.html*

Borowski, J. and Fic, P. (2021). NADIA r package, https://cran.r-project.org/web/packages/NADIA/index.html. Accessed: 2021-05-26.

Bouthillier, X. and Varoquaux, G. (2020). Survey of machine-learning experimental methods at NeurIPS2019 and ICLR2020, *Research report*, Inria Saclay Ile de France.
URL: *https://hal.archives-ouvertes.fr/hal-02447823*

Buuren, S. v. and Groothuis-Oudshoorn, K. (2010). mice: Multivariate imputation by chained equations in r, *Journal of statistical software* pp. 1–68.

Chen, J. and Shao, J. (2000). Nearest neighbor imputation for survey data, *Journal of official statistics* **16**(2): 113.

Chen, T. and Guestrin, C. (2016). Xgboost: A scalable tree boosting system, *Proceedings of the 22nd acm sigkdd international conference on knowledge discovery and data mining*, pp. 785–794.

DeGrave, A. J., Janizek, J. D. and Lee, S.-I. (2021). Ai for radiographic covid-19 detection selects shortcuts over signal, *Nature Machine Intelligence* pp. 1–10.

Dempster, A. P., Laird, N. M. and Rubin, D. B. (1977). Maximum Likelihood from Incomplete Data via the EM Algorithm, *Journal of the Royal Statistical Society. Series B (Methodological)* **39**(1): 1–38.
URL: *http://www.jstor.org/stable/2984875*

Demšar, J. (2006). Statistical Comparisons of Classifiers over Multiple Data Sets, *Technical Report 1*.
URL: *http://jmlr.org/papers/v7/demsar06a.html*

Friedman, J., Hastie, T. and Tibshirani, R. (2001). *The elements of statistical learning*, Vol. 1, Springer series in statistics New York.

Friedman, M. (1937). The Use of Ranks to Avoid the Assumption of Normality Implicit in the Analysis of Variance, *Journal of the American Statistical Association* **32**(200): 675–701.
URL: *https://www.tandfonline.com/doi/abs/10.1080/01621459.1937.10503522*

Friedman, M. (1940). A Comparison of Alternative Tests of Significance for the Problem of *m* Rankings, *The Annals of Mathematical Statistics* **11**(1): 86–92.
URL: *https://doi.org/10.1214/aoms/1177731944*

Groenwold, R. H. (2020). Informative missingness in electronic health record systems: the curse of knowing, *Diagnostic and prognostic research* **4**(1): 1–6.

Guyon, I. and Elisseeff, A. (2003). An introduction to variable and feature selection, *Journal of machine learning research* **3**: 1157.

Iman, R. L. and Davenport, J. M. (1980). Approximations of the critical region of the fbietkan statistic, *Communications in Statistics - Theory and Methods* **9**(6): 571–595.
URL: *https://doi.org/10.1080/03610928008827904*

Jäger, S., Allhorn, A. and Bießmann, F. (2021). A Benchmark for Data Imputation Methods, *Frontiers in Big Data* **0**: 48.

Jarrett, D., Yoon, J., Bica, I., Qian, Z., Ercole, A. and van der Schaar, M. (2021). Clairvoyance: A pipeline toolkit for medical time series, *International Conference on Learning Representations*.

Jiang, W., Josse, J. and Lavielle, M. (2020). Logistic regression with missing covariates—Parameter estimation, model selection and prediction within a joint-modeling framework, *Computational Statistics and Data Analysis* **145**: 106907.

Johnson, A. E., Pollard, T. J., Shen, L., Lehman, L. W. H., Feng, M., Ghassemi, M., Moody, B., Szolovits, P., Anthony Celi, L. and Mark, R. G. (2016). MIMIC-III, a freely accessible critical care database, *Scientific Data* **3**(1): 1–9.
URL: *www.nature.com/sdata/*

Josse, J., Prost, N., Scornet, E. and Varoquaux, G. (2019). On the consistency of supervised learning with missing values, *arXiv preprint arXiv:1902.06931* .

Khan, S. S., Ahmad, A. and Mihailidis, A. (2019). Bootstrapping and multiple imputation ensemble approaches for missing data.

Läll, K., Lepamets, M., Palover, M., Esko, T., Metspalu, A., Tõnisson, N., Padrik, P., Mägi, R. and Fischer, K. (2019). Polygenic prediction of breast cancer: Comparison of genetic predictors and implications for risk stratification, *BMC Cancer* **19**(1).
URL: *https://pubmed.ncbi.nlm.nih.gov/31182048/*

Le Morvan, M., Josse, J., Scornet, E. and Varoquaux, G. (2021). What's a good imputation to predict with missing values?, *NeurIPS* .

Le Morvan, M., Prost, N., Josse, J., Scornet, E. and Varoquaux, G. (2020). Linear predictor on linearly-generated data with missing values: non consistency and solutions, *in* S. Chiappa and R. Calandra (eds), *Proceedings of the Twenty Third International Conference on Artificial Intelligence and Statistics*, Vol. 108 of *Proceedings of Machine Learning Research*, PMLR, pp. 3165–3174.
URL: *http://proceedings.mlr.press/v108/morvan20a.html*

Little, R. J. and Rubin, D. B. (2019). *Statistical analysis with missing data*, Vol. 793, John Wiley & Sons.

Madden, J. M., Lakoma, M. D., Rusinak, D., Lu, C. Y. and Soumerai, S. B. (2016). Missing clinical and behavioral health data in a large electronic health record (ehr) system, *Journal of the American Medical Informatics Association* **23**(6): 1143–1149.

Miotto, R., Li, L., Kidd, B. A. and Dudley, J. T. (2016). Deep patient: an unsupervised representation to predict the future of patients from the electronic health records, *Scientific reports* **6**(1): 1–10.

National Center for Health Statistics (2017). National Health Interview Survey (NHIS).
URL: *https://www.cdc.gov/nchs/nhis/nhis_2017_data_*

release.htm
Nemenyi, P. (1963). *Distribution-free Multiple Comparisons*, Princeton University.
URL: https://books.google.fr/books?id=nhDMtgAACAAJ
Olson, R. S., Cava, W. L., Mustahsan, Z., Varik, A. and Moore, J. H. (2018). Data-driven advice for applying machine learning to bioinformatics problems, *PACIFIC SYMPOSIUM ON BIOCOMPUTING 2018: Proceedings of the Pacific Symposium*, World Scientific, pp. 192–203.
Pedregosa, F., Varoquaux, G., Gramfort, A., Michel, V., Thirion, B., Grisel, O., Blondel, M., Prettenhofer, P., Weiss, R., Dubourg, V. et al. (2011). Scikit-learn: Machine learning in python, *the Journal of machine Learning research* **12**: 2825.
Poulos, J. and Valle, R. (2018). Missing Data Imputation for Supervised Learning, *Applied Artificial Intelligence* **32**(2): 186–196.
URL: *https://www.tandfonline.com/doi/abs/10.1080/08839514.2018.1448143*
Rose, S. and Rizopoulos, D. (2020). Machine learning for causal inference in biostatistics, *Biostatistics* **21**(2): 336–338.
Rubin, D. B. (1976). Inference and missing data, *Biometrika* **63**(3): 581–592.
URL: https://academic.oup.com/biomet/article/63/3/581/270932
Sharafoddini, A., Dubin, J. A., Maslove, D. M. and Lee, J. (2019). A New Insight Into Missing Data in Intensive Care Unit Patient Profiles: Observational Study., *JMIR medical informatics* **7**(1): e11605.
Shwartz-Ziv, R. and Armon, A. (2021). Tabular data: Deep learning is not all you need, *arXiv preprint arXiv:2106.03253* .
Sperrin, M., Martin, G. P., Sisk, R. and Peek, N. (2020). Missing data should be handled differently for prediction than for description or causal explanation.
Steele, A. J., Denaxas, S. C., Shah, A. D., Hemingway, H. and Luscombe, N. M. (2018). Machine learning models in electronic health records can outperform conventional survival models for predicting patient mortality in coronary artery disease, *PloS one* **13**(8): e0202344.
Sudlow, C., Gallacher, J., Allen, N., Beral, V., Burton, P., Danesh, J., Downey, P., Elliott, P., Green, J., Landray, M., Liu, B., Matthews, P., Ong, G., Pell, J., Silman, A., Young, A., Sprosen, T., Peakman, T. and Collins, R. (2015). UK Biobank: An Open Access Resource for Identifying the Causes of a Wide Range of Complex Diseases of Middle and Old Age, *PLoS Medicine* **12**(3).
URL: https://pubmed.ncbi.nlm.nih.gov/25826379/
Twala, B. E. T. H., Jones, M. C. and Hand, D. J. (2008). Good methods for coping with missing data in decision trees, *Pattern Recogn. Lett.* **29**: 950–956.
Van Buuren, S. (2018). *Flexible imputation of missing data*, CRC press.
van Smeden, M., Groenwold, R. H. and Moons, K. G. (2020). A cautionary note on the use of the missing indicator method for handling missing data in prediction research, *Journal of clinical epidemiology* **125**: 188–190.
Wells, B. J., Chagin, K. M., Nowacki, A. S. and Kattan, M. W. (2013). Strategies for handling missing data in electronic health record derived data, *Egems* **1**(3).
Wilcoxon, F. (1945). Individual Comparisons by Ranking Methods, *Biometrics Bulletin* **1**(6): 80–83.
URL: http://www.jstor.org/stable/3001968
Zheng, T., Xie, W., Xu, L., He, X., Zhang, Y., You, M., Yang, G. and Chen, Y. (2017). A machine learning-based framework to identify type 2 diabetes through electronic health records, *International journal of medical informatics* **97**: 120–127.

(a) Prediction performance

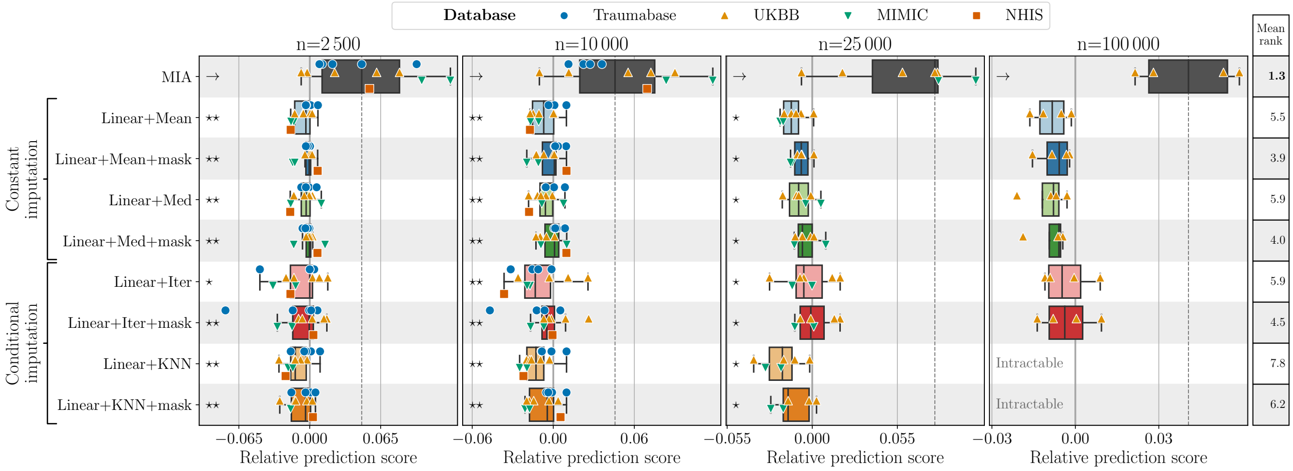

(b) Computational time

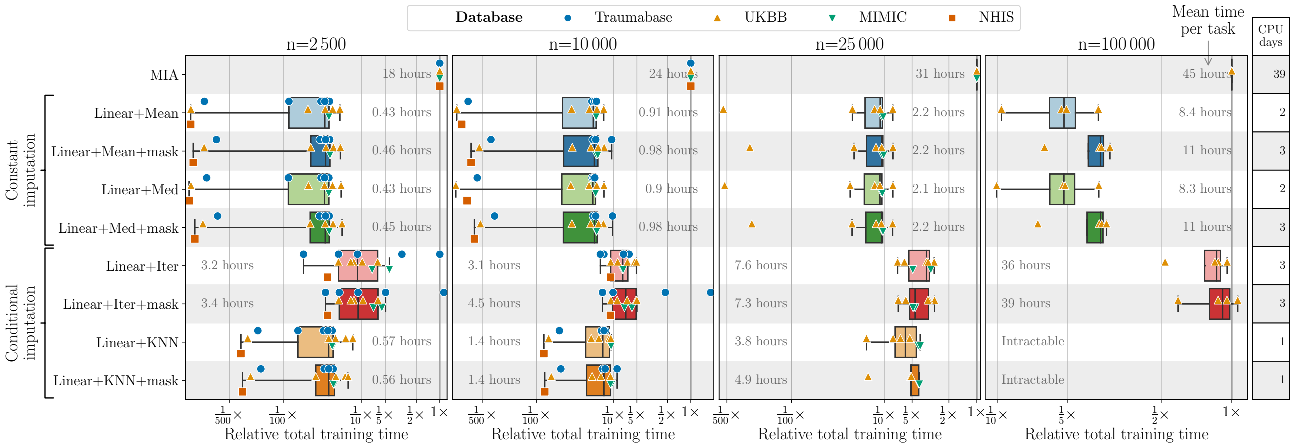

Figure 2. Supplementary results: linear and gradient-boosted trees models. Comparison of prediction performance and training times across the 9 methods (linear models and gradient boosting trees, see Supplementary Table 2) for 13 prediction tasks spread over 4 databases, and for 4 sizes of dataset (2 500, 10 000, 25 000 and 100 000 samples). For linear models, ridge is used for regressions and logistic regression for classifications. For each of the tasks and sizes, we computed a reference score by averaging the scores obtained by the 9 methods on the corresponding task and size. The relative prediction score of a method on a task and size is the deviation of the prediction score from the reference score of this task and size. For computational time, the total training time comprises imputation and tuning times and is given relative to the one of MIA for each task and size. More details on how these plots were created are given in the *Detailed benchmarking methodology* section. The significance is assessed with a one-sided Wilcoxon signed-rank test with MIA taken as reference (see Supplementary Table 4b). Methods which performed significantly poorer (resp. better) at the 0.05 level are marked with "⋆" (resp. "⋆(>)") and "⋆⋆" (resp. "⋆⋆(>)") for Bonferroni-corrected levels. Two tables give the overall average ranks and the total number of CPU days for each method, all tasks and sizes combined. The average number of CPU hours *per task* required to evaluate each method is given on each line. Detailed scores and ranks broken out by tasks are given in Supplementary Table 9. Notice that KNN and KNN+mask were intractable at n=100 000 due to their memory footprint of $\mathcal{O}(n^2)$.

Appendix

Supplementary experiment: linear models or trees?

Protocol

This supplementary experiment uses the same pipeline as the main experiment except that imputation is paired with linear models instead of boosted trees as summarized in Supplementary Table 2. We used ridge for regressions, and ℓ_2-penalized logistic regression for classifications.

Findings: trees with MIA improve upon linear models

MIA with boosted trees outperforms all 8 combinations of imputers with linear models, on every size and every database. Supplementary Figure 2a shows that MIA obtained the best average rank of 1.3 far ahead of other methods. The following ones are Linear+Mean+mask, Linear+Med+mask and Linear+Iter+mask with a rank of 3.9, 4.0 and 4.5 respectively. The one-sided Wilcoxon signed-rank test confirms this claim. Supplementary Table 4 shows that MIA with boosted trees is significantly better than every linear methods on the first two sizes even at the Bonferroni-corrected level. The null hypothesis of the Friedman test is rejected below the 0.05 level except for the last size as shown on Supplementary Table 3b. Thus methods are not equivalent for the first three sizes. The Nemenyi test on Supplementary Figure 3b confirms that results are not significant for the larger size.

Moreover, we were expecting the mean and median imputations to give bad results being paired with linear models as shown in Le Morvan et al. (2020). Not only these results confirm our expectations, but they also show that non-constant imputation models give similar results when paired with a linear model. As before, masked versions perform slightly better than their no-mask counterpart.

However, gradient-boosted trees with MIA are a lot slower than imputation with linear models. Supplementary Figure 2b shows that boosted trees with MIA is up to 500 times slower than constant imputations with linear models. Also, condi-

Table 2. Methods compared in the supplementary experiment.

In-article name	Imputer	Mask	Predictive model
Boosted trees+MIA	-	-	Boosted trees
Linear+Mean	Mean	No	Ridge/Logit
Linear+Mean+mask	Mean	Yes	Ridge/Logit
Linear+Med	Median	No	Ridge/Logit
Linear+Med+mask	Median	Yes	Ridge/Logit
Linear+Iter	Iterative	No	Ridge/Logit
Linear+Iter+mask	Iterative	Yes	Ridge/Logit
Linear+KNN	KNN	No	Ridge/Logit
Linear+KNN+mask	KNN	Yes	Ridge/Logit

tional imputation leads to slower computations than mean and median imputation. Given the low gain obtained against mean and median imputation, they are of limited interest.
The main takeaway is the outperformance in score of MIA with gradient-boosted trees over imputation with linear models when it comes to handling missing values. This outperformance comes with a cost: a much longer computation time.

Significance tests

In the following paragraphs, we took the notations and formulations of Demšar (2006). We consider k algorithms and N datasets. We note r_i^j the rank of the j-th algorithm on the i-th dataset. Note $R_j = \frac{1}{N}\sum_i r_i^j$ the average rank.

Friedman test. The Friedman statistic χ_F^2 is distributed according to a chi-square distribution with $k-1$ degrees of freedom.

$$\chi_F^2 = \frac{12N}{k(k+1)}\left[\sum_j R_j^2 - \frac{k(k+1)^2}{4}\right] \quad (1)$$

Iman and Davenport (1980) derived a less conservative statistic F_F which is distributed according to the F-distribution with $k-1$ and $(k-1)(N-1)$ degrees of freedom.

$$F_F = \frac{(N-1)\chi_F^2}{N(k-1)-\chi_F^2} \quad (2)$$

Both statistics (1) and (2) are given on Supplementary Table 3 with their associated p-values for the 2 sets of methods and the 4 sizes of dataset.

Table 3. Friedman test, correction by Iman and Davenport and Nemenyi test. CD is the critical distance and N the number of tasks for each size.

(a) Tree-based methods of Table 1.

Size	χ_F^2	χ_F^2 p-value	F_F	F_F p-value	CD	N
2500	73	9.6e-11	12	7.4e-16	4.6	13
10000	76	2.3e-11	15	6.1e-18	4.8	12
25000	30	2.5e-03	3.9	2.4e-04	6.3	7
100000	10	0.43	1.2	0.35	6.8	4

(b) Boosted-trees and linear methods of Supplementary Table 2.

Size	χ_F^2	χ_F^2 p-value	F_F	F_F p-value	CD	N
2500	41	5.1e-06	7.8	5.2e-08	3.3	13
10000	50	9.7e-08	12	1.7e-11	3.5	12
25000	23	5.6e-03	4.3	6.2e-04	4.5	7
100000	-19	1	-1.1	1	6	4

Figure 3. Mean ranks by method and by size of dataset. The critical difference is computed using the Nemenyi test (equation (3) and Supplementary Table 3). Methods within the critical difference range do not perform significantly differently from one another according to the Nemenyi test. Methods within the critical difference range of MIA are in red, others in black.

(a) Tree-based methods.

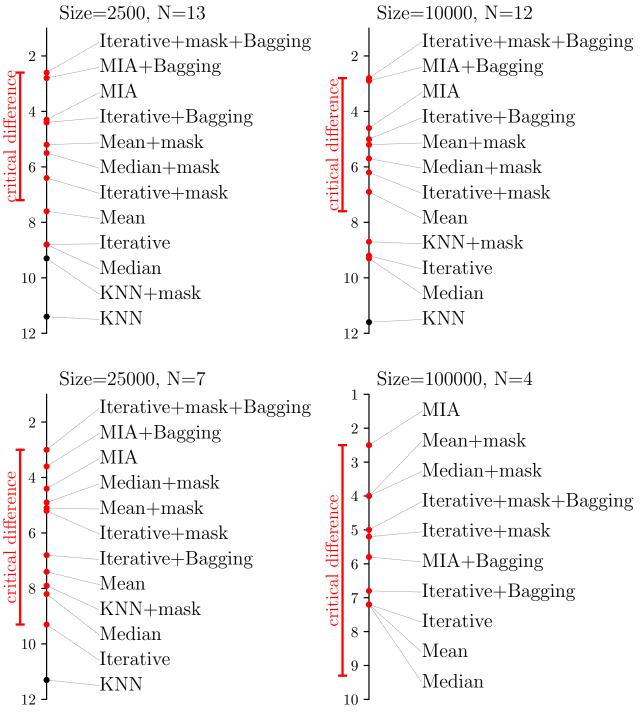

(b) Boosted trees+MIA vs linear methods.

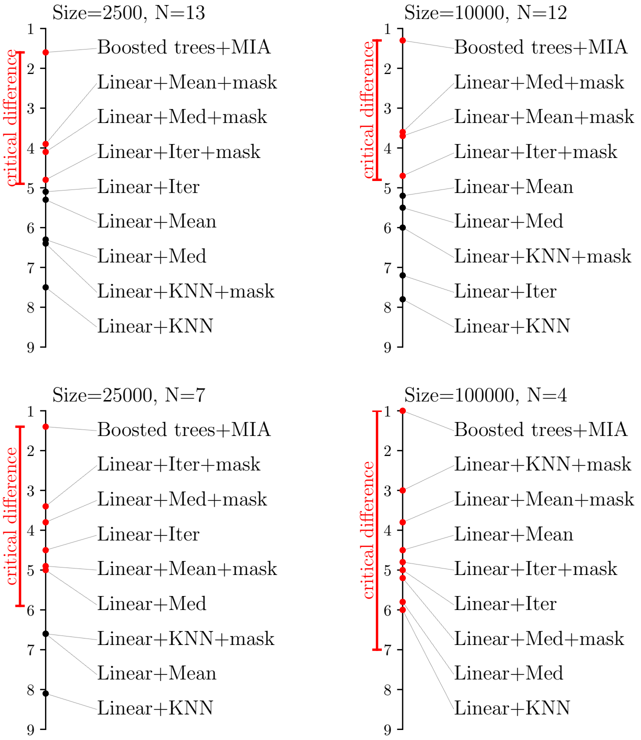

Nemenyi test. Once the Friedman test is rejected, the Nemenyi test can be applied. It provides a critical difference CD which is the minimal difference between the average ranks of two algorithms for them to be significantly different.

$$CD = q_\alpha \sqrt{\frac{k(k+1)}{6N}} \quad (3)$$

Values of q_α are given in Table 5 of Demšar (2006). Values of critical differences for the 2 sets of methods and the 4 sizes of dataset are given in Supplementary Table 3.

Wilcoxon signed-rank test. To compute the one-sided Wilcoxon signed-rank test, we used the `wilcoxon` function of the `scipy.stats` module between the 13 average scores of MIA against the ones of every other methods. Resulting p-values are given in Supplementary Table 4 for the 4 sizes of dataset.

Table 4. One-sided Wilcoxon signed-rank test. p-values of the one-sided right test on the difference of score between MIA and every other method for Table 4a, and between gradient-boosted trees and linear models for Table 4b. p-values below the 0.05 level are marked with *. p-values below the Bonferroni corrected level are marked with **. When the reversed test (*i.e.* one-sided left) is significant instead, p-values are marked with $^{*(>)}$ and $^{**(>)}$ following the same rule.

(a) MIA vs imputation. Bonferroni level: $0.05/19 = 2.6 \times 10^{-3}$. Rejecting the null hypothesis means MIA performed better than the compared method.

Size<br>Method	2500	10000	25000	100000
Mean	1.2e-03**	4.6e-02*	2.3e-02*	6.2e-02
Mean+mask	4.0e-02*	2.3e-01	1.5e-01	6.2e-02
Median	5.2e-03*	1.7e-03**	2.3e-02*	6.2e-02
Median+mask	4.0e-02*	2.1e-01	1.5e-01	1.2e-01
Iterative	5.2e-03*	3.2e-02*	3.9e-02*	6.2e-02
Iterative+mask	2.4e-02*	2.1e-01	4.7e-01	6.2e-02
KNN	1.2e-04**	2.4e-04**	3.1e-02*	
KNN+mask	1.2e-04**	7.3e-04**	3.1e-02*	
MI	8.1e-01	6.6e-01	3.4e-01	1.2e-01
MI+mask	9.9e-01*(>)	9.6e-01*(>)	9.2e-01	4.4e-01
MIA+bagging	9.7e-01*(>)	9.4e-01	7.7e-01	3.1e-01
Linear+Mean	6.1e-04**	4.9e-04**	7.8e-03*	6.2e-02
Linear+Mean+mask	8.5e-04**	7.3e-04**	1.6e-02*	6.2e-02
Linear+Med	6.1e-04**	4.9e-04**	7.8e-03*	6.2e-02
Linear+Med+mask	6.1e-04**	4.9e-04**	1.6e-02*	6.2e-02
Linear+Iter	3.1e-03*	1.2e-03**	1.6e-02*	6.2e-02
Linear+Iter+mask	2.3e-03**	1.2e-03**	1.6e-02*	6.2e-02
Linear+KNN	1.2e-04**	2.4e-04**	1.6e-02*	5.0e-01
Linear+KNN+mask	1.2e-04**	2.4e-04**	3.1e-02*	5.0e-01

(b) Gradient-boosted trees vs linear models. Bonferroni level: $0.05/8 = 6.25 \times 10^{-3}$. Rejecting the null hypothesis means gradient-boosted trees performed better than linear models for the given imputer.

Size<br>Imputer	2500	10000	25000	100000
Mean	1.2e-03**	4.9e-04**	1.6e-02*	6.2e-02
Mean+mask	1.7e-03**	4.9e-04**	1.6e-02*	6.2e-02
Median	6.1e-04**	7.3e-04**	1.6e-02*	6.2e-02
Median+mask	8.5e-04**	2.4e-04**	1.6e-02*	6.2e-02
Iterative	4.0e-03**	1.2e-03**	2.3e-02*	6.2e-02
Iterative+mask	2.3e-03**	1.2e-03**	1.6e-02*	6.2e-02
KNN	8.5e-04**	7.3e-04**	3.1e-02*	
KNN+mask	8.5e-04**	4.9e-04**	3.1e-02*	

Table 5. Scikit-learn's implementations of the methods.

In-article name	Scikit-learn's method
Boosted trees	`HistGradientBoostingRegressor`, `HistGradientBoostingClassifier`
Linear model	`Ridge`, `LogisticRegression`
Mean, Mean+mask	`SimpleImputer`
Median, Median+mask	`SimpleImputer`
Iterative, Iterative+mask	`IterativeImputer`
KNN, KNN+mask	`KNNImputer`
ANOVA selection	`f_regression`, `f_classif`
Permutation importance	`permutation_importance`
Bagging	`BaggingRegressor`, `BaggingClassifier`

Table 6. Correlation between features. Average number of ordinal and numerical features correlated to other ordinal or numerical features with an absolute correlation coefficient larger than thresholds $\{0.1, 0.2, 0.3\}$, averaged on all ordinal and numerical features of the task and expressed in percentage of the number of ordinal and numerical features in the task. For example in the task "death_screening", a numerical or ordinal feature has an absolute correlation value greater than 0.01 with 68% of the ordinal and numerical features of the task in average.

			Threshold		
Database	Task	# features	0.1	0.2	0.3
Traumabase	death_screening	92	68%	41%	22%
	hemo	12	50%	23%	12%
	hemo_screening	76	65%	36%	20%
	platelet_screening	90	67%	40%	22%
	septic_screening	76	68%	37%	18%
UKBB	breast_25	11	40%	20%	19%
	breast_screening	100	26%	12%	8%
	fluid_screening	100	21%	10%	6%
	parkinson_screening	100	28%	16%	11%
	skin_screening	100	24%	11%	8%
MIMIC	hemo_screening	100	22%	6%	3%
	septic_screening	100	21%	6%	2%
NHIS	income_screening	78	15%	6%	4%
Average		79	40%	20%	12%

Figure 4. Types of features. Number of categorical, ordinal and numerical features in each dataset, *before encoding*. Note that one non-encoded categorical feature can lead to several selected encoded features. Since we select 100 encoded features, some task have less than 100 non-encoded features. For tasks having several trials, five horizontal bars are plotted representing one trial each, as feature selection may select different features.

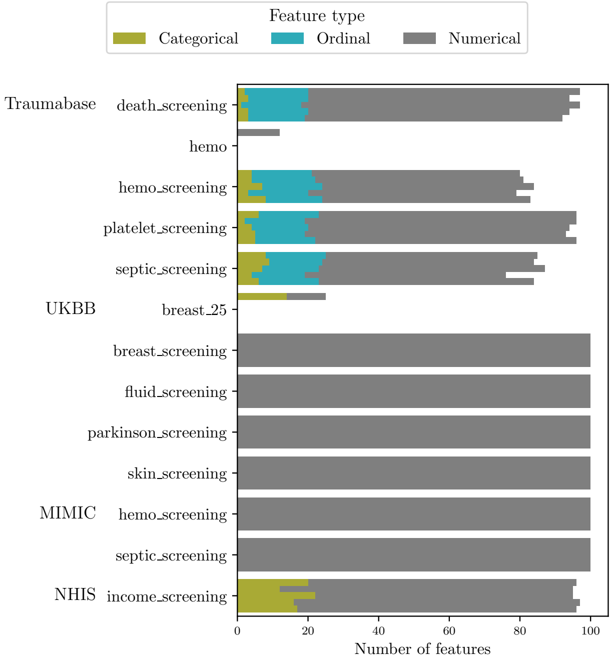

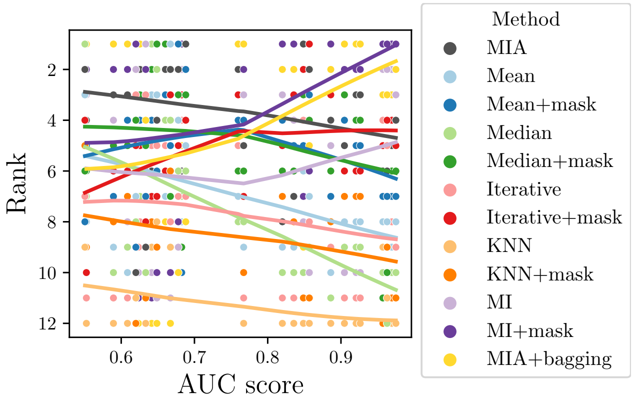

(a) Classification tasks

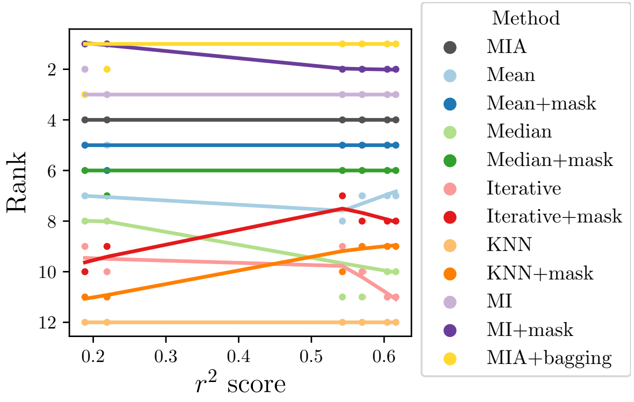

(b) Regression tasks

Figure 5. Effect of difficulty on the ranks of the methods. For each task and size, the average score obtained by the methods is taken as a proxy of its difficulty. Local regressions (LOWESS) are plotted for each method to better visualize trends.

Effect of tasks' difficulty on the performance of the methods. For classification tasks, Supplementary Figure 5a shows the relative performance of the methods as a function of the tasks' difficulty. Bagged methods Iterative+mask+Bagging and MIA+Bagging show a clear trend with lower (resp. higher) ranks for easier (resp. harder) methods. Also, MIA is the best performing one for harder tasks (for AUC < 0.8). Thus, the interest of MIA seems more pronounced for harder tasks. There is not enough regression tasks to observe exploitable trends on Supplementary Figure 5b.

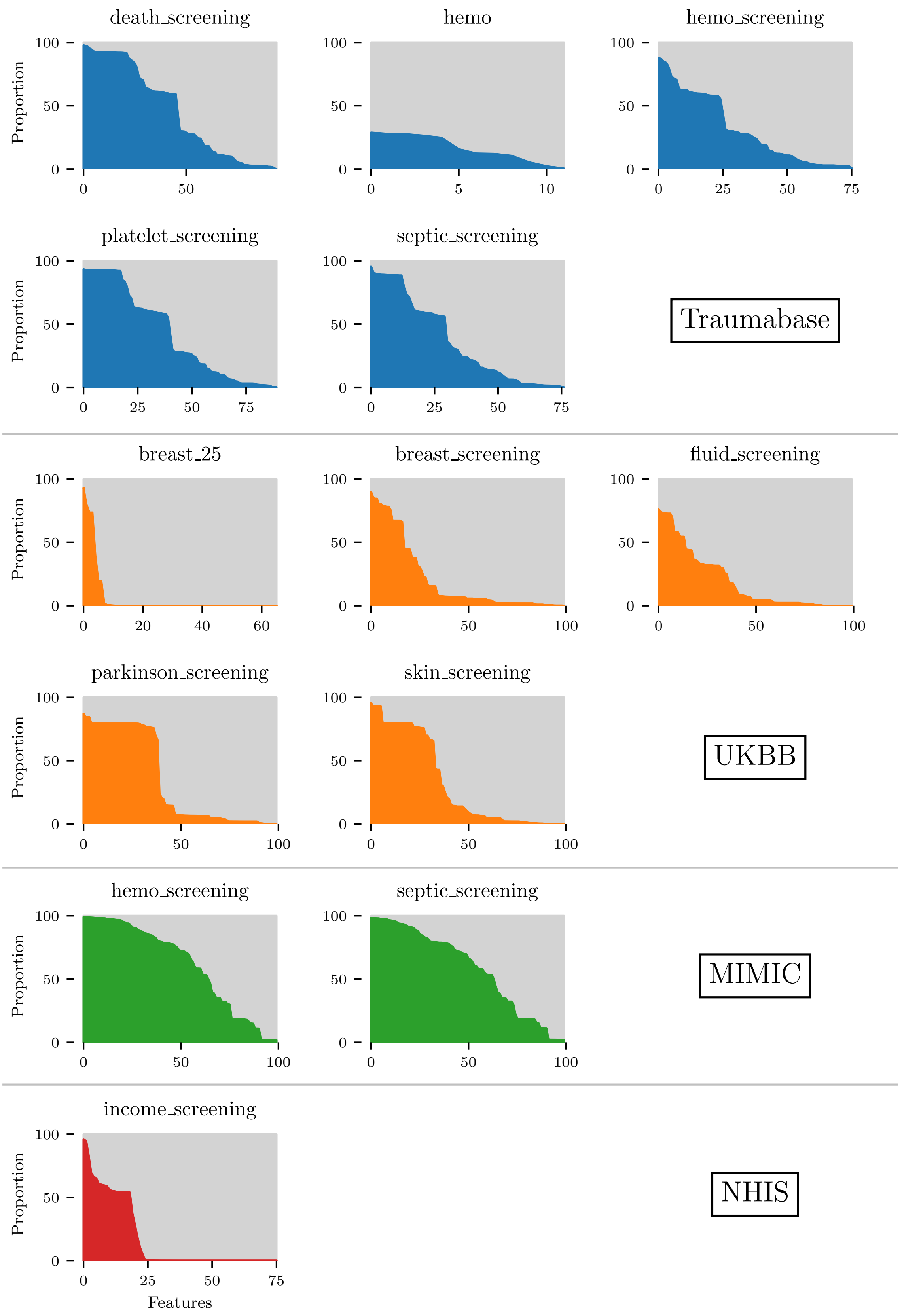

Figure 6. Missing values distribution. Proportion of missing values across selected encoded features for each task and for trial number 1, sorted in decreasing order. Other trials have similar proportions.

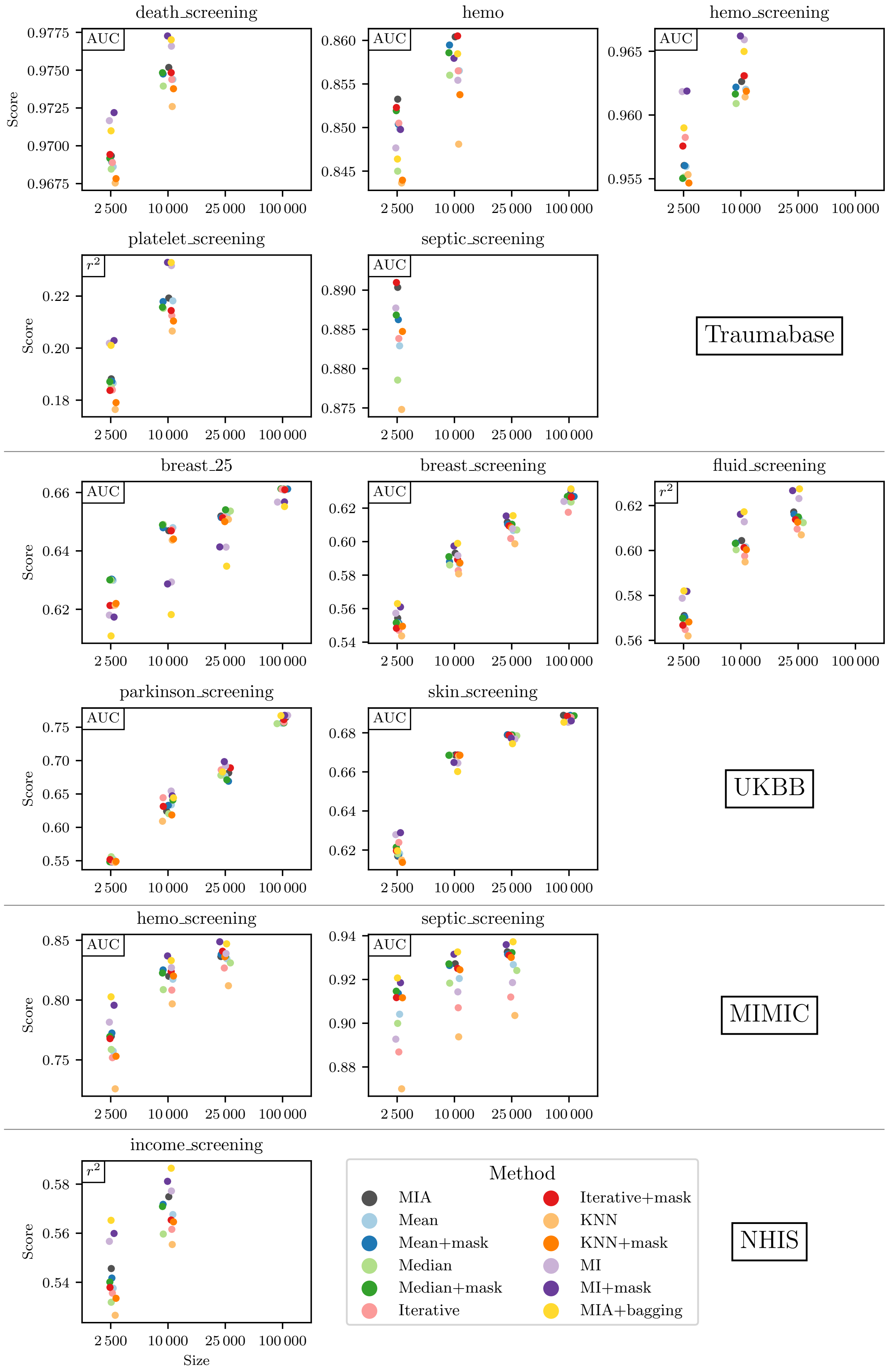

Figure 7. Scores of the tree-based methods as a function of the training size. Detailed scores of Figure 1 broken out by task.

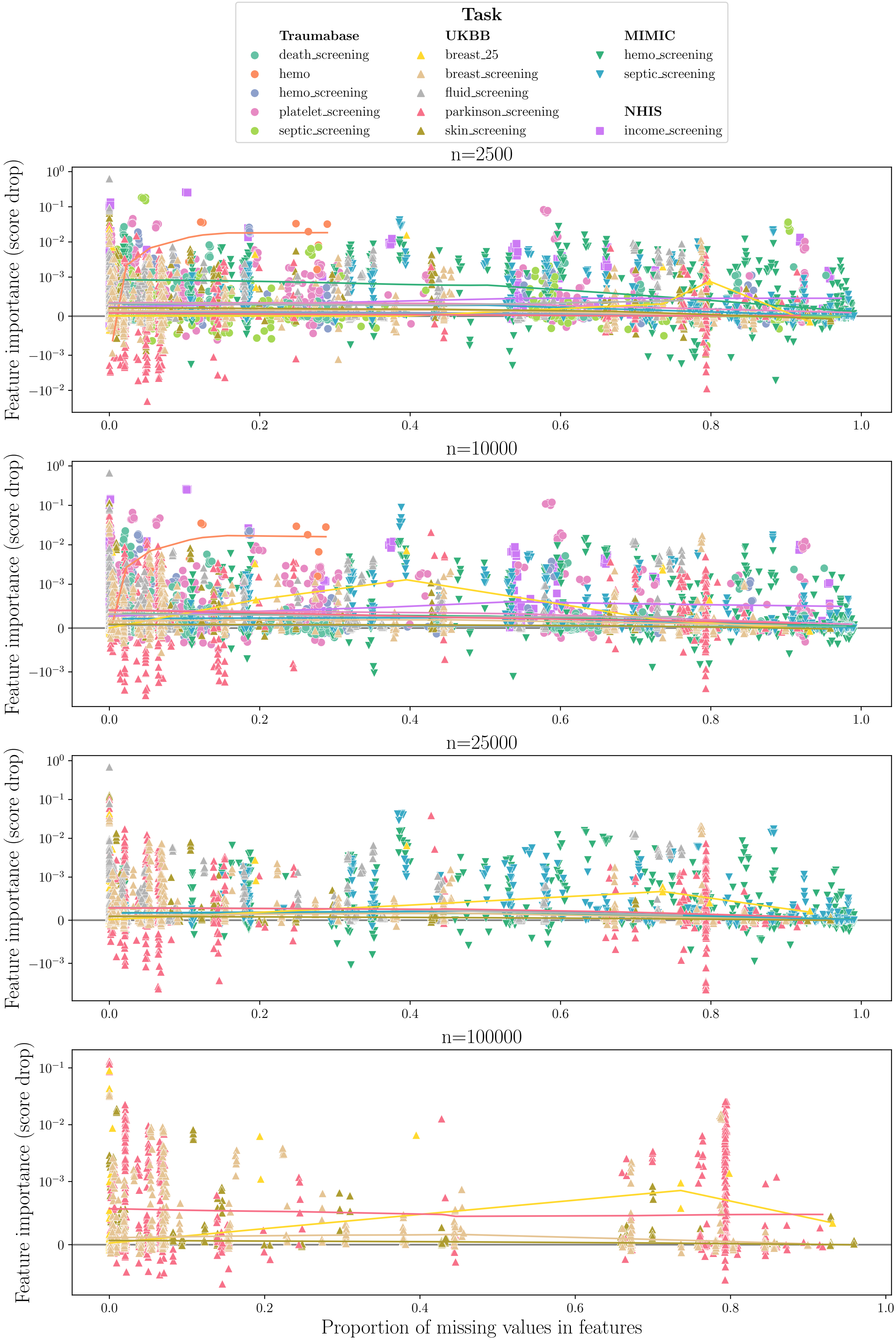

Figure 8. Feature importance versus proportion of missing values. Importance is measured as the drop in score when randomly permuting the considered feature. Each feature is permuted 10 times and its importance is taken as the average drop in score. Score drops are also averaged across folds. Local regressions (LOWESS) are plotted for each task to better visualize trends.

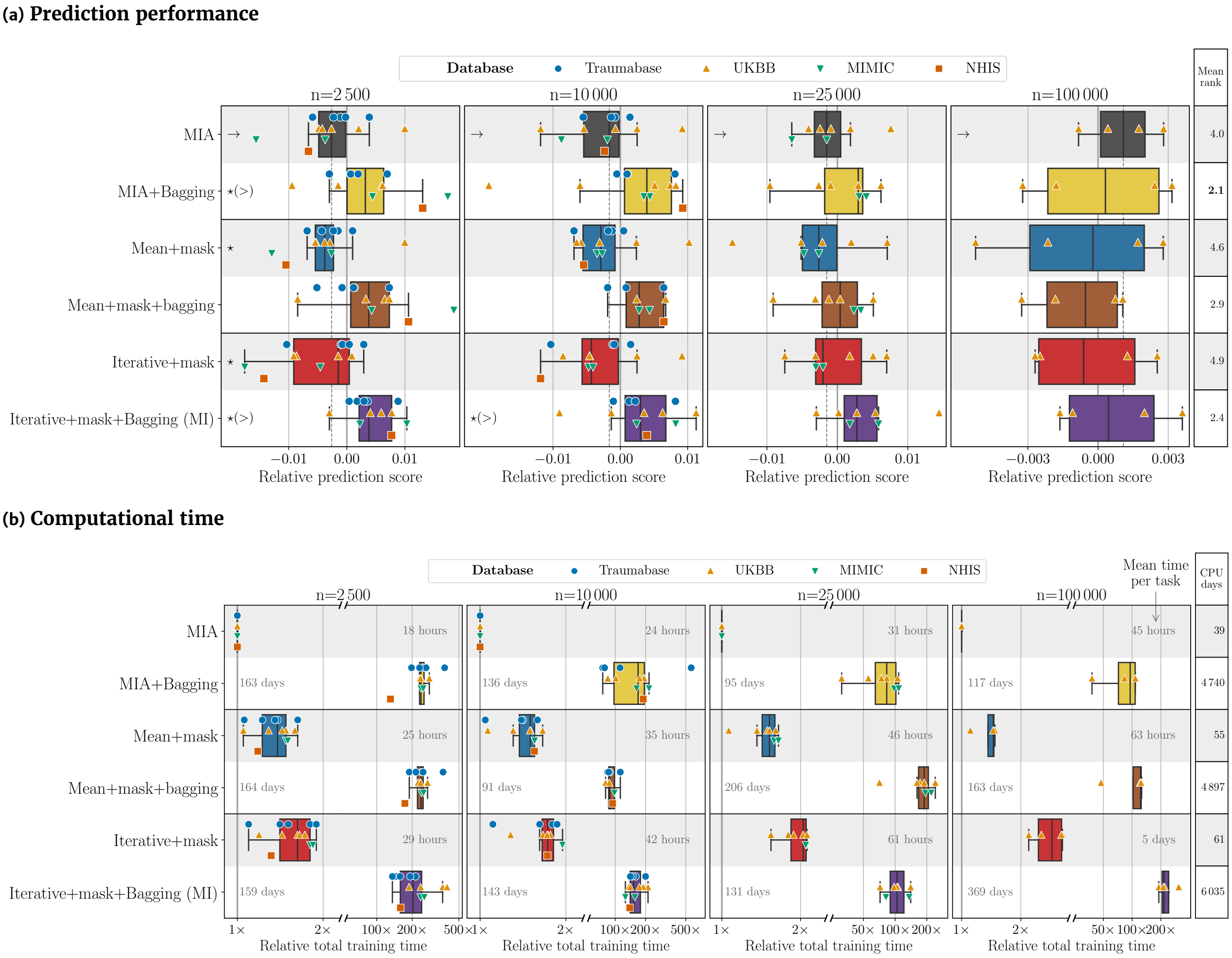

Figure 9. Effect of bagging. Comparison of prediction performance and training times between MIA, Mean+mask, Iterative+mask and their bagged version, for 13 prediction tasks spread over 4 databases, and for 4 sizes of dataset (2 500, 10 000, 25 000 and 100 000 samples). This figure is based on Figure 1, refer to caption of Figure 1 for more details.

Table 7. Overview of the prediction tasks used in this article. For selection, 'A' means ANOVA and 'M' means manual. Type 'C' is classification and type 'R' is regression. The number of features is given after encoding and selection. Since this number may vary between trials, we average it on the 5 trials for the ANOVA selection. Target is the name of the feature to predict in the original database or a formula to build a new feature to predict from the existing ones.

Database	Task	Score	Scorer	Selection	Number of samples	Number of features	Type	Target	Description
Traumabase	death_screening	0.96	AUC	A	12341	98	C	Décès == Oui	Predict the death of patients.
	hemo	0.85	AUC	M	19569	12	C	Choc hémorragique (? 4 CGR sur 6h) == Oui	Predict the hemorrhagic shock using features defined in Jiang et al. (2020).
	hemo_screening	0.95	AUC	A	13047	89	C	Choc hémorragique (? 4 CGR sur 6h) == Oui	Predict the hemorrhagic shock using ANOVA selection.
	platelet_screening	0.17	R2	A	12696	96	R	Plaquettes	Predict the level of platelet on arrival at the hospital using ANOVA selection.
	septic_screening	0.86	AUC	A	6046	90	C	Choc septique == Oui	Predict septic shock.
UKBB	breast_25	0.60	AUC	M	273384	66	C	One of: C500, C501, C502, C503, C504, C505, C506, C508, C509; found in one of: 41270-0.0, 41202-0.0, 41204-0.0, 40006-0.0; or one of: 1740, 1743, 1744, 1745, 1748, 1749; found in one of: 41271-0.0, 41203-0.0, 41205-0.0, 40013-0.0	Predict malignant neoplasm of breast on female patients only using features defined in Läll et al. (2019).
	breast_screening	0.59	AUC	A	182257	100	C	Same as breast_25	Predict malignant neoplasm of breast on female patients only using ANOVA selection.
	fluid_screening	0.56	R2	A	110308	100	R	20016-0.0	Predict the fluid intelligence score.
	parkinson_screening	0.65	AUC	A	335005	100	C	One of: G20, G210, G211, G212, G213, G214, G218, G219, G22, F023; found in one of: 41270-0.0, 41202-0.0, 41204-0.0, 40006-0.0; or one of: 3320, 3321; found in one of: 41271-0.0, 41203-0.0, 41205-0.0, 40013-0.0	Predict Parkinson's disease.
	skin_screening	0.64	AUC	A	335005	100	C	One of: C430, C431, C432, C433, C434, C435, C436, C437, C438, C439, C440, C441, C442, C443, C444, C445, C446, C447, C448, C449; found in one of: 41270-0.0, 41202-0.0, 41204-0.0, 40006-0.0; or one of: 1720, 1723, 1725, 1726, 1727, 1729, 1730, 1731, 1732, 1733, 1734, 1735, 1736, 1737, 1739; found in one of: 41271-0.0, 41203-0.0, 41205-0.0, 40013-0.0	Predict melanoma and other malignant neoplasms of skin.
MIMIC	hemo_screening	0.74	AUC	A	30836	100	C	One of: 78559, 99809, 9584; found in ICD9_CODE	Predict the hemorrhagic shock from the LABEVENTS table only.
	septic_screening	0.87	AUC	A	30836	100	C	ICD9_CODE == 78552	Predict the septic shock from the LABEVENTS table only.
NHIS	income_screening	0.52	R2	A	20987	96	R	ERNYR-P	Predict the income earned on the previous year with information from tables: household, family, person and adult.

Table 8. Scores and ranks of the tree based methods described in Table 1.

(a) Scores relative to the absolute reference score plotted in Figure 1a. Values in bold are the reference scores and are absolute. Other scores are given relative to the reference score of their task and size.

	Database	Traumabase					UKBB					MIMIC		NHIS
Size	Task / Method	death_screening	hemo	hemo_screening	platelet_screening	septic_screening	breast_25	breast_screening	fluid_screening	parkinson_screening	skin_screening	hemo_screening	septic_screening	income_screening
2500	MIA	-8e-5	+5e-3	-1e-3	-4e-4	+4e-3	+7e-3	+2e-3	+5e-5	+2e-3	-3e-3	+3e-3	+8e-3	+3e-3
	Mean	-8e-4	+1e-3	-1e-3	-2e-3	-3e-3	+6e-3	-1e-3	-2e-3	+2e-3	-2e-3	-1e-2	-6e-4	-5e-3
	Mean+mask	-5e-4	+2e-3	-1e-3	-1e-3	+2e-4	+7e-3	-8e-4	-5e-4	-2e-3	-3e-3	+5e-3	+9e-3	-1e-3
	Median	-1e-3	-4e-3	-2e-3	-3e-3	-8e-3	+6e-3	-1e-3	-4e-3	+6e-3	-2e-3	-8e-3	-5e-3	-1e-2
	Median+mask	-2e-4	+3e-3	-2e-3	-1e-3	+7e-4	+7e-3	-9e-4	-1e-3	-2e-3	+1e-3	+2e-3	+1e-2	-3e-3
	Iterative	-5e-4	+2e-3	+1e-3	-5e-3	-2e-3	-2e-3	-5e-3	-6e-3	-2e-3	+4e-3	-2e-2	-2e-2	-7e-3
	Iterative+mask	+4e-6	+4e-3	+3e-4	-5e-3	+5e-3	-2e-3	-4e-3	-4e-3	+1e-3	-4e-4	+6e-4	+7e-3	-5e-3
	KNN	-2e-3	-5e-3	-2e-3	-1e-2	-1e-2	-2e-3	-9e-3	-9e-3	-3e-3	-5e-3	-4e-2	-3e-2	-2e-2
	KNN+mask	-2e-3	-5e-3	-3e-3	-1e-2	-1e-3	-2e-3	-3e-3	-3e-3	-1e-3	-6e-3	-1e-2	+7e-3	-9e-3
	Iterative+Bagging	+2e-3	-1e-3	+5e-3	+1e-2	+2e-3	-6e-3	+5e-3	+8e-3		+8e-3	+1e-2	-1e-2	+1e-2
	Iterative+mask+Bagging	+3e-3	+1e-3	+5e-3	+1e-2	+7e-3	-6e-3	+9e-3	+1e-2		+9e-3	+3e-2	+1e-2	+2e-2
	MIA+Bagging	+2e-3	-2e-3	+2e-3	+1e-2	+6e-3	-1e-2	+1e-2	+1e-2		-4e-4	+4e-2	+2e-2	+2e-2
	Reference score	**0.97**	**0.85**	**0.96**	**0.19**	**0.89**	**0.62**	**0.55**	**0.57**	**0.55**	**0.62**	**0.77**	**0.90**	**0.54**
10000	MIA	+2e-4	+4e-3	-4e-4	+3e-4		+5e-3	+4e-3	-1e-5	-9e-3	+1e-3	-1e-4	+6e-3	+5e-3
	Mean	-6e-4	-3e-4	-1e-3	-8e-4		+6e-3	-3e-3	-3e-3	+3e-4	+2e-3	-3e-3	-2e-4	-2e-3
	Mean+mask	-2e-4	+3e-3	-8e-4	-1e-3		+6e-3	-1e-3	-1e-3	-6e-4	+1e-3	+5e-3	+6e-3	+2e-3
	Median	-1e-3	-8e-4	-2e-3	-4e-3		+8e-3	-3e-3	-4e-3	-1e-2	+1e-3	-1e-2	-2e-3	-1e-2
	Median+mask	-1e-4	+2e-3	-1e-3	-3e-3		+7e-3	+2e-3	-1e-3	+8e-3	+1e-3	+3e-3	+6e-3	+1e-3
	Iterative	-6e-4	-3e-4	+8e-5	-6e-3		+6e-3	-7e-3	-7e-3	+1e-2	+1e-3	-1e-2	-1e-2	-8e-3
	Iterative+mask	-1e-4	+4e-3	+9e-5	-5e-3		+5e-3	-1e-4	-3e-3	-2e-3	+1e-3	+4e-3	+4e-3	-4e-3
	KNN	-2e-3	-9e-3	-2e-3	-1e-2		+2e-3	-9e-3	-9e-3	-2e-2	+1e-3	-2e-2	-3e-2	-1e-2
	KNN+mask	-1e-3	-3e-3	-1e-3	-9e-3		+2e-3	-2e-3	-4e-3	-1e-2	+1e-3	+1e-4	+4e-3	-5e-3
	Iterative+Bagging	+2e-3	-1e-3	+3e-3	+1e-2		-1e-2	+2e-3	+8e-3	+2e-2	-3e-3	+7e-3	-6e-3	+7e-3
	Iterative+mask+Bagging	+2e-3	+1e-3	+3e-3	+1e-2		-1e-2	+8e-3	+1e-2	+1e-2	-2e-3	+2e-2	+1e-2	+1e-2
	MIA+Bagging	+2e-3	+2e-3	+2e-3	+1e-2		-2e-2	+1e-2	+1e-2	+1e-2	-7e-3	+1e-2	+1e-2	+2e-2
	Reference score	**0.97**	**0.86**	**0.96**	**0.22**		**0.64**	**0.59**	**0.60**	**0.63**	**0.67**	**0.82**	**0.92**	**0.57**
25000	MIA						+3e-3	+3e-3	+9e-4	-1e-3	+9e-4	+8e-4	+6e-3	
	Mean						+4e-3	-2e-3	-2e-3	-5e-3	+9e-4	-1e-3	+5e-4	
	Mean+mask						+3e-3	+2e-3	-1e-4	-1e-2	+1e-3	+2e-3	+5e-3	
	Median						+5e-3	-2e-3	-4e-3	-5e-3	+5e-4	-5e-3	-2e-3	
	Median+mask						+5e-3	+2e-3	-1e-3	-1e-2	+9e-4	+1e-3	+6e-3	
	Iterative						+3e-3	-7e-3	-7e-3	+4e-3	+5e-4	-9e-3	-1e-2	
	Iterative+mask						+3e-3	+8e-4	-2e-3	+6e-3	+8e-4	+5e-3	+5e-3	
	KNN						+2e-3	-1e-2	-9e-3			-2e-2	-2e-2	
	KNN+mask						+1e-3	-1e-4	-4e-3			+6e-4	+4e-3	
	Iterative+Bagging						-7e-3	-8e-4	+7e-3	+1e-2	-1e-3	+3e-3	-8e-3	
	Iterative+mask+Bagging						-7e-3	+7e-3	+1e-2	+2e-2	-7e-4	+1e-2	+1e-2	
	MIA+Bagging						-1e-2	+7e-3	+1e-2	+4e-4	-4e-3	+1e-2	+1e-2	
	Reference score						**0.65**	**0.61**	**0.62**	**0.68**	**0.68**	**0.84**	**0.93**	
100000	MIA						+2e-3	+2e-3		+3e-3	+1e-3			
	Mean						+1e-3	-2e-3		-5e-3	+7e-4			
	Mean+mask						+2e-3	+9e-4		-2e-3	+1e-3			
	Median						+2e-3	-2e-3		-6e-3	+5e-4			
	Median+mask						+2e-3	+9e-4		-5e-3	+1e-3			
	Iterative						+1e-3	-9e-3		-4e-3	+2e-4			
	Iterative+mask						+1e-3	+6e-4		+1e-4	+8e-4			
	KNN													
	KNN+mask													
	Iterative+Bagging						-3e-3	-2e-3		+6e-3	-2e-3			
	Iterative+mask+Bagging						-3e-3	+5e-3		+6e-3	-2e-3			
	MIA+Bagging						-4e-3	+5e-3		+6e-3	-2e-3			
	Reference score						**0.66**	**0.63**		**0.76**	**0.69**			
Average	Reference score	**0.97**	**0.85**	**0.96**	**0.20**	**0.89**	**0.64**	**0.59**	**0.60**	**0.66**	**0.66**	**0.81**	**0.92**	**0.56**

(b) Ranks computed from the relative scores in Supplementary Table 8a. Best average ranks are in bold.

Size	Database / Task / Method	Traumabase: death_screening	Traumabase: hemo	Traumabase: hemo_screening	Traumabase: platelet_screening	Traumabase: septic_screening	UKBB: breast_25	UKBB: breast_screening	UKBB: fluid_screening	UKBB: parkinson_screening	UKBB: skin_screening	MIMIC: hemo_screening	MIMIC: septic_screening	NHIS: income_screening	Average: Traumabase	Average: UKBB	Average: MIMIC	Average: NHIS	Average: All
2500	MIA	5	1	6	4	4	1	4	4	2	10	5	5	4	4.0	4.2	5.0	4.0	4.3
	Mean	9	6	8	7	10	5	8	7	3	7	9	8	8	8.0	6.0	8.5	8.0	7.6
	Mean+mask	7	5	7	5	7	2	5	5	8	9	4	4	5	6.2	5.8	4.0	5.0	5.2
	Median	10	10	10	8	11	4	7	9	1	8	8	9	11	9.8	5.8	8.5	11.0	8.8
	Median+mask	6	3	11	6	6	3	6	6	6	4	6	3	6	6.4	5.0	4.5	6.0	5.5
	Iterative	8	4	4	9	9	9	11	11	7	3	11	11	9	6.8	8.2	11.0	9.0	8.8
	Iterative+mask	4	2	5	10	3	8	10	10	4	5	7	6	7	4.8	7.4	6.5	7.0	6.4
	KNN	12	12	9	12	12	7	12	12	9	11	12	12	12	11.4	10.2	12.0	12.0	11.4
	KNN+mask	11	11	12	11	8	6	9	8	5	12	10	7	10	10.6	8.0	8.5	10.0	9.3
	Iterative+Bagging	2	8	2	2	5	10	3	3		2	3	10	3	3.8	4.5	6.5	3.0	4.4
	Iterative+mask+Bagging	1	7	1	1	1	11	2	2		1	2	2	2	**2.2**	**4.0**	2.0	2.0	**2.6**
	MIA+Bagging	3	9	3	3	2	12	1	1		6	1	1	1	4.0	5.0	**1.0**	**1.0**	2.8
10000	MIA	4	2	6	4		6	3	4	9	2	8	3	4	4.0	**4.8**	5.5	4.0	4.6
	Mean	8	7	8	5		3	9	7	6	1	9	8	7	7.0	5.2	8.5	7.0	6.9
	Mean+mask	7	3	7	6		4	7	5	7	5	4	5	5	5.8	5.6	4.5	5.0	5.2
	Median	10	9	12	8		1	10	10	10	4	10	9	11	9.8	7.0	9.5	11.0	9.3
	Median+mask	6	4	10	7		2	5	6	5	7	6	4	6	6.8	5.0	5.0	6.0	5.7
	Iterative	9	8	5	10		5	11	11	3	8	11	11	10	8.0	7.6	11.0	10.0	9.2
	Iterative+mask	5	1	4	9		7	6	8	8	3	5	6	8	4.8	6.4	5.5	8.0	6.2
	KNN	12	12	11	12		9	12	12	12	9	12	12	12	11.8	10.8	12.0	12.0	11.6
	KNN+mask	11	11	9	11		8	8	9	11	6	7	7	9	10.5	8.4	7.0	9.0	8.7
	Iterative+Bagging	3	10	2	3		10	4	3	1	11	3	10	3	4.5	5.8	6.5	3.0	5.0
	Iterative+mask+Bagging	1	6	1	1		11	2	2	2	10	1	2	2	**2.2**	5.4	**1.5**	2.0	**2.8**
	MIA+Bagging	2	5	3	2		12	1	1	4	12	2	1	1	3.0	6.0	1.5	**1.0**	2.9
25000	MIA						4	3	4	6	2	7	3			**3.8**	5.0		4.4
	Mean						3	10	7	8	4	9	8			6.4	8.5		7.4
	Mean+mask						6	4	5	10	1	5	5			5.2	5.0		5.1
	Median						2	9	10	7	6	10	9			6.8	9.5		8.2
	Median+mask						1	5	6	9	3	6	4			4.8	5.0		4.9
	Iterative						5	11	11	4	7	11	11			7.6	11.0		9.3
	Iterative+mask						7	6	8	3	5	3	6			5.8	4.5		5.2
	KNN						8	12	12			12	12			10.7	12.0		11.3
	KNN+mask						9	7	9			8	7			8.3	7.5		7.9
	Iterative+Bagging						11	8	3	2	9	4	10			6.6	7.0		6.8
	Iterative+mask+Bagging						10	2	2	1	8	1	2			4.6	**1.5**		**3.0**
	MIA+Bagging						12	1	1	5	10	2	1			5.8	1.5		3.6
100000	MIA						2	3		4	1					**2.5**			**2.5**
	Mean						7	8		9	5					7.2			7.2
	Mean+mask						3	5		6	2					4.0			4.0
	Median						4	9		10	6					7.2			7.2
	Median+mask						1	4		8	3					4.0			4.0
	Iterative						5	10		7	7					7.2			7.2
	Iterative+mask						6	6		5	4					5.2			5.2
	KNN																		
	KNN+mask																		
	Iterative+Bagging						9	7		1	10					6.8			6.8
	Iterative+mask+Bagging						8	2		2	8					5.0			5.0
	MIA+Bagging						10	1		3	9					5.8			5.8
Average	MIA	4.5	**1.5**	6.0	4.0	4.0	3.2	3.2	4.0	5.2	**3.8**	6.7	3.7	4.0	4.0	**3.9**	5.2	4.0	4.3
	Mean	8.5	6.5	8.0	6.0	10.0	4.5	8.8	7.0	6.5	4.2	9.0	8.0	7.5	7.8	6.2	8.5	7.5	7.5
	Mean+mask	7.0	4.0	7.0	5.5	7.0	3.8	5.2	5.0	7.8	4.2	4.3	4.7	5.0	6.1	5.2	4.5	5.0	5.2
	Median	10.0	9.5	11.0	8.0	11.0	2.8	8.8	9.7	7.0	6.0	9.3	9.0	11.0	9.9	6.8	9.2	11.0	9.2
	Median+mask	6.0	3.5	10.5	6.5	6.0	**1.8**	5.0	6.0	7.0	4.2	6.0	3.7	6.0	6.5	4.8	4.8	6.0	5.5
	Iterative	8.5	6.0	4.5	9.5	9.0	6.0	10.8	11.0	5.2	6.2	11.0	11.0	9.5	7.5	7.8	11.0	9.5	9.0
	Iterative+mask	4.5	1.5	4.5	9.5	3.0	7.0	7.0	8.7	5.0	4.2	5.0	6.0	7.5	4.6	6.4	5.5	7.5	6.0
	KNN	12.0	12.0	10.0	12.0	12.0	8.0	12.0	12.0	10.5	10.0	12.0	12.0	12.0	11.6	10.5	12.0	12.0	11.5
	KNN+mask	11.0	11.0	10.5	11.0	8.0	7.7	8.0	8.7	8.0	9.0	8.3	7.0	9.5	10.3	8.3	7.7	9.5	8.9
	Iterative+Bagging	2.5	9.0	2.0	2.5	5.0	10.0	5.5	3.0	**1.3**	8.0	3.3	10.0	3.0	4.2	5.6	6.7	3.0	4.9
	Iterative+mask+Bagging	**1.0**	6.5	**1.0**	**1.0**	**1.0**	10.0	2.0	2.0	1.7	6.8	**1.3**	2.0	2.0	**2.1**	4.5	1.7	2.0	**2.6**
	MIA+Bagging	2.5	7.0	3.0	2.5	2.0	11.5	**1.0**	**1.0**	4.0	9.2	1.7	**1.0**	**1.0**	3.4	5.4	**1.3**	**1.0**	2.8

Table 9. Scores and ranks of gradient-boosted trees+MIA compared to linear methods described in Supplementary Table 2.
We removed one outlier fold from one trial for the methods Linear+Iter and Linear+Iter+mask for the "task platelet_screening" at size n=2 500. Others are unchanged.

(a) Scores relative to the absolute reference score and plotted in Supplementary Figure 2a. Values in bold are the reference scores and are absolute. Other scores are given relative to the reference score of their task and size.

	Database	Traumabase					UKBB					MIMIC		NHIS
Size	Task<br>Method	death_screening	hemo	hemo_screening	platelet_screening	septic_screening	breast_25	breast_screening	fluid_screening	parkinson_screening	skin_screening	hemo_screening	septic_screening	income_screening
2500	Boosted trees+MIA	+1e-2	+9e-3	+2e-2	+1e-1	+5e-2	+8e-2	-2e-3	+6e-2	-8e-3	+2e-2	+1e-1	+1e-1	+5e-2
	Linear+Mean	+8e-4	-3e-3	-3e-3	+8e-3	-4e-3	-6e-3	+2e-4	-1e-2	+2e-3	-6e-3	-1e-2	-2e-2	-2e-2
	Linear+Mean+mask	+6e-4	+2e-4	-2e-3	-2e-3	-4e-3	-5e-4	+1e-3	+8e-4	+2e-3	-4e-3	-2e-2	-1e-2	+7e-3
	Linear+Med	-7e-4	-8e-3	-3e-3	+6e-3	-4e-3	+2e-3	+3e-4	-1e-2	-5e-4	-5e-3	-2e-2	+1e-2	-2e-2
	Linear+Med+mask	-3e-4	-7e-3	-3e-3	-2e-3	-4e-3	+3e-3	+8e-4	+8e-4	-3e-4	-3e-3	-1e-2	+1e-2	+7e-3
	Linear+Iter	+2e-3	+4e-3	+8e-5	-5e-2	-6e-6	-1e-2	+3e-3	-2e-2	+2e-2	+9e-3	-1e-2	-3e-2	-2e-2
	Linear+Iter+mask	-2e-2	+7e-3	-5e-4	-8e-2	+1e-3	-1e-2	+2e-3	-7e-3	+2e-2	+1e-2	-2e-2	-3e-2	+3e-3
	Linear+KNN	+1e-3	-3e-3	-5e-3	+9e-3	-2e-2	-3e-2	-2e-3	-8e-3	-1e-2	-1e-2	-2e-2	-2e-2	-2e-2
	Linear+KNN+mask	+7e-4	+8e-4	-4e-3	+5e-3	-2e-2	-3e-2	-3e-3	+2e-3	-1e-2	-1e-2	-2e-2	-2e-2	+3e-3
	Reference score	**0.96**	**0.84**	**0.94**	**0.09**	**0.84**	**0.55**	**0.56**	**0.51**	**0.56**	**0.59**	**0.64**	**0.81**	**0.49**
10000	Boosted trees+MIA	+2e-2	+1e-2	+3e-2	+4e-2		+9e-2	+1e-2	+7e-2	-1e-2	+6e-2	+1e-1	+8e-2	+7e-2
	Linear+Mean	+1e-2	-9e-4	+8e-4	-4e-3		-1e-2	+8e-4	-2e-2	+2e-4	-1e-2	-1e-2	-2e-2	-2e-2
	Linear+Mean+mask	+1e-2	+4e-3	+9e-4	+1e-3		-1e-2	-2e-4	+1e-3	+7e-4	-7e-3	-2e-2	-1e-2	+1e-2
	Linear+Med	+9e-3	-6e-3	+5e-4	-6e-3		-1e-2	-7e-4	-2e-2	-5e-3	-9e-3	-8e-3	+8e-3	-2e-2
	Linear+Med+mask	+9e-3	+2e-3	+5e-4	+1e-3		-1e-2	+9e-4	+1e-3	-5e-3	-1e-2	-9e-3	+1e-2	+1e-2
	Linear+Iter	-3e-2	-1e-3	-2e-2	-1e-2		-2e-3	-3e-3	-3e-2	+3e-2	+1e-2	-2e-2	-2e-2	-4e-2
	Linear+Iter+mask	-5e-2	+5e-3	-1e-2	-6e-3		-1e-3	-3e-3	-7e-3	+3e-2	+9e-3	-7e-3	-2e-2	-6e-4
	Linear+KNN	+1e-2	-9e-3	-1e-3	-8e-3		-2e-2	-3e-3	-9e-3	-2e-2	-2e-2	-2e-2	-2e-2	-2e-2
	Linear+KNN+mask	+1e-2	-5e-3	-9e-4	-4e-3		-2e-2	-3e-3	+4e-3	-1e-2	-2e-2	-2e-2	-2e-2	+5e-3
	Reference score	**0.95**	**0.85**	**0.94**	**0.18**		**0.56**	**0.58**	**0.53**	**0.63**	**0.61**	**0.70**	**0.84**	**0.51**
25000	Boosted trees+MIA						+8e-2	+2e-2	+8e-2	-7e-3	+6e-2	+1e-1	+8e-2	
	Linear+Mean						-1e-2	+9e-4	-2e-2	-7e-3	-1e-2	-2e-2	-2e-2	
	Linear+Mean+mask						-1e-2	+1e-3	+1e-3	-7e-3	-7e-3	-1e-2	-1e-2	
	Linear+Med						-1e-2	-9e-4	-2e-2	-9e-3	-2e-2	-4e-3	+6e-3	
	Linear+Med+mask						-7e-3	-1e-3	+1e-3	-6e-3	-1e-2	-1e-2	+9e-3	
	Linear+Iter						-5e-3	-8e-3	-3e-2	+2e-2	+1e-2	-1e-4	-1e-2	
	Linear+Iter+mask						-9e-4	-8e-3	-8e-3	+2e-2	+1e-2	+1e-3	-1e-2	
	Linear+KNN						-2e-2	-2e-3	-1e-2		-4e-2	-3e-2	-2e-2	
	Linear+KNN+mask						-2e-2	-2e-3	+3e-3			-3e-2	-2e-2	
	Reference score						**0.57**	**0.59**	**0.54**	**0.69**	**0.62**	**0.73**	**0.85**	
100000	Boosted trees+MIA						+6e-2	+3e-2		+2e-2	+5e-2			
	Linear+Mean						-1e-2	-8e-4		-5e-3	-2e-2			
	Linear+Mean+mask						-8e-3	-1e-3		-3e-3	-2e-2			
	Linear+Med						-9e-3	-2e-3		-7e-3	-2e-2			
	Linear+Med+mask						-6e-3	-4e-3		-6e-3	-2e-2			
	Linear+Iter						-1e-2	-8e-3		-4e-4	+9e-3			
	Linear+Iter+mask						-1e-2	-7e-3		+4e-4	+9e-3			
	Linear+KNN							-3e-3						
	Linear+KNN+mask							-1e-3						
	Reference score						**0.60**	**0.60**		**0.74**	**0.64**			
Average	Reference score	**0.96**	**0.85**	**0.94**	**0.14**	**0.84**	**0.57**	**0.58**	**0.53**	**0.66**	**0.62**	**0.69**	**0.83**	**0.50**

(b) Ranks computed from relative scores in Supplementary Table 9a. Best average ranks are in bold.

	Database	Traumabase					UKBB					MIMIC		NHIS	Average				
Size	Task / Method	death_screening	hemo	hemo_screening	platelet_screening	septic_screening	breast_25	breast_screening	fluid_screening	parkinson_screening	skin_screening	hemo_screening	septic_screening	income_screening	Traumabase	UKBB	MIMIC	NHIS	All
2500	Boosted trees+MIA	1	1	1	1	1	1	7	1	7	1	1	1	1	**1.0**	**3.4**	**1.0**	**1.0**	**1.6**
	Linear+Mean	4	7	6	3	6	5	6	7	3	7	3	6	6	5.2	5.6	4.5	6.0	5.3
	Linear+Mean+mask	6	5	4	7	4	4	3	3	4	5	5	4	2	5.2	3.8	4.5	2.0	3.9
	Linear+Med	8	9	7	4	5	3	5	8	6	6	7	3	8	6.6	5.6	5.0	8.0	6.3
	Linear+Med+mask	7	8	5	6	7	2	4	4	5	4	4	2	3	6.6	3.8	3.0	3.0	4.1
	Linear+Iter	2	3	2	8	3	7	1	9	1	3	2	9	7	3.6	4.2	5.5	7.0	5.1
	Linear+Iter+mask	9	2	3	9	2	6	2	5	2	2	6	8	4	5.0	3.4	7.0	4.0	4.8
	Linear+KNN	3	6	9	2	9	9	8	6	9	9	9	5	9	5.8	8.2	7.0	9.0	7.5
	Linear+KNN+mask	5	4	8	5	8	8	9	2	8	8	8	7	5	6.0	7.0	7.5	5.0	6.4
10000	Boosted trees+MIA	1	1	1	1		1	1	1	7	1	1	1	1	**1.0**	**2.2**	**1.0**	**1.0**	**1.3**
	Linear+Mean	4	5	3	4		7	3	7	4	6	5	6	6	4.0	5.4	5.5	6.0	5.2
	Linear+Mean+mask	5	3	2	3		6	4	4	3	4	7	4	2	3.2	4.2	5.5	2.0	3.7
	Linear+Med	7	8	4	6		5	5	8	6	5	3	3	7	6.2	5.8	3.0	7.0	5.5
	Linear+Med+mask	6	4	5	2		4	2	3	5	7	4	2	3	4.2	4.2	3.0	3.0	3.6
	Linear+Iter	8	6	9	9		3	7	9	2	2	6	8	9	8.0	4.6	7.0	9.0	7.2
	Linear+Iter+mask	9	2	8	7		2	8	5	1	3	2	5	5	6.5	3.8	3.5	5.0	4.7
	Linear+KNN	2	9	7	8		9	6	6	9	8	9	9	8	6.5	7.6	9.0	8.0	7.8
	Linear+KNN+mask	3	7	6	5		8	9	2	8	9	8	7	4	5.2	7.2	7.5	4.0	6.0
25000	Boosted trees+MIA						1	1	1	5	1	1	1			**1.8**	**1.0**		**1.4**
	Linear+Mean						7	3	7	6	5	7	8			5.6	7.5		6.6
	Linear+Mean+mask						5	2	4	4	4	6	6			3.8	6.0		4.9
	Linear+Med						6	4	8	7	7	4	3			6.4	3.5		5.0
	Linear+Med+mask						4	5	3	3	6	5	2			4.2	3.5		3.8
	Linear+Iter						3	9	9	1	3	3	5			5.0	4.0		4.5
	Linear+Iter+mask						2	8	5	2	2	2	4			3.8	3.0		3.4
	Linear+KNN						9	6	6		8	9	9			7.2	9.0		8.1
	Linear+KNN+mask						8	7	2			8	7			5.7	7.5		6.6
100000	Boosted trees+MIA						1	1		1	1					**1.0**			**1.0**
	Linear+Mean						6	2		5	5					4.5			4.5
	Linear+Mean+mask						3	4		4	4					3.8			3.8
	Linear+Med						4	5		7	7					5.8			5.8
	Linear+Med+mask						2	7		6	6					5.2			5.2
	Linear+Iter						5	9		3	3					5.0			5.0
	Linear+Iter+mask						7	8		2	2					4.8			4.8
	Linear+KNN							6								6.0			6.0
	Linear+KNN+mask							3								3.0			3.0
Average	Boosted trees+MIA	**1.0**	**1.0**	**1.0**	**1.0**	**1.0**	**1.0**	**2.5**	**1.0**	5.0	**1.0**	**1.0**	**1.0**	**1.0**	**1.0**	**2.1**	**1.0**	**1.0**	**1.3**
	Linear+Mean	4.0	6.0	4.5	3.5	6.0	6.2	3.5	7.0	4.5	5.8	5.0	6.7	6.0	4.8	5.4	5.8	6.0	5.5
	Linear+Mean+mask	5.5	4.0	3.0	5.0	4.0	4.5	3.2	3.7	3.8	4.2	6.0	4.7	2.0	4.3	3.9	5.3	2.0	3.9
	Linear+Med	7.5	8.5	5.5	5.0	5.0	4.5	4.8	8.0	6.5	6.2	4.7	3.0	7.5	6.3	6.0	3.8	7.5	5.9
	Linear+Med+mask	6.5	6.0	5.0	4.0	7.0	3.0	4.5	3.3	4.8	5.8	4.3	2.0	3.0	5.7	4.3	3.2	3.0	4.0
	Linear+Iter	5.0	4.5	5.5	8.5	3.0	4.5	6.5	9.0	**1.8**	2.8	3.7	7.3	8.0	5.3	4.9	5.5	8.0	5.9
	Linear+Iter+mask	9.0	2.0	5.5	8.0	2.0	4.2	6.5	5.0	1.8	2.2	3.3	5.7	4.5	5.3	4.0	4.5	4.5	4.6
	Linear+KNN	2.5	7.5	8.0	5.0	9.0	9.0	6.5	6.0	9.0	8.3	9.0	7.7	8.5	6.4	7.8	8.3	8.5	7.8
	Linear+KNN+mask	4.0	5.5	7.0	5.0	8.0	8.0	7.0	2.0	8.0	8.5	8.0	7.0	4.5	5.9	6.7	7.5	4.5	6.2

Manuscript with difference from previous submission Click here to access/download;Manuscript;diff_manuscript.pdf

OXFORD (GIGA)^n SCIENCE

GigaScience, XXXX, 1–23

doi: xx.xxxx/xxxx
Manuscript in Preparation
Paper

PAPER

Benchmarking missing-values approaches for predictive models on health databases.

Alexandre Perez-Lebel[1,2,3,*], Gaël Varoquaux[1,2,3], Marine Le Morvan[2], Julie Josse[4] and Jean-Baptiste Poline[1]

[1]McConnell Brain Imaging Centre, The Neuro (Montreal Neurological Institute-Hospital), Faculty of Medicine, McGill University, Montreal, QC, Canada and [2]Inria, Palaiseau, France and [3]Mila, Montreal, QC, Canada and [4]Inria, Montpellier, France

[*]alexandre.perez@inria.fr

Abstract

BACKGROUND As databases grow larger, it becomes harder to fully control their collection, and they frequently come with missing values: incomplete observations. These large databases are well suited to train machine-learning models, for instance for forecasting or to extract biomarkers in biomedical settings. Such predictive approaches can use discriminative –rather than generative– modeling, and thus open the door to new missing-values strategies. Yet existing empirical evaluations of strategies to handle missing values have focused on inferential statistics. **RESULTS** Here we conduct a systematic benchmark of missing-values strategies in predictive models with a focus on large health databases: ~~two~~ four electronic health record datasets, a population brain imaging one, ~~and~~ a health survey and two intensive care ones. Using gradient-boosted trees, we compare native support for missing values with simple and state-of-the-art imputation prior to learning. We investigate prediction accuracy and computational time. For prediction after imputation, we find that adding an indicator to express which values have been imputed is important, suggesting that the data are missing not at random. Elaborate missing values imputation can improve prediction compared to simple strategies but requires longer computational time on large data. Learning trees that model missing values –with missing incorporated attribute– leads to robust, fast, and well-performing predictive modeling. **CONCLUSIONS** Native support for missing values in supervised machine learning predicts better than state-of-the-art imputation with much less computational cost. When using imputation, it is important to add indicator columns expressing which values have been imputed.

Key words: Missing values; machine learning; supervised learning; benchmark; imputation; multiple imputation; bagging

Background: missing values in databases

Missing values are pervasive in many application domains. This is particularly true on health data, where missing values arise for a multitude of reasons: two patients rarely follow the same medical path and take the exact same set of exams; measurements are omitted because of lack of time or because the patient's condition does not allow it; hospitals do not collect exactly the same information because of diverging practices and the use of different devices; etc. This problem is exacerbated when the data are aggregated across multiple sources or when each individual sample comprises many features. The more data there is, the more data is missing.

There is a rich and established statistical literature for the treatment of missing data (Little and Rubin; 2019; Wells et al.; 2013), which has so far been mostly focused on inferential purposes, ~~*i.e.*~~ *i.e.* estimating parameters of a probabilistic model with their confidence intervals. For such problem, an

Compiled on: November 30, 2021.
Draft manuscript prepared by the author.

Key Points

- Benchmarks on health databases highlight the challenges that they ~~present~~ represent for statistical learning: non-ignorable missing values (Missing Not At Random – MNAR), non-linear relationships between covariates and ~~oucomes~~outcomes.
- Native missing-values support in supervised machine learning gives better prediction than state-of-the-art imputation with significantly less computational cost.
- With linear models, conditional imputation is to be preferred.
- When using imputation, concatenating the missingness indicator with the input features significantly improves predictions.
- Bagging, as sometimes used for multiple-imputation, improves prediction performance but with a prohibitive time cost.

important distinction between missing data mechanisms was introduced by Rubin (1976): Missing Completely At Random (MCAR) where the probability of having missing data does not depend on the covariates, Missing At Random (MAR), where the probability of a missing ~~values~~ value only depends on the observed values of other variables; and Missing Not At Random (MNAR) which covers all other cases. MNAR corresponds to cases where the missingness ~~caries~~ carries information. For example, if heartbeat measures are not reported when the values are too low, it creates a MNAR situation. Most available methods for inference in the presence of missing values are only valid under the MAR assumption, including maximum likelihood approaches with the ~~expectation maximization~~ Expectation Maximization algorithm (Dempster et al.; 1977), as well as ~~multiple imputation~~ Multiple Imputation (Van Buuren; 2018). The latter is a ~~two steps~~ two-step approach where the data is first imputed multiple times to create multiple completed datasets, and then the analysis is performed on each imputed dataset separately before combining the results to take into account the uncertainty due to missing values.

Supervised learning to build models that predict best a response using covariates with missing values can lead to different tradeoff than inference models (Sperrin et al.; 2020; Josse et al.; 2019). In health, such predictive models are central to building complex biomarkers or risk scores, to forecasting an epidemic, and they can even underlie causal inference for policy evaluation (Rose and Rizopoulos; 2020). They are increasingly used on electronic health records ~~Miotto et al. (2016); Zheng et al. (2017); Steele et al. (2018)~~ (Miotto et al.; 2016; Zheng et al.; 2017; Steele et al.; 2018) , where the choice of strategy to handle missing values remains a challenge (Jarrett et al.; 2021). Indeed, unlike with inference, little work to date has focused on the systematic evaluation of supervised learning with missing values. Existing works focus on benchmarking imputation quality (Jäger et al.; 2021; Bertsimas et al.; 2018) – which, as our study points out, is a different goal than prediction quality – or only focus on imputation-based methods (Poulos and Valle; 2018).

In practice, a number of options are commonly used to learn predictive models with missing values. The simplest one is to delete all observations containing missing values. However, leaving aside the possible biases that this practice may induce, it often leads to considerable loss of information in high and even moderate dimensions. Indeed, when there are many variables, it is common that only a few observations are completely observed.

In order to deal with arbitrary subsets of input features, the most common practice currently consists in first imputing the missing values, and then learning a predictive model (e.g regression or classification) on the completed data. The popularity of this approach is mainly due to its simplicity and ease of implementation. After imputation, off-the-shelf learners can be applied on the completed dataset. Recent theoretical results show that applying a supervised-learning regression on imputed data can asymptotically recover the optimal prediction function; however most imputation strategies, including the common imputation by the conditional expectation, create discontinuities in the regression function to learn (Le Morvan et al.; 2021).

A small number of machine learning models can natively handle missing values, in particular popular tree-based methods. Trees greedily partition the input space into subspaces in order to minimize a risk. This non-smooth optimization scheme enables them to be easily adapted to directly learn from incomplete data. Several adaptations of trees to missing values have been proposed (see Josse et al.; 2019, for a short review). Missing Incorporated in Attributes (MIA, Twala et al.; 2008) is the most promising strategy (Josse et al.; 2019), described below in the experiment section.

In this work, we benchmark the most popular methods for supervised learning with missing values on multiple large real-world health databases. In contrast to most simulations, real health databases combine a number of challenges: unknown data distributions (not necessarily Gaussian), uncontrolled missing data mechanism (not necessarily MAR), mixed quantitative and categorical data, and often a high level of noise. In such a challenging setting, we compare existing approaches to make recommendations that are directly relevant for the practitioner. To establish general recommendations, we study a total of 13 prediction *real-world* tasks (10 classification and 3 regression tasks) across four publicly-available health databases of very different nature. For each of these tasks, we compare methodologies based on imputation followed by regression or classification, to tree-based models that can natively handle missing values with a MIA strategy. These methods are chosen from the common practice as well as theoretical work on supervised learning with missing values (Josse et al.; 2019).

The present study has several strengths in terms of benchmarking methodology, avoiding common limitations. It uses real data *and* real missingness; multiple draws of a cross-validation loops are used; the imputation procedure is not fitted on the whole dataset but rather on the training set to prevent leaks from the training set to the out-of-sample test set; hyper-parameters of the predictive model are tuned for each method to reduce bias in the hyper-parameters selection; and finally the study benchmarks imputation methods *and* predictive models that handles missing values. As a result, our benchmark is very computation-intensive: the whole study costed approximately 520 000 CPU hours, *i.e.* 60 years on a single CPU, revealing the need to also account for compute cost in recommendations.

After briefly exposing our benchmarking methodology, we give a synthetic view of the findings and discuss observed trends. Overall, the benchmarks reveal the presence of missing not at random (MNAR) values and non-linear mechanisms. High-quality conditional imputation gives good prediction provided that a variable indicating which entries were imputed is added to the completed data. However, its algorithmic complexity makes it prohibitively costly on large data. Rather, tree-based methods with integrated support for missing values (missing incorporated attribute – MIA) perform as well or better, at a fraction of the computational cost.

Empirical study

Benchmarking the imputation and MIA methods

Our experiments compare two-step procedures based on imputation followed by regression or classification, as well as tree-based models with an intrinsic support for missing values thanks to MIA. The ~~9~~ 12 methods compared are summarized in Table 1: MIA~~and~~, 8 methods based on ~~imputation~~single imputation and 3 methods using Multiple Imputation via Bagging. Below, we describe further the imputation strategies benchmarked as well as MIA.

Single Imputation

Constant imputation: mean and median. The simplest approach to imputation is to replace missing values by a constant such as the mean, the median or the mode of the corresponding feature. This is frowned upon in classical statistical practice, as the resulting data distribution is severely distorted compared to that of fully-observed data. Yet, in a supervised setting, the goal is different from that of inferential tasks. Recent theoretical results have established that powerful learners such as ones based on trees can learn to recognize such imputed values and give the best possible predictions (Josse et al.; 2019). The key to the success of this strategy is to impute the training and the test set with the same constant: missing values of the test set are imputed with the constants learned on the training set (mean, median, etc).

Conditional imputation: MICE and KNN. Powerful imputation approaches rely on conditional dependencies between features to fill in the missing values. Adapting machine-learning techniques gives flexible estimators of these dependencies. Classical approaches include k-nearest neighbor regressors (Chen and Shao; 2000), and iterative conditional imputers that predict one feature as a function of others, as with the MICE imputer (Buuren and Groothuis-Oudshoorn; 2010). In our experiments, we benchmark their implementation in scikit-learn (Pedregosa et al.; 2011): the `KNNImputer` as well as the `IterativeImputer`, using linear models to impute missing values.

Table 1. Methods compared in the main experiment.
All use gradient-boosted trees as predictive model. ~~8~~ 10 use imputation and ~~1~~ 2 uses MIA. Bagging uses 100 estimators in the ensemble.

4.2pt

In-article name	Imputer	Mask	Bagging	Predictive model
MIA	–	No	No	Boosted trees
Mean	Mean	No	No	Boosted trees
Mean+mask	Mean	Yes	No	Boosted trees
Median	Median	No	No	Boosted trees
Median+mask	Median	Yes	No	Boosted trees
Iterative	Iterative	No	No	Boosted trees
Iterative+mask	Iterative	Yes	No	Boosted trees
KNN	KNN	No	No	Boosted trees
KNN+mask	KNN	Yes	No	Boosted trees
Iterative +Bagging	Iterative	No	Yes (100)	Boosted trees
Iterative+mask +Bagging	Iterative	Yes	Yes (100)	Boosted trees
MIA+Bagging	–	No	Yes (100)	Boosted trees

Adding the mask. Conditional imputation can make it hard for the learner to retrieve which entries were originally observed and which were originally missing. However, the information of missingness can be relevant for predicting the outcome in cases where it depends on missingness, or in *missing not at random* settings where the missingness carries information. For these reasons, it can be useful after imputation to add new binary features that encode whether a value was originally missing or not: the *mask* or *missingness indicator* (Josse et al.; 2019; Sharafoddini et al.; 2019; Sperrin et al.; 2020).

Multiple Imputation

When estimating model parameters, it is of great importance to reflect the uncertainty due to the missing values. For this purpose, Multiple Imputation methods are widely used, often via Resampling methods such as the Bootstrap. However, for prediction (classification or regression) theoretical conditions differ from that of parameters estimation. Indeed, it has been shown recently that a sufficiently flexible learner reaches optimal performances asymptotically with Single Imputation, whatever the missing data mechanism and whatever the choice of imputation function (Le Morvan et al.; 2021). Still, this result holds in asymptotic regimes, and there is a need for empirical results on handling missing values with Multiple Imputation or Bootstrap in the context of supervised learning. Theoretically, the only result that we are aware of for Multiple Imputation in the context of prediction requires access to an oracle predictor for fully observed data and is valid only in MAR (Josse et al.; 2019, th. 3). In general, it is not clear how to use Multiple Imputation for supervised learning: sampling can be applied in different ways during training the model or applying their predictions to new data. Khan et al. (2019) review and compare a number of methods for using Multiple Imputation and Bootstrap: learning on an averaged version of a multiply imputed dataset, bagging single imputations, bagging Multiple Imputations, constructing ensembles based on predictors that were each learned on a version of a multiply imputed dataset (Friedman et al.; 2001, chap 16). As these methods all come with a significant computing cost, we focus on the most promising approach: bagging single imputation. More precisely, we draw for each task 100 bootstrap replicates. We then fit the single imputation and the predictive model on each of these replicates, to obtain 100 predictors. Final predictions are made either by voting or by averaging (see Supplementary Table 5).

Directly handling missing values with tree-based models: MIA

We also consider the MIA (Missing Incorporated in Attribute) strategy to readily model missing values in tree-based models. It has the benefit of using all samples, including incomplete ones, to produce the splits of the input space. More precisely for each split based on variable j, all samples with a missing value in variable j are either sent to the left or to the right child node depending on which option leads to the lowest risk. Note that the samples with an observed value in variable j can either be split between the left and right child node according to whether their values x_j is greater or smaller than a threshold~~z~~, or either all be sent to the same child node so that they are separated from the samples with a missing value in variable j. That makes MIA particularly suited to Missing Not At Random (MNAR) settings, as it can harness the missingness informa-

tion. Moreover, since trees with MIA directly learn with missing values, they provide a straightforward way of dealing with missing values in the test set. We use the implementation in scikit-learn's boosted trees (`HistGradientBoostingRegressor`).

Predictive model

For the supervised learning step, we focus on gradient-boosted trees –though we also benchmark linear models in a complementary analysis ~~exposed~~ described in the appendices. We applied supervised learning to the imputed data for the imputation-based methods. We also used the tree models with their support of MIA for a direct handling of missing values. Gradient-boosted trees are state-of-the art predictors for tabular data (Chen and Guestrin; 2016; Olson et al.; 2018; Shwartz-Ziv and Armon; 2021), and thus constitute a strong baseline. Moreover, using gradient-boosted trees enables us to keep the same predictive model for all approaches, thereby putting emphasis on the impact of the missing data treatment.

To define the input features we either use the choice of experts in prior studies, or feature screening, a classic machine-learning procedure using a simple ANOVA-based univariate ~~tests~~ test of the link of each feature to the outcome (Guyon and Elisseeff; 2003). ~~This step~~ In both cases, the same set of selected features is used for all methods within each predictive task. Selecting features is necessary because some of the imputation methods studied are not tractable with a large number of features.

Health databases

To reach conclusions as general as possible we used four real-world health-related databases. These databases vary in terms of location, size, purpose and time, to cover a wider data scope. These databases already existed and no data collection was made in this study. Below, we describe them briefly, giving the prediction tasks studied for each of them.

Traumabase

The Traumabase Group is a collaboration studying major trauma. The database gathers information from 20 French trauma centers on more than 20 000 trauma cases from admission until discharge from critical care. Data collection started in 2010 and is still ongoing in 2020. We used records spanning from 2010 to 2019. Data can be obtained by contacting the team on the Traumabase website (www.traumabase.eu).

We defined 5 prediction tasks on this database, 4 classifications and 1 regression. Outcomes are diverse: patient's death, hemorrhagic shock, septic shock, and platelet count. Features for the hemorrhagic shock prediction are taken from Jiang et al. (2020).

UK Biobank

UK Biobank (UKBB) (Sudlow et al.; 2015) is a major prospective epidemiology cohort with biomedical measurements. It provides health information on more than 500 000 United-Kingdom participants aged between 40 to 69 years from 2006 to 2010. The data are available upon application as detailed on the UK BioBank website (www.ukbiobank.ac.uk).

We defined 5 tasks on this database, 4 classifications and 1 regression. Outcomes are the diagnosis of three diseases - breast cancer, skin cancer, Parkinson's disease - as well as prediction of the fluid-intelligence score. Breast cancer prediction uses features defined in Läll et al. (2019).

MIMIC-III

The Medical Information Mart for Intensive Care (MIMIC) database (Johnson et al.; 2016) is an Intensive Care Unit (ICU) dataset developed by the MIT Lab for Computational Physiology. It comprises deidentified health data associated with about 60 000 ICU admissions recorded at the Beth Israel Deaconess Medical Center of Boston, United States, between 2001 and 2012. It includes demographics, vital signs, laboratory tests, medications, and more. The data can be accessed via an application described on the MIMIC website (mimic.physionet.org).

We defined 2 classification tasks on this database. Outcomes are septic shock and hemorrhagic shock.

NHIS

The National Health Interview Survey (NHIS) (National Center for Health Statistics; 2017) is a major data collection program of the National Center for Health Statistics (NCHS), part of the Centers for Disease Control and Prevention (CDC) in the United States. It aims to monitor the health of the population. Since 1957, it collects data from United-States population. We use the 2017 edition, summing up to approximately 35 000 households containing about 87 500 persons. The database is freely-accessible on the NHIS website (www.cdc.gov/nchs/nhis/).

We defined 1 regression task on this database. Outcome is the yearly income.

More details on each database and task can be found in the appendices, in particular in Supplementary Table 7 ~~, Supplementary Fig.~~ and Supplementary Figure 4 that detail the number of features available and their type (numerical, ordinal and categorical), and Supplementary ~~Fig.~~ Figure 6 giving the distribution of missing values across features.

Findings

~~Fig.~~ Figure 1 summarizes the performances and computational times of the various methods across the 4 databases and 13 prediction tasks. To explore the importance of the amount of data, we created ~~train~~ training datasets of 4 ~~different~~ sizes: 2 500, 10 000, 25 000 and 100 000 samples. We report the general trends.

~~MIA takes the lead~~ Bagging improves prediction, ~~although not significantly~~ MIA performs well at limited cost

Iterative+mask+Bagging obtains the best overall average rank (~~1.9~~ 2.6) across all tasks and ~~dataset~~ sizes in terms of prediction score closely followed by MIA+Bagging (2.8) as shown on ~~Fig.~~ Figure 1a and Supplementary Table 8b. ~~In further details, MIA is also the best performing method in terms of prediction scores on every size with average ranks of 1.9, 2.2, 2.4 and 1.2 on the sizes 2 500, 10~~ Overall, Bagging improves markedly all approaches (supplementary Figure 9). However the cost of these bagged methods can be prohibitive. At size n=100 000, ~~25 000 and~~ Iterative+mask+Bagging and MIA+Bagging cost 369 and 117 CPU days per task respectively, about 100 ~~000 respectivelyas shown on Supplementary Fig.~~ 3a~~.~~ to 200 times slower than non-bagged method such as MIA (1.9 CPU days per task).

MIA enables to navigate a trade off between prediction performance and computational tractability: With Bagging it comes close to Iterative+Mask with half the computational cost on large databases. Without Bagging, it is the best overall performer, with an overall average rank of 4.3, and up to 200 times faster. It is followed by Mean+mask, Median+mask and Iterative+mask ~~are the next 3 best methods~~ with overall average ranks of ~~2.8, 3.1 and 3.7~~ 5.2, 5.5 and 6.0 respectively. Mean, ~~Iterative,~~ KNN+mask, Iterative, Median and KNN ~~perform~~ performed the worst with overall average ranks of ~~5.0, 6.4, 6.4, 6.7 and 8.8 respectively. For~~ 7.5, 8.9, 9.0, 9.2 and

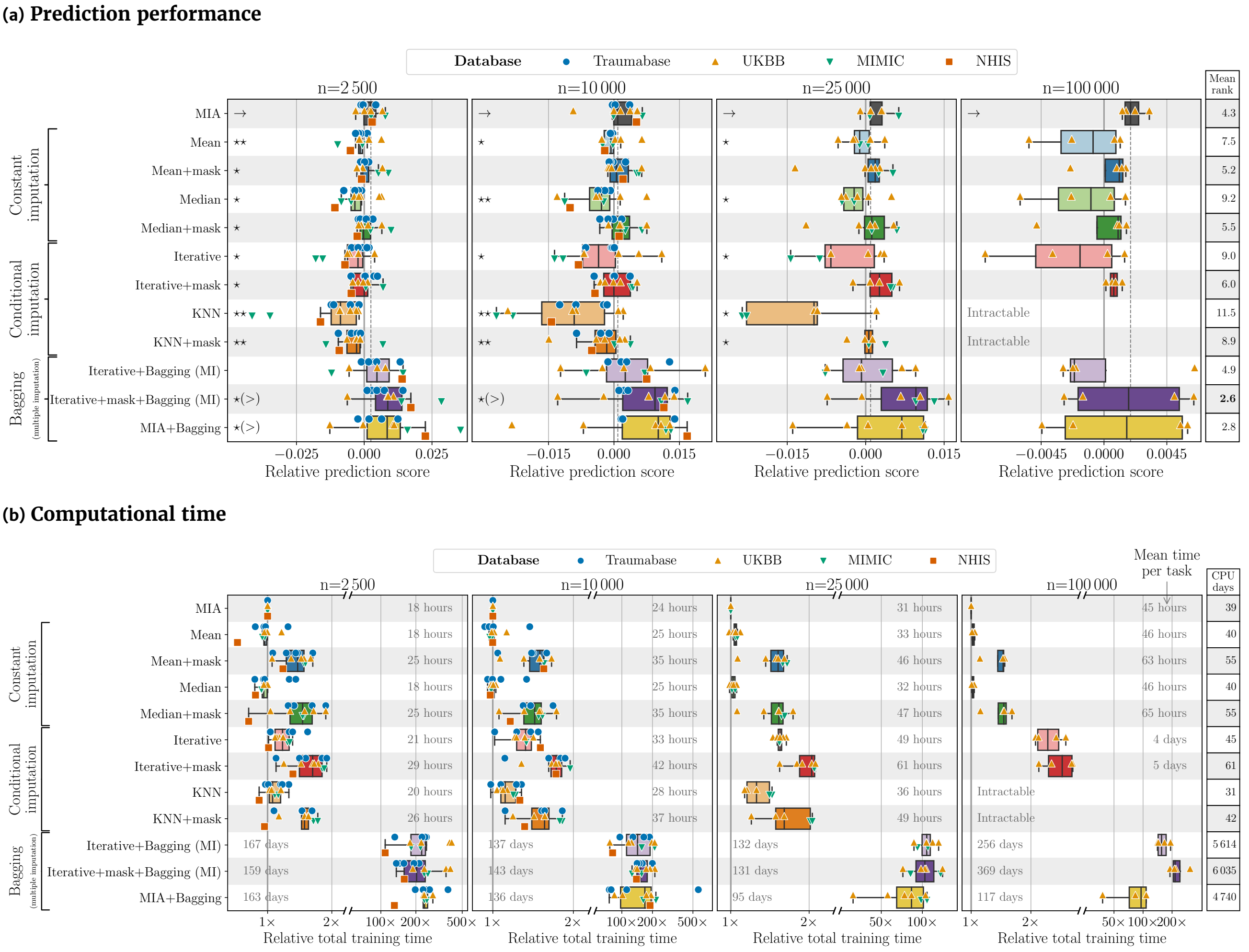

Figure 1. Gradient-boosted trees models. Comparison of prediction performance and training times across the ~~9~~ 12 methods (see Table 1) for 13 prediction tasks spread over 4 databases, and for 4 sizes of dataset (2 500, 10 000, 25 000 and 100 000 samples). For each of the tasks and sizes, we computed a reference score by averaging the scores obtained by the ~~9~~ 12 methods on the corresponding task and size. The relative prediction score of a method on a task and size is the deviation of the prediction score from the reference score of this task and size. For computational time, the total training time comprises imputation and tuning times and is given relative to the one of MIA for each task and size. More details on how these plots were created are given in the *Plotting method* section. The ~~table gives~~ significance is assessed with a one-sided Wilcoxon signed-rank test with MIA taken as reference (see Supplementary Table 4a). Methods which performed significantly poorer (resp. better) at the 0.05 level are marked with "⋆" (resp. "⋆(>)") and "⋆⋆" (resp. "⋆⋆(>)") for Bonferroni-corrected levels. Two tables give the overall average ranks ~~of~~ and the ~~methods~~ total number of CPU days for each method, all tasks and sizes combined. ~~More detailed~~ The average number of CPU hours *per task* required to evaluate each method is given on each line. Detailed scores and ranks broken out by tasks are given in Supplementary Table 8 and Supplementary ~~Table~~Figure 97. Notice that KNN and KNN+mask were intractable at $n = 100\,000$ due to their memory footprint of $\mathcal{O}(n^2)$.

11.5 respectively. Supplementary Table 8a and 8b give more quantitative details about scores and ranks of each method.

Similar observations can be made on each size separately. MIA obtained the best prediction scores on every size with average ranks of 4.3, 4.6, ~~refer to Supplementary Table~~ 8a ~~and~~ 8b4.4 and 2.5 on sizes 2 500, 10 000, 25 000 and 100 000 respectively as shown on Supplementary Figure 3a.

~~The better performance of MIA does not come at~~

In terms of computing time, beyond the fact that Bagging multiplies by 100 the cost of ~~a longer training time. Indeed, Fig.~~ 1b ~~shows that MIA is among the fastest procedures along~~ every method, MIA is almost always the fastest (Figure 1b), though it gives excellent prediction performance. It is on par with Mean, and Median imputations~~. Training with MIA took up to 33% less time than the average training time of all methods combined~~, but adding the mask to these methods –a key ingredient to prediction performance– doubles their computing times. At the other end of the spectrum, Iterative+mask and KNN+mask are the slowest non-bagged methods. The ~~gap between computational time increases~~ gaps between training times of the methods increase with the size of the database, revealing the difference in algorithmic scalability.

~~*Significance*~~*Statistical significance.* To assess significance of the above results, we ran three statistical tests: the Friedman test (Friedman; 1937, 1940), the Nemenyi test (Nemenyi; 1963) and the one-sided Wilcoxon signed-rank test (Wilcoxon; 1945), all described in Demšar (2006).
The Friedman test compares the average ranks of several algorithms ran on several datasets. The null hypothesis assumes that all algorithms are equivalent, ~~*i.e.*~~ *i.e.* their rank should be equal. Table 3a shows that the null hypothesis is rejected with p-values way below the 0.05 level for the ~~four dataset sizes .~~ sizes 2 500, 10 000 and 25 000. This indicates that at least one algorithm has significantly different performances from one other on these sizes. Following Demšar (2006), we then proceed with a post-hoc analysis with the Nemenyi test, assessing the significance of the difference between two algorithms using a critical ~~distance~~difference. Algorithms with a difference in ranks smaller than the critical ~~distance~~ difference are not significantly different. ~~Results show that MIA, Mean+mask,~~

~~Med+mask and Iter+mask always have ranks that do not significantly differ across all dataset sizes (~~Unfortunately, there are many methods to compare (12) comparatively to the number of datasets (13). As a result, the critical difference is high as shown in equation (3) and Supplementary Table 3a and ~~Supplementary Fig.~~ 3a~~). Hence our experiments do not give evidence that the lead of MIA over~~ there is almost no significance when comparing the performance of MIA with the one of the other methods ~~is significant. Results do show that MIAsignificantly outperform KNN~~as shown on Supplementary Figure 3a. However, there are some significance when comparing bagged methods. For example at size n=2 500, Iterative+mask+Bagging and MIA+Bagging performed significantly better than Mean, Median, Iterative, KNN+mask ~~, Iterative and Median on nearly all sizes (Supplementary Fig.~~ 3a~~).~~ and KNN.

~~A~~ We run a complementary analysis with a one-sided Wilcoxon signed-rank test, used for non-parametric tests comparing algorithms pairwise~~leads to the same conclusions as the Nemeyni test (~~. We compare MIA to every other methods. The null hypothesis claims that the median of the score differences between the two methods is positive (resp. negative) for the one-sided right (resp. one-sided left) test. Results of the test is shown on Figure 1a and Supplementary Table ~~4).~~ 4b. At size n=2 500, MIA performed significantly better than every other non-bagged methods at the 0.05 level. MIA also performed significantly better than Mean, KNN and KNN+mask at the Bonferroni-corrected level. Bagged methods Iterative+mask+Bagging and MIA+Bagging performed significantly better than MIA at the 0.05 level. The bigger the size n, the less tasks are available and so the less significant are the results.

Adding the mask improves prediction

Imputations with the additional variable representing the mask perform systematically better in terms of average prediction score than their counterpart without mask ~~, and the difference is significant~~ (Figure 1a, Supplementary Table 8b).

In addition, MIA is not significantly better than the masked imputations yet it is for the non-masked imputations (~~Supplementary Table~~ 4~~, Supplementary Fig.~~ 3aFigure 1a, Supplementary Table 4). However, adding the mask leads to longer training times (~~Fig.~~Figure 1b). Indeed, adding the mask doubles the number of features for the supervised-learning step.

Conditional imputation is on par with constant imputation

~~Fig.~~Figure 1 shows that conditional imputation using Iterative or KNN imputers does not perform consistently better than constant imputation. The overall mean rank of Iterative and KNN are ~~6.4 and 8.8 versus 5 and 6.7~~ 9.0 and 11.5 versus 7.5 and 9.2 for Mean and Median respectively (~~Table~~ Figure 1a and Supplementary Table 8b), and a similar delta is visible on the masked version.

Supplementary finding: Boosted-trees outperform linear methods

Imputation methods paired with a linear model performed poorer than when paired with boosted-trees (Supplementary ~~Fig.~~Figure 2, Supplementary Table 9b). Additionally, boosted-trees paired with MIA are significantly better than every other method based on a linear model (Supplementary Table 4).

Discussion

Interpretation

~~Good imputation does not imply~~ Model aggregation drives the good ~~prediction~~performance of Multiple Imputation

As with standard multiple-imputation strategies used for parameter estimation, Bagging generates multiple bootstrap replicate training sets. Yet, the standard practice of Multiple Imputation strives to capture well the conditional distribution of the missing values given the observed one, while such conditional imputation is not needed for good prediction (as revealed by the good performance of MIA and Le Morvan et al.; 2021). Indeed, Bagging in itself is known to improve generalization. To answer whether the good performance of Multiple Imputation can be attributed to ensembling –averaging multiple predictors– or capturing the conditional distribution, we performed additional experiment with Mean+mask+Bagging (see Supplementary Figure 9). We observed that Mean+mask+Bagging is on par with Iterative+mask+Bagging, which suggests that the improved performances are rather due to the effect of Bagging itself rather than capturing the conditional distribution of the missing data given the observed ones.

Good imputation does not imply good prediction, even for Multiple Imputation

It may be surprising at first that a sophisticated conditional imputation does not outperform constant imputation. Indeed, it contradicts the intuition that better imputation should lead to better prediction. Theoretical work shows that this intuition is not always true (Le Morvan et al.; 2021): even in MAR settings, it may not hold for strongly non-linear ~~mechanism~~ mechanisms and little dependency across features. In the health databases that we studied, the features are weakly correlated: on average, only 12% of the features are correlated at more than 0.3 in absolute value (Supplementary Table 6). This low correlation among features may ~~exlain~~ explain our findings. If features are mostly independent, there is little information on the unobserved values to be extracted from the observed ones. For supervised learning, constant imputation comes with the benefit that it creates a simple structure captured by the supervised-learning step, which can then adapt to the missingness (Josse et al.; 2019).

~~The missingness is informative~~

~~For imputation-based pipelines, prediction significantly improves with the missingness mask added as input features. This suggests that the missingness is informative, which is often the case in health databases (Agniel et al.; 2018; Madden et al.; 2016). Hence for all health databases studied, either the covariates are Missing Not At Random (MNAR) or the outcome to predict depends on the missingness. Either cases fall outside of the theoretical framework that grounds the validity of statistical analysis using imputation (Rubin; 1976; Josse et al.; 2019). The empirical results also confirm that the practice of adding the mask as input allows to harness the predictive information in missing data patterns (Sperrin et al.; 2020), otherwise hidden in the imputed data and much more difficult to recover.~~

Boosted-trees with MIA give best predictive models at little cost

MIA, the missing-values support inside gradient-boosted trees, appears as a method of choice to deal with missing values. It was on average the best performing one in terms of performance in our extensive benchmark while having a low computational cost. Sophisticated conditional imputation such as the Iterative or KNN imputers are appealing because they may recover plausible values for missing entries, as discussed below. However, they are intractable with large datasets. Beyond the costs outlined by our experiments (~~Fig.~~Figure 1b), the

broader problem is the algorithmic scalability: for a dataset of p features and n samples, the compute cost of a KNN imputer scales as $n^2 p^2$ and the memory footprint as n^2, while the compute cost of an iterator imputer scales as $p^2 n \min(n, p)$ when it is based on linear models, the cheapest alternative. If both p and n grow, these costs rapidly becomes prohibitive. They prevented us from exploring larger datasets, ~~*eg*~~ *e.g.* with more features. Note that to ground valid predictions, the imputation model must be learned only on the train set; hence it is recomputed many times in a cross-validation loop.

Regardless of missing-values handling, gradient-boosted trees predict significantly better than linear models (Supplementary Table 4b). Tree-based models excel on categorical or ordinal features, however these are only a minority of the features of the databases studied (Supplementary ~~Fig.~~Figure 4). Hence the good performance of gradient-boosted trees probably reveals non-linear mechanisms in the data. Note that the smallest database that we explored has a sample size of ~~n = 2 500.~~ n=2 500. For much smaller data, the simplest model –the linear model– may be the best choice.

The missingness is informative

For imputation-based pipelines, prediction significantly improves with the missingness mask added as input features. This suggests that the missingness is informative, which is often the case in health databases (Agniel et al.; 2018; Madden et al.; 2016). Hence for all health databases studied, either the covariates are Missing Not At Random (MNAR) or the outcome to predict depends on the missingness. Either cases fall outside of the theoretical framework that grounds the validity of statistical analysis using imputation (Rubin; 1976; Josse et al.; 2019). The empirical results also confirm that the practice of adding the mask as input allows to harness the predictive information in missing data patterns (Sperrin et al.; 2020), otherwise hidden in the imputed data and much more difficult to recover.

Features with high missing rates are also important.

Within each task, the missing rate per feature varies over a wide spectrum (see Supplementary Figure 6). We checked that features' missing rates and predictive importance were not associated. For this, we measured permutation features: the drop in a model score after shuffling a feature, thereby cancelling its contribution to the model performance. We ran this experiment for each task and each feature using scikit-learn's implementation (see Supplementary Table 5). We found no association between a feature's missing rate and its importance (Supplementary Figure 8). Predictions do not only rely on features with few missing values. Moreover, even features with a very high level of missing values (for example > 80%) seem to be as important as the others. This highlights the fact that it is worth making the effort of learning with incomplete features, even when they have a high missing rate.

Imputation may benefit robustness or interpretability

A good imputation may bring the benefit of recovering a meaningful missing value, reflecting a biological or clinical reality rather than operational constraints. For instance, the weight of a patient may be measured upon scheduled admission to a hospital but not at the emergency department. A predictive model based on an imputed underlying value may lend itself better to mechanistic interpretation than a model implicitly capturing missingness such as MIA. In addition, using missingness to drive prediction may be more fragile, ~~*eg*~~ *e.g.* to changes in the operational process. In such a case, shifts in the missing data patterns should be closely monitored (Sperrin et al.; 2020; van Smeden et al.; 2020; Groenwold; 2020) as they could seriously alter prediction performance. Indeed, machine-learning models building their predictions on "shortcuts" in the data –not directly related to outcome of interest but rather to the acquisition– sometimes generalize less well to new hospitals (DeGrave et al.; 2021). Nevertheless, in health the mere presence of a measure, such as a colonoscopy, is often an indication in itself.

Limitations and further work

Limitations: not ~~every difference is significance~~all differences are significant

Relative performance of approaches varies across datasets, which is not surprising as no prediction model is expected to dominate on all data. The diversity of the datasets and the statistical analysis grounds the generality of the findings. Yet, not all differences are significant at large sample sizes. This lack of significance can simply be explained because of a small statistical power of the benchmark as only a few datasets are available to test these very large sample sizes settings (only 4 tasks at the ~~n = 100 000~~ n=100 000 size).

More datasets would probably have made more differences significant. Yet, the benchmarks presented here already incurred large computational costs, due to the nested cross-validation: about ~~12~~520 000 CPU hours. Also, the findings build upon 13 different tasks, markedly more than the typical machine-learning benchmark: only 6% of empirical results published at NeurIPS and 8% ICLR (both leading machine-learning venues) build upon more than 10 datasets (Bouthillier and Varoquaux; 2020).

Limitations: imputation quality is not assessed

All the conclusions of this study pertain to prediction and do not allow us to conclude on imputation's ability to accurately reconstruct missing values. The focus of our study is indeed on prediction.

Further work: more benchmarks would be interesting, and costly

To limit computation costs and mimic typical usage, no hyper-parameters tuning was performed on the parameters of the imputers. Recently, software tools have been introduced to perform model selection on imputation jointly with the supervised step (Jarrett et al.; 2021; Borowski and Fic; 2021). Further evaluation could quantify how much gains are brought by such joint model selection, though it would need sizable computational resources.

Further work could test more supervised learning models. The motivation of the present study was not to find the absolute best pipeline, but rather to understand compromises that hold across datasets and are readily usable.

Conclusion

Extensive benchmarking on health databases reveals trends in the performance of methods to build predictive models handling missing values. First, directly incorporating missing values in tree-based models with MIA, gives a small but systematic improvement in prediction performance over prior imputation. Second, the computational cost of imputation using MICE or KNN becomes intractable for large datasets. Third, gradient-boosted trees give better predictions than linear models. Fourth, Bagging increases predictive performance but with a severe computational cost. Fifth, good imputation does not imply good prediction as both have different tradeoffs. Finally, the experiments reveal that the missingness is informative. ~~Hence,~~ Overall, a novel message of this benchmark is that for

building predictive models, supervised learning directly handling missing values should be considered, beyond imputation.

Potential implications

This work suggests a departure from current practices: supervised learning directly handling missing values can be preferable to imputation. In particular, classic conditional expectation methods can be computationally intractable both in terms of time and memory on large datasets. Constant imputation with the mask also performs well with little costs.

Detailed benchmarking methodology

Experiment

We selected four real databases with missing values described in *Health databases*. From them we defined empirically 13 prediction tasks – that is a set of input features and an outcome to predict – with the intent of covering as diverse use cases as possible: regressions, classifications, diverse outcomes, diverse feature types (numerical, ordinal and categorical). We sub-sampled the datasets to study 4 sizes: 2 500, 10 000, 25 000 and 100 000 samples. We selected a subset of features from the databases for each prediction task using two approaches. Manually selecting or defining features based on articles or automatically selecting 100 encoded features using an univariate ANOVA selection. We often used the later because it has the advantage of not requiring expert knowledge to define the features. Manual selection keeps fewer features than our automated selection. Note that we one-hot encoded categorical features before selecting 100 encoded features with ANOVA. Less than 100 non-encoded features may thus be involved in the task. The ANOVA is fitted on one third of the samples and the two remaining thirds are kept for fitting and evaluating the methods. To reduce bias induced by the choice of subset on which is fit the ANOVA, we ran 5 trials in which the subset is each time redrawn and average the ~~results~~scores and times. Task having their features manually selected are given the whole samples and only 1 trial is performed. Each of the 12 methods is given the exact same features and cross-validation folds.

The next step consists in benchmarking the ~~9~~ 12 methods of Table 1 on the defined prediction tasks. We used the implementation from scikit-learn (Pedregosa et al.; 2011) for all methods (see Supplementary Table 5). Two nested cross-validations are used. ~~The inner one performs~~ The outer one yields 5 training and test sets. On each training set, we perform a cross-validated hyper-parameter ~~tuning on the predictive model to make the results less dependant of the model's parameters. The outer one averages the validations of the tuned model . We then assessed~~ search –the inner cross-validation– and select the best hyper-parameters. We evaluate the best model on the respective test set. We assess the quality of the prediction with a coefficient of determination for regressions and the area under the ROC curve for classification. We average the scores obtained on the 5 test sets of the outer cross-validation to give the final score. Finally, we ~~compared~~ compare averaged prediction scores one to each other.

~~Training~~ We also monitored training and imputation times ~~were also monitored~~ to add time concerns to our analysis. A very detailed description of the experimental method is available on protocols.io. A link to the protocol and to the code of the experiments is given in *Availability of supporting data and materials* and *Availability of source code and requirements*.

Plotting method

Figures on prediction scores

The experiment gives one prediction score per fold, per trial, per task, per method, per size. Cross-validations aggregate and average scores across the folds and trials resulting in an average score for each of the (task, method, size). For each one of the pairs (task, size), we computed a reference score by averaging the scores obtained by the ~~9~~ 12 methods on the corresponding task and size. The plotted metric is what we called the relative prediction score – that is the deviation of the prediction score from the reference score – for each of the (task, method, size). We created one box plot for each of the 4 sizes with the same structure: the relative prediction score on the x-axis and the ~~9~~ 12 methods on the y-axis. Each is overlaid with a scatter plot plotting the relative prediction score per (task, method, size). The scatter plot shares its x-axis with the box plot. On the y-axis however, each dot is given a y coordinate according to its method and database so that scores coming from a same method and database are plotted on the same ~~line. Aggregated scores having any missing underlying score are grayed out and excluded from the box plot computation. For example, sometimes a prediction failed on a particular fold on a particular trial (*eg* due to convergence error). In this case, some scores are missing to fully compute the average. We want to exclude these incomplete averages from the benchmark to avoid distorting the results.~~ horizontal line.

Figures on computation time

Computational time plots follow the same structure. The metric of interest is now the total training time. It includes imputation time and the full hyper-parameters tuning time. It is evaluated using computer's process time instead of wall-clock time. The ~~reference value is computed in the same way as with prediction scores, that is by averaging the times across the 9 methods~~ total training time of MIA is taken as reference time for each (task, size). ~~However the~~ The relative total training time is computed by dividing by the reference ~~value. Indeed, the~~ time. The x-scale is ~~now~~ logarithmic to better apprehend comparison on large scales.

Availability of source code and requirements

- Project name: Benchmarking missing-values approaches for predictive models on health databases.
- Project home page: https://github.com/alexprz/article-benchmark_mv_approaches
- Operating system: Platform independent
- Programming language: Python 3.7.6
- Other requirements: all requirements are listed in the requirements.txt file of the repository.
- License: MIT

Availability of supporting data and materials

The datasets supporting the results of this article are available at the following URL.

- **Traumabase**, by contacting the team at http://www.traumabase.eu/en_US/contact.
- **UKBB**: upon application at https://www.ukbiobank.ac.uk/register-apply/.
- **MIMIC-III**: upon application at https://mimic.physionet.org/gettingstarted/access/.
- **NHIS** freely available at https://www.cdc.gov/nchs/nhis/nhis_2017_data_release.htm.

A thorough description of the protocols of the experiments conducted in this article is available on protocols.io at: https://www.protocols.io/view/benchmarking-missing-values-approaches-for-predict-bmvgk63w.

Declarations

List of abbreviations

The following abbreviations were used in this article:

- ANOVA: Analysis Of Variance.
- AUC: Area Under the Curve.
- KNN: K-Nearest Neighbors.
- MAR: Missing At Random
- MIA: Missing Incorporated in Attribute.
- MNAR: Missing Not At Random
- MIMIC: Medical Information Mart for Intensive Care.
- NHIS: National Health Interview Survey.
- LOWESS : Locally Weighted Scatterplot Smoothing.
- UKBB: UK Biobank.

Ethical Approval

Only secondary data use was involved in this work. Each one of the databases has previously been approved by the Ethical Review Board. Access to each database was obtained according to the corresponding rules and authorization, when applicable (MIMIC, Traumabase, UKBB).

Consent for publication

Not applicable.

Competing Interests

The authors declare that they have no competing interests.

Funding

Gaël Varoquaux, Julie Josse, and Marine Le Morvan acknowledge funding from DataAI under the MissingBigData grant. Gaël Varoquaux and Marine Le Morvan acknowledge funding from ANR under the DirtyData grant (ANR-17-CE23-0018). Alexandre Perez-Lebel was partially funded by a Mitacs Globalink Research Award for research in Canada. ~~JB~~ Jean-Baptiste Poline is partially funded by the National Institutes of Health (NIH) NIH-NIBIB P41 EB019936 (ReproNim) NIH-NIMH R01 MH083320 (CANDIShare) and NIH RF1 MH120021 (NIDM), the National Institute Of Mental Health of the NIH under Award Number R01MH096906 (Neurosynth), as well as the Canada First Research Excellence Fund, awarded to McGill University for the Healthy Brains for Healthy Lives initiative and the Brain Canada Foundation with support from Health Canada.

Author's Contributions

Following the CRediT Contributor Roles Taxonomy:

- Alexandre Perez-Lebel: Investigation, Data curation, Formal Analysis, Writing – original draft, Writing – review & editing.
- Gaël Varoquaux: Funding acquisition, Conceptualization, Supervision, Writing – review & editing, Validation.
- Marine Le Morvan: Supervision, Writing – review & editing, Validation.
- Julie Josse: Writing – review & editing.
- Jean-Baptiste Poline: Funding acquisition, Supervision, Writing – review & editing, Validation.

Acknowledgements

Not applicable.

Authors' information

Alexandre Perez-Lebel is an engineering student specialized in applied mathematics, computer science and machine learning. He interned one year at the Montreal Neurological Institute of McGill University in Montreal and is ~~now finishing his master's at Inria, before transiting to~~ currently pursuing a PhD program in machine learning at Inria.

References

Agniel, D., Kohane, I. S. and Weber, G. M. (2018). Biases in electronic health record data due to processes within the healthcare system: retrospective observational study, *Bmj* **361**.

Bertsimas, D., Pawlowski, C. and Zhuo, Y. D. (2018). From Predictive Methods to Missing Data Imputation: An Optimization Approach, *Journal of Machine Learning Research* **18**(196): 1–39.
URL: *http://jmlr.org/papers/v18/17-073.html*

Borowski, J. and Fic, P. (2021). NADIA r package, https://cran.r-project.org/web/packages/NADIA/index.html. Accessed: 2021-05-26.

Bouthillier, X. and Varoquaux, G. (2020). Survey of machine-learning experimental methods at NeurIPS2019 and ICLR2020, *Research report*, Inria Saclay Ile de France.
URL: *https://hal.archives-ouvertes.fr/hal-02447823*

Buuren, S. v. and Groothuis-Oudshoorn, K. (2010). mice: Multivariate imputation by chained equations in r, *Journal of statistical software* pp. 1–68.

Chen, J. and Shao, J. (2000). Nearest neighbor imputation for survey data, *Journal of official statistics* **16**(2): 113.

Chen, T. and Guestrin, C. (2016). Xgboost: A scalable tree boosting system, *Proceedings of the 22nd acm sigkdd international conference on knowledge discovery and data mining*, pp. 785–794.

DeGrave, A. J., Janizek, J. D. and Lee, S.-I. (2021). Ai for radiographic covid-19 detection selects shortcuts over signal, *Nature Machine Intelligence* pp. 1–10.

Dempster, A. P., Laird, N. M. and Rubin, D. B. (1977). Maximum Likelihood from Incomplete Data via the EM Algorithm, *Journal of the Royal Statistical Society. Series B (Methodological)* **39**(1): 1–38.
URL: *http://www.jstor.org/stable/2984875*

Demšar, J. (2006). Statistical Comparisons of Classifiers over Multiple Data Sets, *Technical Report 1*.
URL: *http://jmlr.org/papers/v7/demsar06a.html*

Friedman, J., Hastie, T. and Tibshirani, R. (2001). *The elements of statistical learning*, Vol. 1, Springer series in statistics New York.

Friedman, M. (1937). The Use of Ranks to Avoid the Assumption of Normality Implicit in the Analysis of Variance, *Journal of the American Statistical Association* **32**(200): 675–701.

URL: https://www.tandfonline.com/doi/abs/10.1080/01621459.1937.10503522

Friedman, M. (1940). A Comparison of Alternative Tests of Significance for the Problem of *m* Rankings, *The Annals of Mathematical Statistics* **11**(1): 86–92.
URL: https://doi.org/10.1214/aoms/1177731944

Groenwold, R. H. (2020). Informative missingness in electronic health record systems: the curse of knowing, *Diagnostic and prognostic research* **4**(1): 1–6.

Guyon, I. and Elisseeff, A. (2003). An introduction to variable and feature selection, *Journal of machine learning research* **3**: 1157.

Iman, R. L. and Davenport, J. M. (1980). Approximations of the critical region of the fbietkan statistic, *Communications in Statistics - Theory and Methods* **9**(6): 571–595.
URL: https://doi.org/10.1080/03610928008827904

Jäger, S., Allhorn, A. and Bießmann, F. (2021). A Benchmark for Data Imputation Methods, *Frontiers in Big Data* **0**: 48.

Jarrett, D., Yoon, J., Bica, I., Qian, Z., Ercole, A. and van der Schaar, M. (2021). Clairvoyance: A pipeline toolkit for medical time series, *International Conference on Learning Representations*.

Jiang, W., Josse, J. and Lavielle, M. (2020). Logistic regression with missing covariates—Parameter estimation, model selection and prediction within a joint-modeling framework, *Computational Statistics and Data Analysis* **145**: 106907.

Johnson, A. E., Pollard, T. J., Shen, L., Lehman, L. W. H., Feng, M., Ghassemi, M., Moody, B., Szolovits, P., Anthony Celi, L. and Mark, R. G. (2016). MIMIC-III, a freely accessible critical care database, *Scientific Data* **3**(1): 1–9.
URL: www.nature.com/sdata/

Josse, J., Prost, N., Scornet, E. and Varoquaux, G. (2019). On the consistency of supervised learning with missing values, *arXiv preprint arXiv:1902.06931* .

Khan, S. S., Ahmad, A. and Mihailidis, A. (2019). Bootstrapping and multiple imputation ensemble approaches for missing data.

Läll, K., Lepamets, M., Palover, M., Esko, T., Metspalu, A., Tõnisson, N., Padrik, P., Mägi, R. and Fischer, K. (2019). Polygenic prediction of breast cancer: Comparison of genetic predictors and implications for risk stratification, *BMC Cancer* **19**(1).
URL: https://pubmed.ncbi.nlm.nih.gov/31182048/

Le Morvan, M., Josse, J., Scornet, E. and Varoquaux, G. (2021). What's a good imputation to predict with missing values?, *NeurIPS* .

Le Morvan, M., Prost, N., Josse, J., Scornet, E. and Varoquaux, G. (2020). Linear predictor on linearly-generated data with missing values: non consistency and solutions, *in* S. Chiappa and R. Calandra (eds), *Proceedings of the Twenty Third International Conference on Artificial Intelligence and Statistics*, Vol. 108 of *Proceedings of Machine Learning Research*, PMLR, pp. 3165–3174.
URL: http://proceedings.mlr.press/v108/morvan20a.html

Little, R. J. and Rubin, D. B. (2019). *Statistical analysis with missing data*, Vol. 793, John Wiley & Sons.

Madden, J. M., Lakoma, M. D., Rusinak, D., Lu, C. Y. and Soumerai, S. B. (2016). Missing clinical and behavioral health data in a large electronic health record (ehr) system, *Journal of the American Medical Informatics Association* **23**(6): 1143–1149.

Miotto, R., Li, L., Kidd, B. A. and Dudley, J. T. (2016). Deep patient: an unsupervised representation to predict the future of patients from the electronic health records, *Scientific reports* **6**(1): 1–10.

National Center for Health Statistics (2017). National Health Interview Survey (NHIS).
URL: https://www.cdc.gov/nchs/nhis/nhis_2017_data_release.htm

Nemenyi, P. (1963). *Distribution-free Multiple Comparisons*, Princeton University.
URL: https://books.google.fr/books?id=nhDMtgAACAAJ

Olson, R. S., Cava, W. L., Mustahsan, Z., Varik, A. and Moore, J. H. (2018). Data-driven advice for applying machine learning to bioinformatics problems, *PACIFIC SYMPOSIUM ON BIOCOMPUTING 2018: Proceedings of the Pacific Symposium*, World Scientific, pp. 192–203.

Pedregosa, F., Varoquaux, G., Gramfort, A., Michel, V., Thirion, B., Grisel, O., Blondel, M., Prettenhofer, P., Weiss, R., Dubourg, V. et al. (2011). Scikit-learn: Machine learning in python, *the Journal of machine Learning research* **12**: 2825.

Poulos, J. and Valle, R. (2018). Missing Data Imputation for Supervised Learning, *Applied Artificial Intelligence* **32**(2): 186–196.
URL: https://www.tandfonline.com/doi/abs/10.1080/08839514.2018.1448143

Rose, S. and Rizopoulos, D. (2020). Machine learning for causal inference in biostatistics, *Biostatistics* **21**(2): 336–338.

Rubin, D. B. (1976). Inference and missing data, *Biometrika* **63**(3): 581–592.
URL: https://academic.oup.com/biomet/article/63/3/581/270932

Sharafoddini, A., Dubin, J. A., Maslove, D. M. and Lee, J. (2019). A New Insight Into Missing Data in Intensive Care Unit Patient Profiles: Observational Study., *JMIR medical informatics* **7**(1): e11605.

Shwartz-Ziv, R. and Armon, A. (2021). Tabular data: Deep learning is not all you need, *arXiv preprint arXiv:2106.03253* .

Sperrin, M., Martin, G. P., Sisk, R. and Peek, N. (2020). Missing data should be handled differently for prediction than for description or causal explanation.

Steele, A. J., Denaxas, S. C., Shah, A. D., Hemingway, H. and Luscombe, N. M. (2018). Machine learning models in electronic health records can outperform conventional survival models for predicting patient mortality in coronary artery disease, *PloS one* **13**(8): e0202344.

Sudlow, C., Gallacher, J., Allen, N., Beral, V., Burton, P., Danesh, J., Downey, P., Elliott, P., Green, J., Landray, M., Liu, B., Matthews, P., Ong, G., Pell, J., Silman, A., Young, A., Sprosen, T., Peakman, T. and Collins, R. (2015). UK Biobank: An Open Access Resource for Identifying the Causes of a Wide Range of Complex Diseases of Middle and Old Age, *PLoS Medicine* **12**(3).
URL: https://pubmed.ncbi.nlm.nih.gov/25826379/

Twala, B. E. T. H., Jones, M. C. and Hand, D. J. (2008). Good methods for coping with missing data in decision trees, *Pattern Recogn. Lett.* **29**: 950–956.

Van Buuren, S. (2018). *Flexible imputation of missing data*, CRC press.

van Smeden, M., Groenwold, R. H. and Moons, K. G. (2020). A cautionary note on the use of the missing indicator method for handling missing data in prediction research, *Journal of clinical epidemiology* **125**: 188–190.

Wells, B. J., Chagin, K. M., Nowacki, A. S. and Kattan, M. W. (2013). Strategies for handling missing data in electronic health record derived data, *Egems* **1**(3).

Wilcoxon, F. (1945). Individual Comparisons by Ranking Methods, *Biometrics Bulletin* **1**(6): 80–83.
URL: http://www.jstor.org/stable/3001968

Zheng, T., Xie, W., Xu, L., He, X., Zhang, Y., You, M., Yang, G. and Chen, Y. (2017). A machine learning-based framework to identify type 2 diabetes through electronic health records, *International journal of medical informatics* **97**: 120–127.

(a) Prediction performance

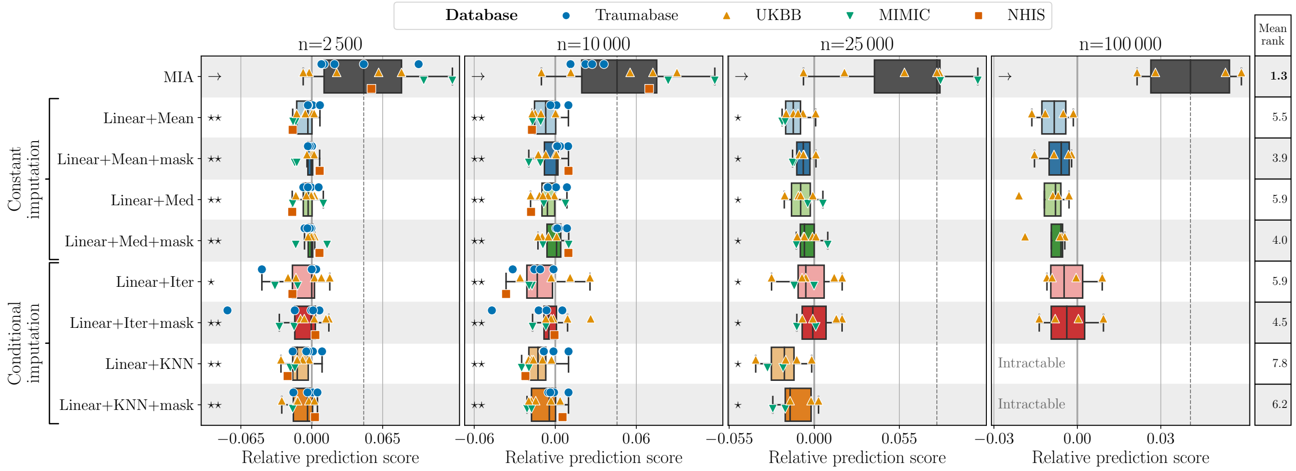

(b) Computational time

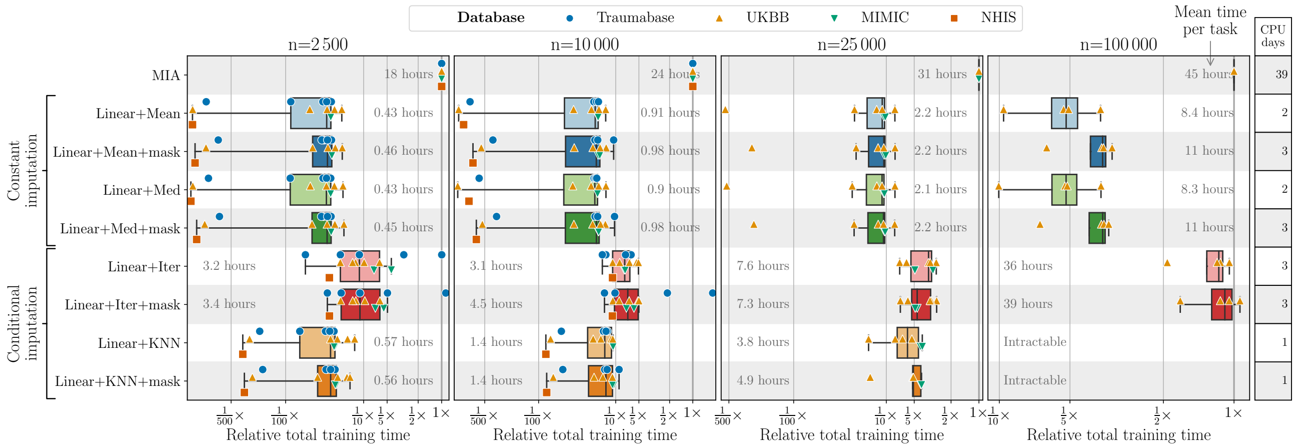

Figure 2. Supplementary results: linear and gradient-boosted trees models. Comparison of prediction performance and training times across the 9 methods (linear models and gradient boosting trees, see Supplementary Table 2) for 13 prediction tasks spread over 4 databases, and for 4 sizes of dataset (2 500, 10 000, 25 000 and 100 000 samples). For linear models, ridge is used for regressions and logistic regression for classifications. For each of the tasks and sizes, we computed a reference score by averaging the scores obtained by the 9 methods on the corresponding task and size. The relative prediction score of a method on a task and size is the deviation of the prediction score from the reference score of this task and size. For computational time, the total training time comprises imputation and tuning times and is given relative to the one of MIA for each task and size. More details on how these plots were created are given in the *Detailed benchmarking methodology* section. The significance is assessed with a one-sided Wilcoxon signed-rank test with MIA taken as reference (see Supplementary Table 4b). Methods which performed significantly poorer (resp. better) at the 0.05 level are marked with "⋆" (resp. "⋆(>)") and "⋆⋆" (resp. "⋆⋆(>)") for Bonferroni-corrected levels. Two tables give the overall average ranks and the total number of CPU days for each method, all tasks and sizes combined. The average number of CPU hours *per task* required to evaluate each method is given on each line. Detailed scores and ranks broken out by tasks are given in Supplementary Table 9. Notice that KNN and KNN+mask were intractable at n=100 000 due to their memory footprint of $\mathcal{O}(n^2)$.

Appendix

Supplementary experiment: linear models or trees?

Protocol

This supplementary experiment uses the same pipeline as the main experiment except that imputation is paired with linear models instead of boosted trees as summarized in Supplementary Table 2. We used ridge for regressions, and ℓ_2-penalized logistic regression for classifications.

Findings: trees with MIA improve upon linear models

MIA with boosted trees outperforms all 8 combinations of imputers with linear models, on every size and every database. Supplementary Figure 2a shows that MIA obtained the best average rank of 1.3 far ahead of other methods. The following ones are Linear+Mean+mask, Linear+Med+mask and Linear+Iter+mask with a rank of 3.9, 4.0 and 4.5 respectively. The one-sided Wilcoxon signed-rank test confirms this claim. Supplementary Table 4 shows that MIA with boosted trees is significantly better than every linear methods on the first two sizes even at the Bonferroni-corrected level. The null hypothesis of the Friedman test is rejected below the 0.05 level except for the last size as shown on Supplementary Table 3b. Thus methods are not equivalent for the first three sizes. The Nemenyi test on Supplementary Figure 3b confirms that results are not significant for the larger size.

Moreover, we were expecting the mean and median imputations to give bad results being paired with linear models as shown in Le Morvan et al. (2020). Not only these results confirm our expectations, but they also show that non-constant imputation models give similar results when paired with a linear model. As before, masked versions perform slightly better than their no-mask counterpart.

However, gradient-boosted trees with MIA are a lot slower than imputation with linear models. Supplementary Figure 2b shows that boosted trees with MIA is up to 500 times slower than constant imputations with linear models. Also, condi-

Table 2. Methods compared in the supplementary experiment.

In-article name	Imputer	Mask	Predictive model
Boosted trees+MIA	-	-	Boosted trees
Linear+Mean	Mean	No	Ridge/Logit
Linear+Mean+mask	Mean	Yes	Ridge/Logit
Linear+Med	Median	No	Ridge/Logit
Linear+Med+mask	Median	Yes	Ridge/Logit
Linear+Iter	Iterative	No	Ridge/Logit
Linear+Iter+mask	Iterative	Yes	Ridge/Logit
Linear+KNN	KNN	No	Ridge/Logit
Linear+KNN+mask	KNN	Yes	Ridge/Logit

tional imputation leads to slower computations than mean and median imputation. Given the low gain obtained against mean and median imputation, they are of limited interest.
The main takeaway is the outperformance in score of MIA with gradient-boosted trees over imputation with linear models when it comes to handling missing values. This outperformance comes with a cost: a much longer computation time.

Significance tests

In the following paragraphs, we took the notations and formulations of Demšar (2006). We consider k algorithms and N datasets. We note r_i^j the rank of the j-th algorithm on the i-th dataset. Note $R_j = \frac{1}{N}\sum_i r_i^j$ the average rank.

Friedman test. The Friedman statistic χ_F^2 is distributed according to a chi-square distribution with $k-1$ degrees of freedom.

$$\chi_F^2 = \frac{12N}{k(k+1)}\left[\sum_j R_j^2 - \frac{k(k+1)^2}{4}\right] \tag{1}$$

Iman and Davenport (1980) derived a less conservative statistic F_F which is distributed according to the F-distribution with $k-1$ and $(k-1)(N-1)$ degrees of freedom.

$$F_F = \frac{(N-1)\chi_F^2}{N(k-1)-\chi_F^2} \tag{2}$$

Both statistics (1) and (2) are given on Supplementary Table 3 with their associated p-values for the 2 sets of methods and the 4 sizes of dataset.

Table 3. Friedman test, correction by Iman and Davenport and Nemenyi test. CD is the critical distance and N the number of tasks for each size.

(a) Tree-based methods of Table 1.

Size	χ_F^2	χ_F^2 p-value	F_F	F_F p-value	CD	N
2500	73	9.6e-11	12	7.4e-16	4.6	13
10000	76	2.3e-11	15	6.1e-18	4.8	12
25000	30	2.5e-03	3.9	2.4e-04	6.3	7
100000	10	0.43	1.2	0.35	6.8	4

(b) Boosted-trees and linear methods of Supplementary Table 2.

Size	χ_F^2	χ_F^2 p-value	F_F	F_F p-value	CD	N
2500	41	5.1e-06	7.8	5.2e-08	3.3	13
10000	50	9.7e-08	12	1.7e-11	3.5	12
25000	23	5.6e-03	4.3	6.2e-04	4.5	7
100000	-19	1	-1.1	1	6	4

Figure 3. Mean ranks by method and by size of dataset. The critical difference is computed using the Nemenyi test (equation (3) and Supplementary Table 3). Methods within the critical difference range do not perform significantly differently from one another according to the Nemenyi test. Methods within the critical difference range of MIA are in red, others in black.

(a) Tree-based methods.

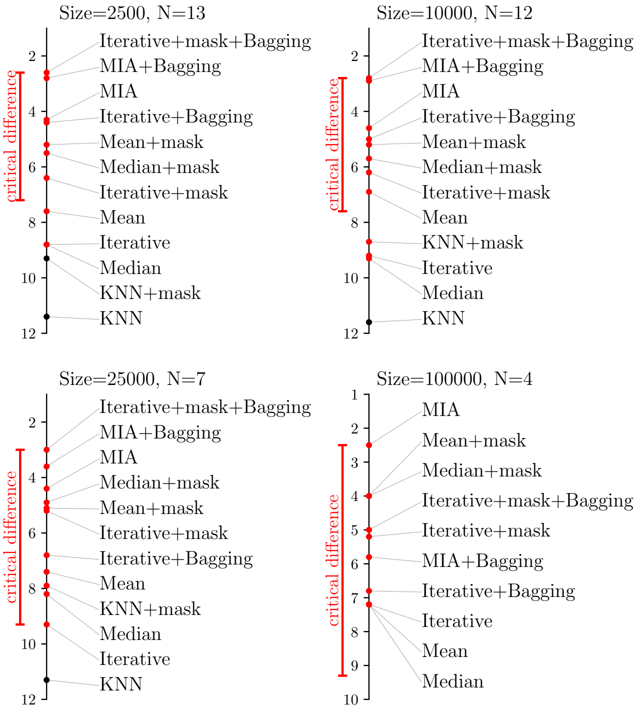

(b) Boosted trees+MIA vs linear methods.

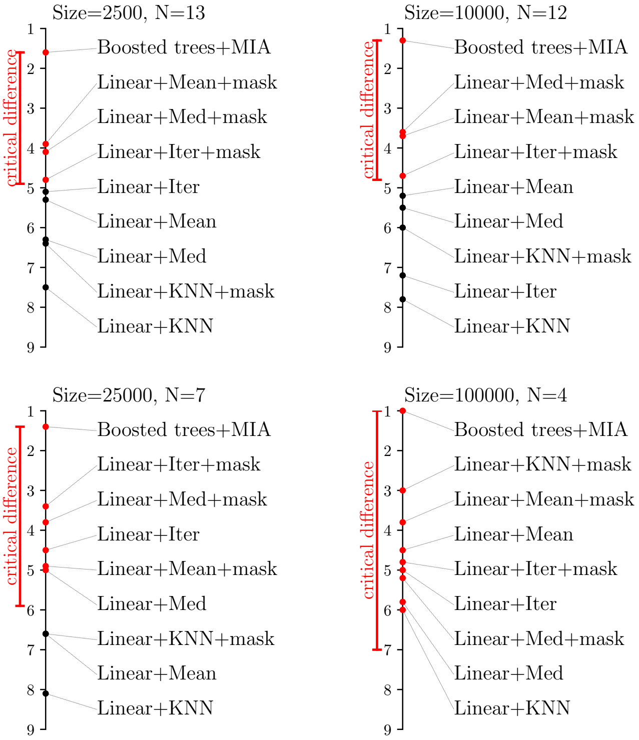

Nemenyi test. Once the Friedman test is rejected, the Nemenyi test can be applied. It provides a critical difference CD which is the minimal difference between the average ranks of two algorithms for them to be significantly different.

$$CD = q_\alpha \sqrt{\frac{k(k+1)}{6N}} \quad (3)$$

Values of q_α are given in Table 5 of Demšar (2006). Values of critical differences for the 2 sets of methods and the 4 sizes of dataset are given in Supplementary Table 3.

Wilcoxon signed-rank test. To compute the one-sided Wilcoxon signed-rank test, we used the `wilcoxon` function of the `scipy.stats` module between the 13 average scores of MIA against the ones of every other methods. Resulting p-values are given in Supplementary Table 4 for the 4 sizes of dataset.

Table 4. One-sided Wilcoxon signed-rank test. p-values of the one-sided right test on the difference of score between MIA and every other method for Table 4a, and between gradient-boosted trees and linear models for Table 4b. p-values below the 0.05 level are marked with *. p-values below the Bonferroni corrected level are marked with **. When the reversed test (*i.e.* one-sided left) is significant instead, p-values are marked with *(>) and **(>) following the same rule.

(a) MIA vs imputation. Bonferroni level: $0.05/19 = 2.6 \times 10^{-3}$. Rejecting the null hypothesis means MIA performed better than the compared method.

Size / Method	2500	10000	25000	100000
Mean	1.2e-03**	4.6e-02*	2.3e-02*	6.2e-02
Mean+mask	4.0e-02*	2.3e-01	1.5e-01	6.2e-02
Median	5.2e-03*	1.7e-03**	2.3e-02*	6.2e-02
Median+mask	4.0e-02*	2.1e-01	1.5e-01	1.2e-01
Iterative	5.2e-03*	3.2e-02*	3.9e-02*	6.2e-02
Iterative+mask	2.4e-02*	2.1e-01	4.7e-01	6.2e-02
KNN	1.2e-04**	2.4e-04**	3.1e-02*	
KNN+mask	1.2e-04**	7.3e-04**	3.1e-02*	
MI	8.1e-01	6.6e-01	3.4e-01	1.2e-01
MI+mask	9.9e-01*(>)	9.6e-01*(>)	9.2e-01	4.4e-01
MIA+bagging	9.7e-01*(>)	9.4e-01	7.7e-01	3.1e-01
Linear+Mean	6.1e-04**	4.9e-04**	7.8e-03*	6.2e-02
Linear+Mean+mask	8.5e-04**	7.3e-04**	1.6e-02*	6.2e-02
Linear+Med	6.1e-04**	4.9e-04**	7.8e-03*	6.2e-02
Linear+Med+mask	6.1e-04**	4.9e-04**	1.6e-02*	6.2e-02
Linear+Iter	3.1e-03*	1.2e-03**	1.6e-02*	6.2e-02
Linear+Iter+mask	2.3e-03**	1.2e-03**	1.6e-02*	6.2e-02
Linear+KNN	1.2e-04**	2.4e-04**	1.6e-02*	5.0e-01
Linear+KNN+mask	1.2e-04**	2.4e-04**	3.1e-02*	5.0e-01

(b) Gradient-boosted trees vs linear models. Bonferroni level: $0.05/8 = 6.25 \times 10^{-3}$. Rejecting the null hypothesis means gradient-boosted trees performed better than linear models for the given imputer.

Size / Imputer	2500	10000	25000	100000
Mean	1.2e-03**	4.9e-04**	1.6e-02*	6.2e-02
Mean+mask	1.7e-03**	4.9e-04**	1.6e-02*	6.2e-02
Median	6.1e-04**	7.3e-04**	1.6e-02*	6.2e-02
Median+mask	8.5e-04**	2.4e-04**	1.6e-02*	6.2e-02
Iterative	4.0e-03**	1.2e-03**	2.3e-02*	6.2e-02
Iterative+mask	2.3e-03**	1.2e-03**	1.6e-02*	6.2e-02
KNN	8.5e-04**	7.3e-04**	3.1e-02*	
KNN+mask	8.5e-04**	4.9e-04**	3.1e-02*	

Table 5. Scikit-learn's implementations of the methods.

In-article name	Scikit-learn's method
Boosted trees	`HistGradientBoostingRegressor`, `HistGradientBoostingClassifier`
Linear model	`Ridge`, `LogisticRegression`
Mean, Mean+mask	`SimpleImputer`
Median, Median+mask	`SimpleImputer`
Iterative, Iterative+mask	`IterativeImputer`
KNN, KNN+mask	`KNNImputer`
ANOVA selection	`f_regression`, `f_classif`
Permutation importance	`permutation_importance`
Bagging	`BaggingRegressor`, `BaggingClassifier`

Table 6. Correlation between features. Average number of ordinal and numerical features correlated to other ordinal or numerical features with an absolute correlation coefficient larger than thresholds $\{0.1, 0.2, 0.3\}$, averaged on all ordinal and numerical features of the task and expressed in percentage of the number of ordinal and numerical features in the task. For example in the task "death_screening", a numerical or ordinal feature has an absolute correlation value greater than 0.01 with 68% of the ordinal and numerical features of the task in average.

Database	Task	# features	Threshold 0.1	0.2	0.3
Traumabase	death_screening	92	68%	41%	22%
	hemo	12	50%	23%	12%
	hemo_screening	76	65%	36%	20%
	platelet_screening	90	67%	40%	22%
	septic_screening	76	68%	37%	18%
UKBB	breast_25	11	40%	20%	19%
	breast_screening	100	26%	12%	8%
	fluid_screening	100	21%	10%	6%
	parkinson_screening	100	28%	16%	11%
	skin_screening	100	24%	11%	8%
MIMIC	hemo_screening	100	22%	6%	3%
	septic_screening	100	21%	6%	2%
NHIS	income_screening	78	15%	6%	4%
Average		79	40%	20%	12%

Figure 4. Types of features. Number of categorical, ordinal and numerical features in each dataset, *before encoding*. Note that one non-encoded categorical feature can lead to several selected encoded features. Since we select 100 encoded features, some task have less than 100 non-encoded features. For tasks having several trials, five horizontal bars are plotted representing one trial each, as feature selection may select different features.

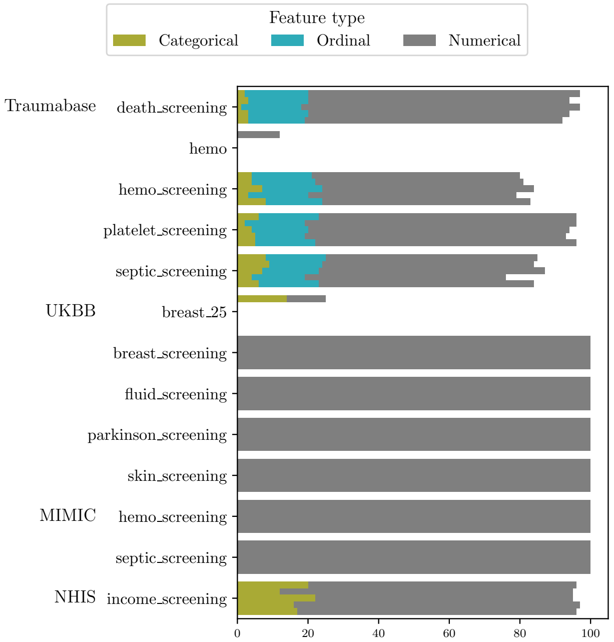

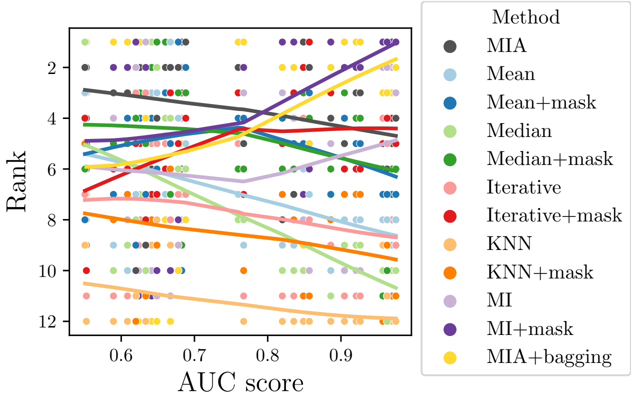

(a) Classification tasks

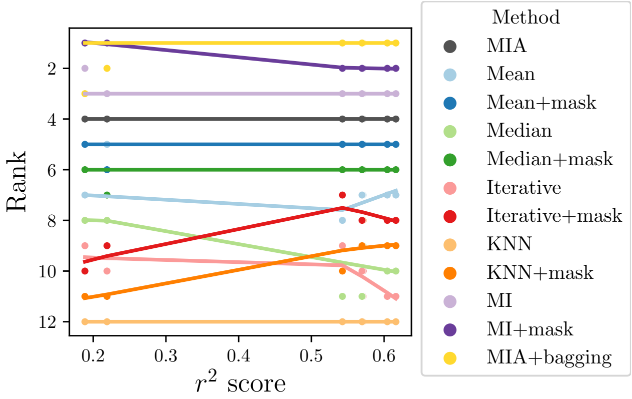

(b) Regression tasks

Figure 5. Effect of difficulty on the ranks of the methods. For each task and size, the average score obtained by the methods is taken as a proxy of its difficulty. Local regressions (LOWESS) are plotted for each method to better visualize trends.

Effect of tasks' difficulty on the performance of the methods. For classification tasks, Supplementary Figure 5a shows the relative performance of the methods as a function of the tasks' difficulty. Bagged methods Iterative+mask+Bagging and MIA+Bagging show a clear trend with lower (resp. higher) ranks for easier (resp. harder) methods. Also, MIA is the best performing one for harder tasks (for AUC < 0.8). Thus, the interest of MIA seems more pronounced for harder tasks. There is not enough regression tasks to observe exploitable trends on Supplementary Figure 5b.

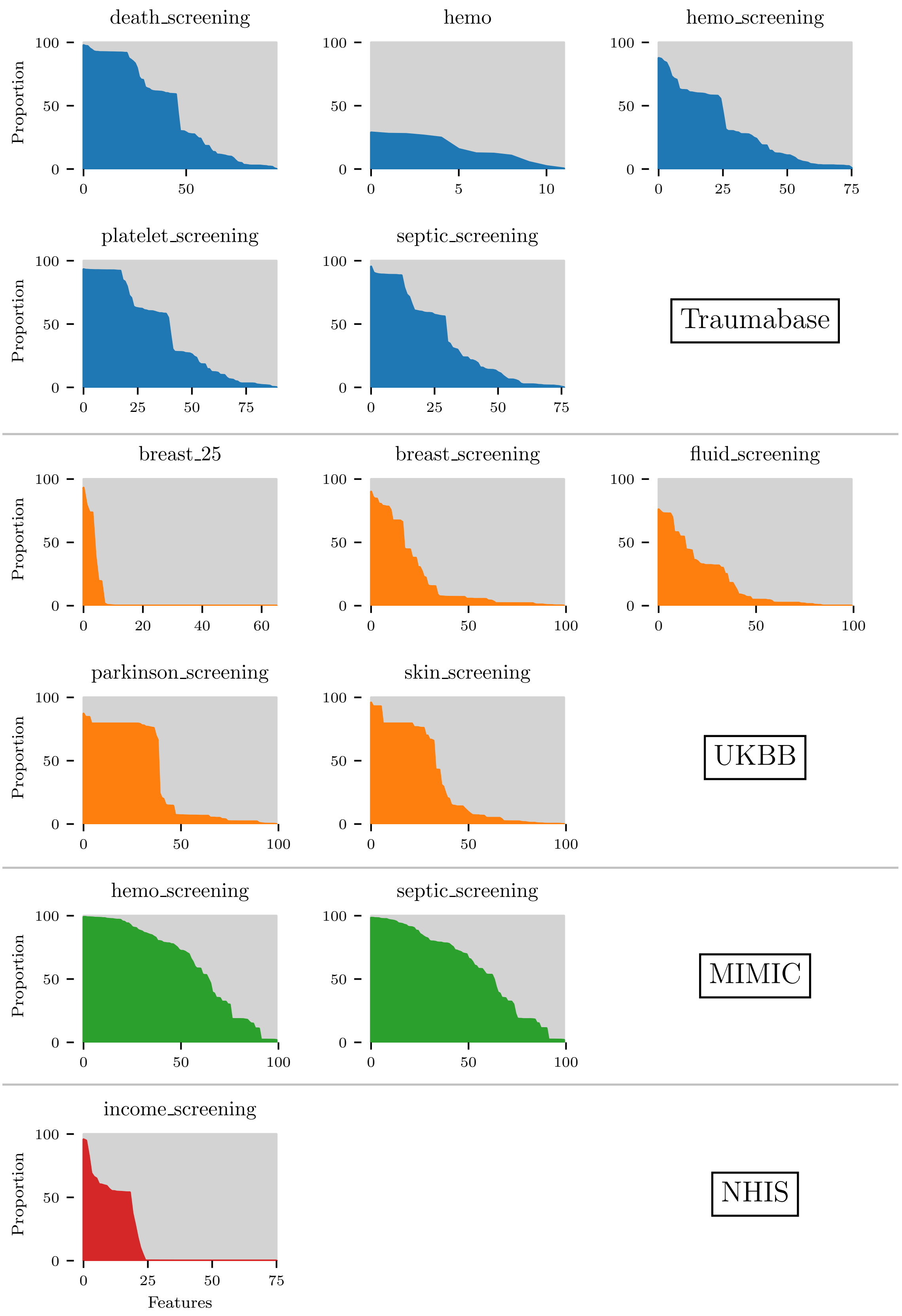

Figure 6. Missing values distribution. Proportion of missing values across selected encoded features for each task and for trial number 1, sorted in decreasing order. Other trials have similar proportions.

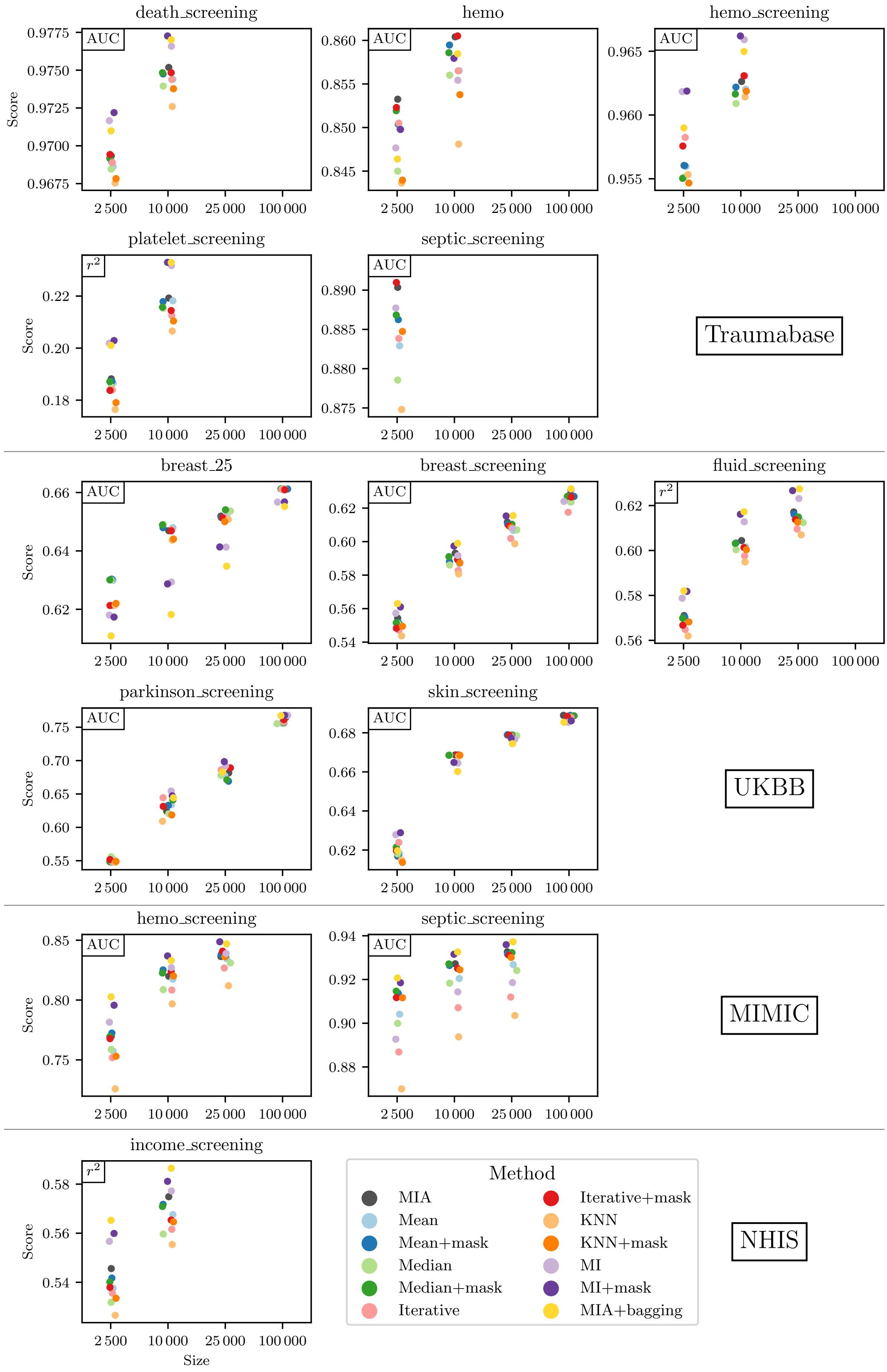

Figure 7. Scores of the tree-based methods as a function of the training size. Detailed scores of Figure 1 broken out by task.

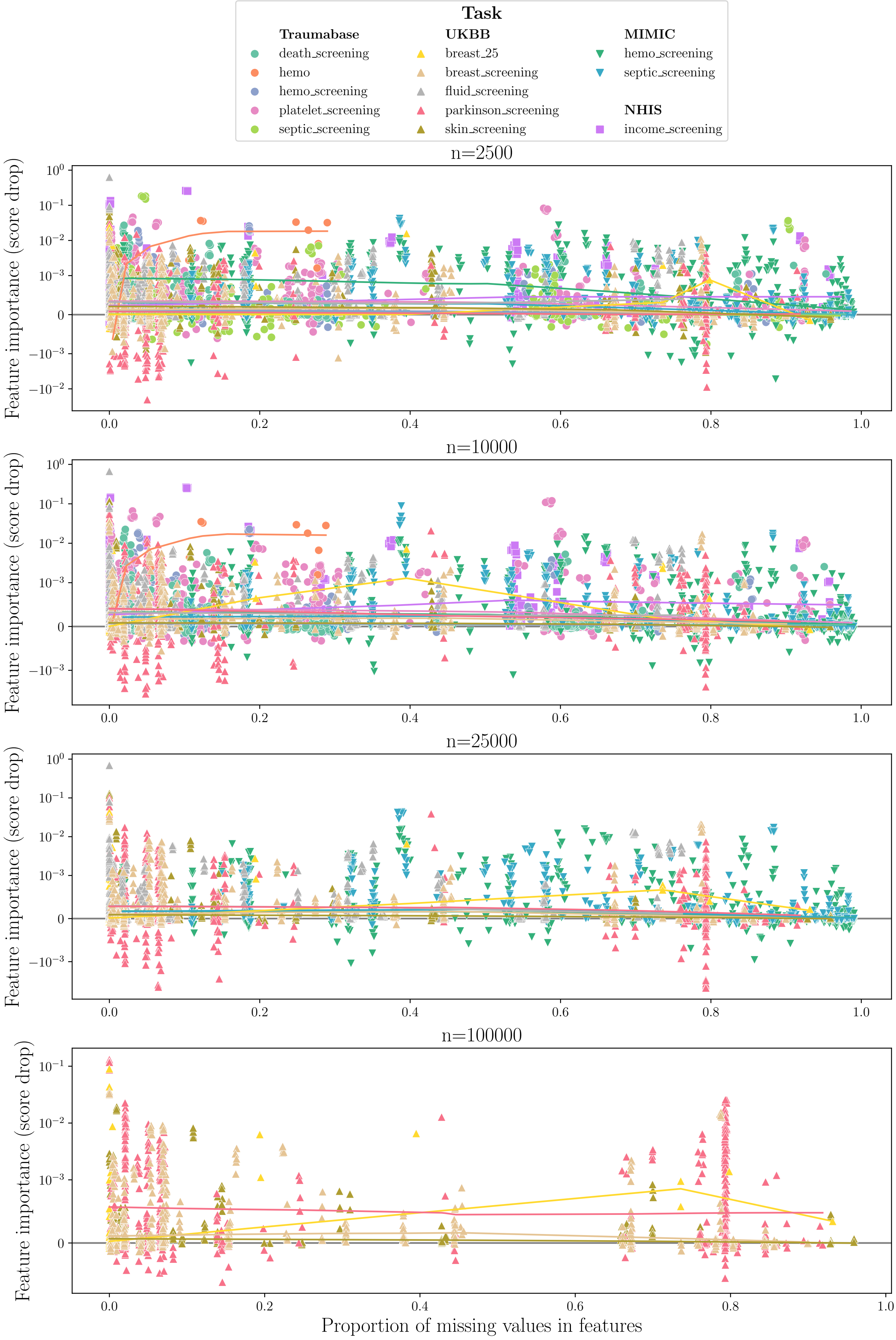

Figure 8. Feature importance versus proportion of missing values. Importance is measured as the drop in score when randomly permuting the considered feature. Each feature is permuted 10 times and its importance is taken as the average drop in score. Score drops are also averaged across folds. Local regressions (LOWESS) are plotted for each task to better visualize trends.

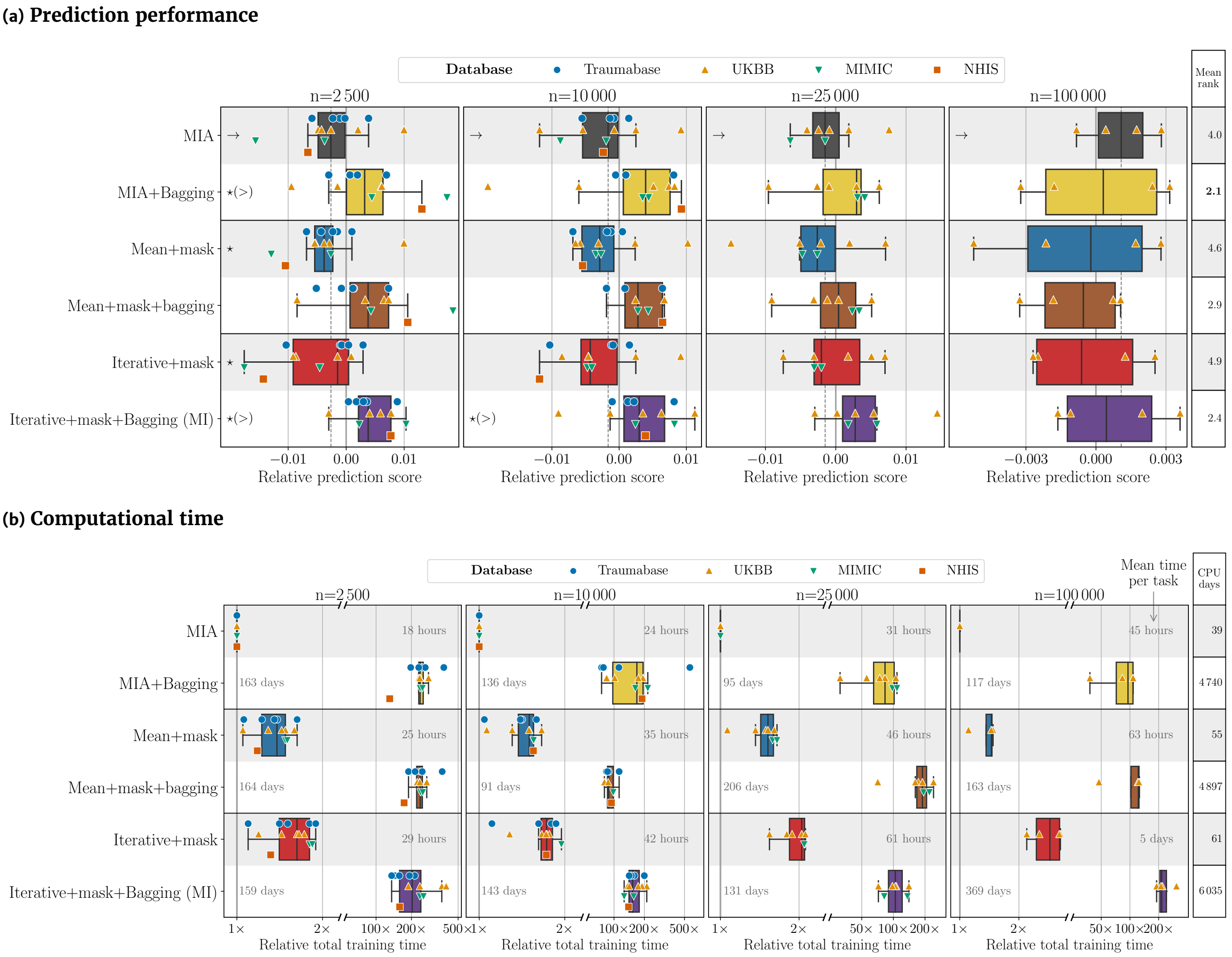

Figure 9. Effect of bagging. Comparison of prediction performance and training times between MIA, Mean+mask, Iterative+mask and their bagged version, for 13 prediction tasks spread over 4 databases, and for 4 sizes of dataset (2 500, 10 000, 25 000 and 100 000 samples). This figure is based on Figure 1, refer to caption of Figure 1 for more details.

Table 7. Overview of the prediction tasks used in this article. For selection, 'A' means ANOVA and 'M' means manual. Type 'C' is classification and type 'R' is regression. The number of features is given after encoding and selection. Since this number may vary between trials, we average it on the 5 trials for the ANOVA selection. Target is the name of the feature to predict in the original database or a formula to build a new feature to predict from the existing ones.

Database	Task	Score	Scorer	Selection	Number of samples	Number of features	Type	Target	Description
Traumabase	death_screening	0.96	AUC	A	12341	98	C	Décès == Oui	Predict the death of patients.
	hemo	0.85	AUC	M	19569	12	C	Choc hémorragique (? 4 CGR sur 6h) == Oui	Predict the hemorrhagic shock using features defined in Jiang et al. (2020).
	hemo_screening	0.95	AUC	A	13047	89	C	Choc hémorragique (? 4 CGR sur 6h) == Oui	Predict the hemorrhagic shock using ANOVA selection.
	platelet_screening	0.17	R2	A	12696	96	R	Plaquettes	Predict the level of platelet on arrival at the hospital using ANOVA selection.
	septic_screening	0.86	AUC	A	6046	90	C	Choc septique == Oui	Predict septic shock.
UKBB	breast_25	0.60	AUC	M	273384	66	C	One of: C500, C501, C502, C503, C504, C505, C506, C508, C509; found in one of: 41270-0.0, 41202-0.0, 41204-0.0, 40006-0.0; or one of: 1740, 1743, 1744, 1745, 1748, 1749; found in one of: 41271-0.0, 41203-0.0, 41205-0.0, 40013-0.0	Predict malignant neoplasm of breast on female patients only using features defined in Läll et al. (2019).
	breast_screening	0.59	AUC	A	182257	100	C	Same as breast_25	Predict malignant neoplasm of breast on female patients only using ANOVA selection.
	fluid_screening	0.56	R2	A	110308	100	R	20016-0.0	Predict the fluid intelligence score.
	parkinson_screening	0.65	AUC	A	335005	100	C	One of: G20, G210, G211, G212, G213, G214, G218, G219, G22, F023; found in one of: 41270-0.0, 41202-0.0, 41204-0.0, 40006-0.0; or one of: 3320, 3321; found in one of: 41271-0.0, 41203-0.0, 41205-0.0, 40013-0.0	Predict Parkinson's disease.
	skin_screening	0.64	AUC	A	335005	100	C	One of: C430, C431, C432, C433, C434, C435, C436, C437, C438, C439, C440, C441, C442, C443, C444, C445, C446, C447, C448, C449; found in one of: 41270-0.0, 41202-0.0, 41204-0.0, 40006-0.0; or one of: 1720, 1723, 1725, 1726, 1727, 1729, 1730, 1731, 1732, 1733, 1734, 1735, 1736, 1737, 1739; found in one of: 41271-0.0, 41203-0.0, 41205-0.0, 40013-0.0	Predict melanoma and other malignant neoplasms of skin.
MIMIC	hemo_screening	0.74	AUC	A	30836	100	C	One of: 78559, 99809, 9584; found in ICD9_CODE	Predict the hemorrhagic shock from the LABEVENTS table only.
	septic_screening	0.87	AUC	A	30836	100	C	ICD9_CODE == 78552	Predict the septic shock from the LABEVENTS table only.
NHIS	income_screening	0.52	R2	A	20987	96	R	ERNYR-P	Predict the income earned on the previous year with information from tables: household, family, person and adult.

Table 8. Scores and ranks of the tree based methods described in Table 1.

(a) Scores relative to the absolute reference score plotted in Figure 1a. Values in bold are the reference scores and are absolute. Other scores are given relative to the reference score of their task and size.

	Database	Traumabase					UKBB					MIMIC		NHIS
Size	Task<br>Method	death_screening	hemo	hemo_screening	platelet_screening	septic_screening	breast_25	breast_screening	fluid_screening	parkinson_screening	skin_screening	hemo_screening	septic_screening	income_screening
2500	MIA	-8e-5	+5e-3	-1e-3	-4e-4	+4e-3	+7e-3	+2e-3	+5e-5	+2e-3	-3e-3	+3e-3	+8e-3	+3e-3
	Mean	-8e-4	+1e-3	-1e-3	-2e-3	-3e-3	+6e-3	-1e-3	-2e-3	+2e-3	-2e-3	-1e-2	-6e-4	-5e-3
	Mean+mask	-5e-4	+2e-3	-1e-3	-1e-3	+2e-4	+7e-3	-8e-4	-5e-4	-2e-3	-3e-3	+5e-3	+9e-3	-1e-3
	Median	-1e-3	-4e-3	-2e-3	-3e-3	-8e-3	+6e-3	-1e-3	-4e-3	+6e-3	-2e-3	-8e-3	-5e-3	-1e-2
	Median+mask	-2e-4	+3e-3	-2e-3	-1e-3	+7e-4	+7e-3	-9e-4	-1e-3	-2e-3	+1e-3	+2e-3	+1e-2	-3e-3
	Iterative	-5e-4	+2e-3	+1e-3	-5e-3	-2e-3	-2e-3	-5e-3	-6e-3	-2e-3	+4e-3	-2e-2	-2e-2	-7e-3
	Iterative+mask	+4e-6	+4e-3	+3e-4	-5e-3	+5e-3	-2e-3	-4e-3	-4e-3	+1e-3	-4e-4	+6e-4	+7e-3	-5e-3
	KNN	-2e-3	-5e-3	-2e-3	-1e-2	-1e-2	-2e-3	-9e-3	-9e-3	-3e-3	-5e-3	-4e-2	-3e-2	-2e-2
	KNN+mask	-2e-3	-5e-3	-3e-3	-1e-2	-1e-3	-2e-3	-3e-3	-3e-3	-1e-3	-6e-3	-1e-2	+7e-3	-9e-3
	Iterative+Bagging	+2e-3	-1e-3	+5e-3	+1e-2	+2e-3	-6e-3	+5e-3	+8e-3		+8e-3	+1e-2	-1e-2	+1e-2
	Iterative+mask+Bagging	+3e-3	+1e-3	+5e-3	+1e-2	+7e-3	-6e-3	+9e-3	+1e-2		+9e-3	+3e-2	+1e-2	+2e-2
	MIA+Bagging	+2e-3	-2e-3	+2e-3	+1e-2	+6e-3	-1e-2	+1e-2	+1e-2		-4e-4	+4e-2	+2e-2	+2e-2
	Reference score	**0.97**	**0.85**	**0.96**	**0.19**	**0.89**	**0.62**	**0.55**	**0.57**	**0.55**	**0.62**	**0.77**	**0.90**	**0.54**
10000	MIA	+2e-4	+4e-3	-4e-4	+3e-4		+5e-3	+4e-3	-1e-5	-9e-3	+1e-3	-1e-4	+6e-3	+5e-3
	Mean	-6e-4	-3e-4	-1e-3	-8e-4		+6e-3	-3e-3	-3e-3	+3e-4	+2e-3	-3e-3	-2e-4	-2e-3
	Mean+mask	-2e-4	+3e-3	-8e-4	-1e-3		+6e-3	-1e-3	-1e-3	-6e-4	+1e-3	+5e-3	+6e-3	+2e-3
	Median	-1e-3	-8e-4	-2e-3	-4e-3		+8e-3	-3e-3	-4e-3	-1e-2	+1e-3	-1e-2	-2e-3	-1e-2
	Median+mask	-1e-4	+2e-3	-1e-3	-3e-3		+7e-3	+2e-3	-1e-3	+8e-3	+1e-3	+3e-3	+6e-3	+1e-3
	Iterative	-6e-4	-3e-4	+8e-5	-6e-3		+6e-3	-7e-3	-7e-3	+1e-2	+1e-3	-1e-2	-1e-2	-8e-3
	Iterative+mask	-1e-4	+4e-3	+9e-5	-5e-3		+5e-3	-1e-4	-3e-3	-2e-3	+1e-3	+4e-3	+4e-3	-4e-3
	KNN	-2e-3	-9e-3	-2e-3	-1e-2		+2e-3	-9e-3	-9e-3	-2e-2	+1e-3	-2e-2	-3e-2	-1e-2
	KNN+mask	-1e-3	-3e-3	-1e-3	-9e-3		+2e-3	-2e-3	-4e-3	-1e-2	+1e-3	+1e-4	+4e-3	-5e-3
	Iterative+Bagging	+2e-3	-1e-3	+3e-3	+1e-2		-1e-2	+2e-3	+8e-3	+2e-2	-3e-3	+7e-3	-6e-3	+7e-3
	Iterative+mask+Bagging	+2e-3	+1e-3	+3e-3	+1e-2		-1e-2	+8e-3	+1e-2	+1e-2	-2e-3	+2e-2	+1e-2	+1e-2
	MIA+Bagging	+2e-3	+2e-3	+2e-3	+1e-2		-2e-2	+1e-2	+1e-2	+1e-2	-7e-3	+1e-2	+1e-2	+2e-2
	Reference score	**0.97**	**0.86**	**0.96**	**0.22**		**0.64**	**0.59**	**0.60**	**0.63**	**0.67**	**0.82**	**0.92**	**0.57**
25000	MIA						+3e-3	+3e-3	+9e-4	-1e-3	+9e-4	+8e-4	+6e-3	
	Mean						+4e-3	-2e-3	-2e-3	-5e-3	+9e-4	-1e-3	+5e-4	
	Mean+mask						+3e-3	+2e-3	-1e-4	-1e-2	+1e-3	+2e-3	+5e-3	
	Median						+5e-3	-2e-3	-4e-3	-5e-3	+5e-4	-5e-3	-2e-3	
	Median+mask						+5e-3	+2e-3	-1e-3	-1e-2	+9e-4	+1e-3	+6e-3	
	Iterative						+3e-3	-7e-3	-7e-3	+4e-3	+5e-4	-9e-3	-1e-2	
	Iterative+mask						+3e-3	+8e-4	-2e-3	+6e-3	+8e-4	+5e-3	+5e-3	
	KNN						+2e-3	-1e-2	-9e-3			-2e-2	-2e-2	
	KNN+mask						+1e-3	-1e-4	-4e-3			+6e-4	+4e-3	
	Iterative+Bagging						-7e-3	-8e-4	+7e-3	+1e-2	-1e-3	+3e-3	-8e-3	
	Iterative+mask+Bagging						-7e-3	+7e-3	+1e-2	+2e-2	-7e-4	+1e-2	+1e-2	
	MIA+Bagging						-1e-2	+7e-3	+1e-2	+4e-4	-4e-3	+1e-2	+1e-2	
	Reference score						**0.65**	**0.61**	**0.62**	**0.68**	**0.68**	**0.84**	**0.93**	
100000	MIA						+2e-3	+2e-3		+3e-3	+1e-3			
	Mean						+1e-3	-2e-3		-5e-3	+7e-4			
	Mean+mask						+2e-3	+9e-4		-2e-3	+1e-3			
	Median						+2e-3	-2e-3		-6e-3	+5e-4			
	Median+mask						+2e-3	+9e-4		-5e-3	+1e-3			
	Iterative						+1e-3	-9e-3		-4e-3	+2e-4			
	Iterative+mask						+1e-3	+6e-4		+1e-4	+8e-4			
	KNN													
	KNN+mask													
	Iterative+Bagging						-3e-3	-2e-3		+6e-3	-2e-3			
	Iterative+mask+Bagging						-3e-3	+5e-3		+6e-3	-2e-3			
	MIA+Bagging						-4e-3	+5e-3		+6e-3	-2e-3			
	Reference score						**0.66**	**0.63**		**0.76**	**0.69**			
Average	Reference score	**0.97**	**0.85**	**0.96**	**0.20**	**0.89**	**0.64**	**0.59**	**0.60**	**0.66**	**0.66**	**0.81**	**0.92**	**0.56**

(b) Ranks computed from the relative scores in Supplementary Table 8a. Best average ranks are in bold.

	Database	Traumabase					UKBB					MIMIC		NHIS	Average				
Size	Task<br>Method	death_screening	hemo	hemo_screening	platelet_screening	septic_screening	breast_25	breast_screening	fluid_screening	parkinson_screening	skin_screening	hemo_screening	septic_screening	income_screening	Traumabase	UKBB	MIMIC	NHIS	All
2500	MIA	5	1	6	4	4	1	4	4	2	10	5	5	4	4.0	4.2	5.0	4.0	4.3
	Mean	9	6	8	7	10	5	8	7	3	7	9	8	8	8.0	6.0	8.5	8.0	7.6
	Mean+mask	7	5	7	5	7	2	5	5	8	9	4	4	5	6.2	5.8	4.0	5.0	5.2
	Median	10	10	10	8	11	4	7	9	1	8	8	9	11	9.8	5.8	8.5	11.0	8.8
	Median+mask	6	3	11	6	6	3	6	6	6	4	6	3	6	6.4	5.0	4.5	6.0	5.5
	Iterative	8	4	4	9	9	9	11	11	7	3	11	11	9	6.8	8.2	11.0	9.0	8.8
	Iterative+mask	4	2	5	10	3	8	10	10	4	5	7	6	7	4.8	7.4	6.5	7.0	6.4
	KNN	12	12	9	12	12	7	12	12	9	11	12	12	12	11.4	10.2	12.0	12.0	11.4
	KNN+mask	11	11	12	11	8	6	9	8	5	12	10	7	10	10.6	8.0	8.5	10.0	9.3
	Iterative+Bagging	2	8	2	2	5	10	3	3		2	3	10	3	3.8	4.5	6.5	3.0	4.4
	Iterative+mask+Bagging	1	7	1	1	1	11	2	2		1	2	2	2	**2.2**	**4.0**	2.0	2.0	**2.6**
	MIA+Bagging	3	9	3	3	2	12	1	1		6	1	1	1	4.0	5.0	**1.0**	**1.0**	2.8
10000	MIA	4	2	6	4		6	3	4	9	2	8	3	4	4.0	**4.8**	5.5	4.0	4.6
	Mean	8	7	8	5		3	9	7	6	1	9	8	7	7.0	5.2	8.5	7.0	6.9
	Mean+mask	7	3	7	6		4	7	5	7	5	4	5	5	5.8	5.6	4.5	5.0	5.2
	Median	10	9	12	8		1	10	10	10	4	10	9	11	9.8	7.0	9.5	11.0	9.3
	Median+mask	6	4	10	7		2	5	6	5	7	6	4	6	6.8	5.0	5.0	6.0	5.7
	Iterative	9	8	5	10		5	11	11	3	8	11	11	10	8.0	7.6	11.0	10.0	9.2
	Iterative+mask	5	1	4	9		7	6	8	8	3	5	6	8	4.8	6.4	5.5	8.0	6.2
	KNN	12	12	11	12		9	12	12	12	9	12	12	12	11.8	10.8	12.0	12.0	11.6
	KNN+mask	11	11	9	11		8	8	9	11	6	7	7	9	10.5	8.4	7.0	9.0	8.7
	Iterative+Bagging	3	10	2	3		10	4	3	1	11	3	10	3	4.5	5.8	6.5	3.0	5.0
	Iterative+mask+Bagging	1	6	1	1		11	2	2	2	10	1	2	2	**2.2**	5.4	**1.5**	2.0	**2.8**
	MIA+Bagging	2	5	3	2		12	1	1	4	12	2	1	1	3.0	6.0	1.5	**1.0**	2.9
25000	MIA						4	3	4	6	2	7	3			**3.8**	5.0		4.4
	Mean						3	10	7	8	4	9	8			6.4	8.5		7.4
	Mean+mask						6	4	5	10	1	5	5			5.2	5.0		5.1
	Median						2	9	10	7	6	10	9			6.8	9.5		8.2
	Median+mask						1	5	6	9	3	6	4			4.8	5.0		4.9
	Iterative						5	11	11	4	7	11	11			7.6	11.0		9.3
	Iterative+mask						7	6	8	3	5	3	6			5.8	4.5		5.2
	KNN						8	12	12			12	12			10.7	12.0		11.3
	KNN+mask						9	7	9			8	7			8.3	7.5		7.9
	Iterative+Bagging						11	8	3	2	9	4	10			6.6	7.0		6.8
	Iterative+mask+Bagging						10	2	2	1	8	1	2			4.6	**1.5**		**3.0**
	MIA+Bagging						12	1	1	5	10	2	1			5.8	1.5		3.6
100000	MIA						2	3		4	1					**2.5**			**2.5**
	Mean						7	8		9	5					7.2			7.2
	Mean+mask						3	5		6	2					4.0			4.0
	Median						4	9		10	6					7.2			7.2
	Median+mask						1	4		8	3					4.0			4.0
	Iterative						5	10		7	7					7.2			7.2
	Iterative+mask						6	6		5	4					5.2			5.2
	KNN																		
	KNN+mask																		
	Iterative+Bagging						9	7		1	10					6.8			6.8
	Iterative+mask+Bagging						8	2		2	8					5.0			5.0
	MIA+Bagging						10	1		3	9					5.8			5.8
Average	MIA	4.5	**1.5**	6.0	4.0	4.0	3.2	3.2	4.0	5.2	**3.8**	6.7	3.7	4.0	4.0	**3.9**	5.2	4.0	4.3
	Mean	8.5	6.5	8.0	6.0	10.0	4.5	8.8	7.0	6.5	4.2	9.0	8.0	7.5	7.8	6.2	8.5	7.5	7.5
	Mean+mask	7.0	4.0	7.0	5.5	7.0	3.8	5.2	5.0	7.8	4.2	4.3	4.7	5.0	6.1	5.2	4.5	5.0	5.2
	Median	10.0	9.5	11.0	8.0	11.0	2.8	8.8	9.7	7.0	6.0	9.3	9.0	11.0	9.9	6.8	9.2	11.0	9.2
	Median+mask	6.0	3.5	10.5	6.5	6.0	**1.8**	5.0	6.0	7.0	4.2	6.0	3.7	6.0	6.5	4.8	4.8	6.0	5.5
	Iterative	8.5	6.0	4.5	9.5	9.0	6.0	10.8	11.0	5.2	6.2	11.0	11.0	9.5	7.5	7.8	11.0	9.5	9.0
	Iterative+mask	4.5	1.5	4.5	9.5	3.0	7.0	7.0	8.7	5.0	4.2	5.0	6.0	7.5	4.6	6.4	5.5	7.5	6.0
	KNN	12.0	12.0	10.0	12.0	12.0	8.0	12.0	12.0	10.5	10.0	12.0	12.0	12.0	11.6	10.5	12.0	12.0	11.5
	KNN+mask	11.0	11.0	10.5	11.0	8.0	7.7	8.0	8.7	8.0	9.0	8.3	7.0	9.5	10.3	8.3	7.7	9.5	8.9
	Iterative+Bagging	2.5	9.0	2.0	2.5	5.0	10.0	5.5	3.0	**1.3**	8.0	3.3	10.0	3.0	4.2	5.6	6.7	3.0	4.9
	Iterative+mask+Bagging	**1.0**	6.5	**1.0**	**1.0**	**1.0**	10.0	2.0	2.0	1.7	6.8	**1.3**	2.0	2.0	**2.1**	4.5	1.7	2.0	**2.6**
	MIA+Bagging	2.5	7.0	3.0	2.5	2.0	11.5	**1.0**	**1.0**	4.0	9.2	1.7	**1.0**	**1.0**	3.4	5.4	**1.3**	**1.0**	2.8

Table 9. Scores and ranks of gradient-boosted trees+MIA compared to linear methods described in Supplementary Table 2.
We removed one outlier fold from one trial for the methods Linear+Iter and Linear+Iter+mask for the "task platelet_screening" at size n=2 500. Others are unchanged.

(a) Scores relative to the absolute reference score and plotted in Supplementary Figure 2a. Values in bold are the reference scores and are absolute. Other scores are given relative to the reference score of their task and size.

Size	Database / Task / Method	Traumabase: death_screening	Traumabase: hemo	Traumabase: hemo_screening	Traumabase: platelet_screening	Traumabase: septic_screening	UKBB: breast_25	UKBB: breast_screening	UKBB: fluid_screening	UKBB: parkinson_screening	UKBB: skin_screening	MIMIC: hemo_screening	MIMIC: septic_screening	NHIS: income_screening
2500	Boosted trees+MIA	+1e-2	+9e-3	+2e-2	+1e-1	+5e-2	+8e-2	-2e-3	+6e-2	-8e-3	+2e-2	+1e-1	+1e-1	+5e-2
	Linear+Mean	+8e-4	-3e-3	-3e-3	+8e-3	-4e-3	-6e-3	+2e-4	-1e-2	+2e-3	-6e-3	-1e-2	-2e-2	-2e-2
	Linear+Mean+mask	+6e-4	+2e-4	-2e-3	-2e-3	-4e-3	-5e-4	+1e-3	+8e-4	+2e-3	-4e-3	-2e-2	-1e-2	+7e-3
	Linear+Med	-7e-4	-8e-3	-3e-3	+6e-3	-4e-3	+2e-3	+3e-4	-1e-2	-5e-4	-5e-3	-2e-2	+1e-2	-2e-2
	Linear+Med+mask	-3e-4	-7e-3	-3e-3	-2e-3	-4e-3	+3e-3	+8e-4	+8e-4	-3e-4	-3e-3	-1e-2	+1e-2	+7e-3
	Linear+Iter	+2e-3	+4e-3	+8e-5	-5e-2	-6e-6	-1e-2	+3e-3	-2e-2	+2e-2	+9e-3	-1e-2	-3e-2	-2e-2
	Linear+Iter+mask	-2e-2	+7e-3	-5e-4	-8e-2	+1e-3	-1e-2	+2e-3	-7e-3	+2e-2	+1e-2	-2e-2	-3e-2	+3e-3
	Linear+KNN	+1e-3	-3e-3	-5e-3	+9e-3	-2e-2	-3e-2	-2e-3	-8e-3	-1e-2	-1e-2	-2e-2	-2e-2	-2e-2
	Linear+KNN+mask	+7e-4	+8e-4	-4e-3	+5e-3	-2e-2	-3e-2	-3e-3	+2e-3	-1e-2	-1e-2	-2e-2	-2e-2	+3e-3
	Reference score	**0.96**	**0.84**	**0.94**	**0.09**	**0.84**	**0.55**	**0.56**	**0.51**	**0.56**	**0.59**	**0.64**	**0.81**	**0.49**
10000	Boosted trees+MIA	+2e-2	+1e-2	+3e-2	+4e-2		+9e-2	+1e-2	+7e-2	-1e-2	+6e-2	+1e-1	+8e-2	+7e-2
	Linear+Mean	+1e-2	-9e-4	+8e-4	-4e-3		-1e-2	+8e-4	-2e-2	+2e-4	-1e-2	-1e-2	-2e-2	-2e-2
	Linear+Mean+mask	+1e-2	+4e-3	+9e-4	+1e-3		-1e-2	-2e-4	+1e-3	+7e-4	-7e-3	-2e-2	-1e-2	+1e-2
	Linear+Med	+9e-3	-6e-3	+5e-4	-6e-3		-1e-2	-7e-4	-2e-2	-5e-3	-9e-3	-8e-3	+8e-3	-2e-2
	Linear+Med+mask	+9e-3	+2e-3	+5e-4	+1e-3		-1e-2	+9e-4	+1e-3	-5e-3	-1e-2	-9e-3	+1e-2	+1e-2
	Linear+Iter	-3e-2	-1e-3	-2e-2	-1e-2		-2e-3	-3e-3	-3e-2	+3e-2	+1e-2	-2e-2	-2e-2	-4e-2
	Linear+Iter+mask	-5e-2	+5e-3	-1e-2	-6e-3		-1e-3	-3e-3	-7e-3	+3e-2	+9e-3	-7e-3	-2e-2	-6e-4
	Linear+KNN	+1e-2	-9e-3	-1e-3	-8e-3		-2e-2	-3e-3	-9e-3	-2e-2	-2e-2	-2e-2	-2e-2	-2e-2
	Linear+KNN+mask	+1e-2	-5e-3	-9e-4	-4e-3		-2e-2	-3e-3	+4e-3	-1e-2	-2e-2	-2e-2	-2e-2	+5e-3
	Reference score	**0.95**	**0.85**	**0.94**	**0.18**		**0.56**	**0.58**	**0.53**	**0.63**	**0.61**	**0.70**	**0.84**	**0.51**
25000	Boosted trees+MIA						+8e-2	+2e-2	+8e-2	-7e-3	+6e-2	+1e-1	+8e-2	
	Linear+Mean						-1e-2	+9e-4	-2e-2	-7e-3	-1e-2	-2e-2	-2e-2	
	Linear+Mean+mask						-1e-2	+1e-3	+1e-3	-7e-3	-7e-3	-1e-2	-1e-2	
	Linear+Med						-1e-2	-9e-4	-2e-2	-9e-3	-2e-2	-4e-3	+6e-3	
	Linear+Med+mask						-7e-3	-1e-3	+1e-3	-6e-3	-1e-2	-1e-2	+9e-3	
	Linear+Iter						-5e-3	-8e-3	-3e-2	+2e-2	+1e-2	-1e-4	-1e-2	
	Linear+Iter+mask						-9e-4	-8e-3	-8e-3	+2e-2	+1e-2	+1e-3	-1e-2	
	Linear+KNN						-2e-2	-2e-3	-1e-2		-4e-2	-3e-2	-2e-2	
	Linear+KNN+mask						-2e-2	-2e-3	+3e-3			-3e-2	-2e-2	
	Reference score						**0.57**	**0.59**	**0.54**	**0.69**	**0.62**	**0.73**	**0.85**	
100000	Boosted trees+MIA						+6e-2	+3e-2		+2e-2	+5e-2			
	Linear+Mean						-1e-2	-8e-4		-5e-3	-2e-2			
	Linear+Mean+mask						-8e-3	-1e-3		-3e-3	-2e-2			
	Linear+Med						-9e-3	-2e-3		-7e-3	-2e-2			
	Linear+Med+mask						-6e-3	-4e-3		-6e-3	-2e-2			
	Linear+Iter						-1e-2	-8e-3		-4e-4	+9e-3			
	Linear+Iter+mask						-1e-2	-7e-3		+4e-4	+9e-3			
	Linear+KNN							-3e-3						
	Linear+KNN+mask							-1e-3						
	Reference score						**0.60**	**0.60**		**0.74**	**0.64**			
Average	Reference score	**0.96**	**0.85**	**0.94**	**0.14**	**0.84**	**0.57**	**0.58**	**0.53**	**0.66**	**0.62**	**0.69**	**0.83**	**0.50**

(b) Ranks computed from relative scores in Supplementary Table 9a. Best average ranks are in bold.

Size	Database / Task / Method	Traumabase: death_screening	Traumabase: hemo	Traumabase: hemo_screening	Traumabase: platelet_screening	Traumabase: septic_screening	UKBB: breast_25	UKBB: breast_screening	UKBB: fluid_screening	UKBB: parkinson_screening	UKBB: skin_screening	MIMIC: hemo_screening	MIMIC: septic_screening	NHIS: income_screening	Average: Traumabase	Average: UKBB	Average: MIMIC	Average: NHIS	Average: All
2500	Boosted trees+MIA	1	1	1	1	1	1	7	1	7	1	1	1	1	**1.0**	**3.4**	**1.0**	**1.0**	**1.6**
	Linear+Mean	4	7	6	3	6	5	6	7	3	7	3	6	6	5.2	5.6	4.5	6.0	5.3
	Linear+Mean+mask	6	5	4	7	4	4	3	3	4	5	5	4	2	5.2	3.8	4.5	2.0	3.9
	Linear+Med	8	9	7	4	5	3	5	8	6	6	7	3	8	6.6	5.6	5.0	8.0	6.3
	Linear+Med+mask	7	8	5	6	7	2	4	4	5	4	4	2	3	6.6	3.8	3.0	3.0	4.1
	Linear+Iter	2	3	2	8	3	7	1	9	1	3	2	9	7	3.6	4.2	5.5	7.0	5.1
	Linear+Iter+mask	9	2	3	9	2	6	2	5	2	2	6	8	4	5.0	3.4	7.0	4.0	4.8
	Linear+KNN	3	6	9	2	9	9	8	6	9	9	9	5	9	5.8	8.2	7.0	9.0	7.5
	Linear+KNN+mask	5	4	8	5	8	8	9	2	8	8	8	7	5	6.0	7.0	7.5	5.0	6.4
10000	Boosted trees+MIA	1	1	1	1		1	1	1	7	1	1	1	1	**1.0**	**2.2**	**1.0**	**1.0**	**1.3**
	Linear+Mean	4	5	3	4		7	3	7	4	6	5	6	6	4.0	5.4	5.5	6.0	5.2
	Linear+Mean+mask	5	3	2	3		6	4	4	3	4	7	4	2	3.2	4.2	5.5	2.0	3.7
	Linear+Med	7	8	4	6		5	5	8	6	5	3	3	7	6.2	5.8	3.0	7.0	5.5
	Linear+Med+mask	6	4	5	2		4	2	3	5	7	4	2	3	4.2	4.2	3.0	3.0	3.6
	Linear+Iter	8	6	9	9		3	7	9	2	2	6	8	9	8.0	4.6	7.0	9.0	7.2
	Linear+Iter+mask	9	2	8	7		2	8	5	1	3	2	5	5	6.5	3.8	3.5	5.0	4.7
	Linear+KNN	2	9	7	8		9	6	6	9	8	9	9	8	6.5	7.6	9.0	8.0	7.8
	Linear+KNN+mask	3	7	6	5		8	9	2	8	9	8	7	4	5.2	7.2	7.5	4.0	6.0
25000	Boosted trees+MIA						1	1	1	5	1	1	1			**1.8**	**1.0**		**1.4**
	Linear+Mean						7	3	7	6	5	7	8			5.6	7.5		6.6
	Linear+Mean+mask						5	2	4	4	4	6	6			3.8	6.0		4.9
	Linear+Med						6	4	8	7	7	4	3			6.4	3.5		5.0
	Linear+Med+mask						4	5	3	3	6	5	2			4.2	3.5		3.8
	Linear+Iter						3	9	9	1	3	3	5			5.0	4.0		4.5
	Linear+Iter+mask						2	8	5	2	2	2	4			3.8	3.0		3.4
	Linear+KNN						9	6	6		8	9	9			7.2	9.0		8.1
	Linear+KNN+mask						8	7	2			8	7			5.7	7.5		6.6
100000	Boosted trees+MIA						1	1		1	1					**1.0**			**1.0**
	Linear+Mean						6	2		5	5					4.5			4.5
	Linear+Mean+mask						3	4		4	4					3.8			3.8
	Linear+Med						4	5		7	7					5.8			5.8
	Linear+Med+mask						2	7		6	6					5.2			5.2
	Linear+Iter						5	9		3	3					5.0			5.0
	Linear+Iter+mask						7	8		2	2					4.8			4.8
	Linear+KNN							6								6.0			6.0
	Linear+KNN+mask							3								3.0			3.0
Average	Boosted trees+MIA	**1.0**	**1.0**	**1.0**	**1.0**	**1.0**	**1.0**	**2.5**	**1.0**	5.0	**1.0**	**1.0**	**1.0**	**1.0**	**1.0**	**2.1**	**1.0**	**1.0**	**1.3**
	Linear+Mean	4.0	6.0	4.5	3.5	6.0	6.2	3.5	7.0	4.5	5.8	5.0	6.7	6.0	4.8	5.4	5.8	6.0	5.5
	Linear+Mean+mask	5.5	4.0	3.0	5.0	4.0	4.5	3.2	3.7	3.8	4.2	6.0	4.7	2.0	4.3	3.9	5.3	2.0	3.9
	Linear+Med	7.5	8.5	5.5	5.0	5.0	4.5	4.8	8.0	6.5	6.2	4.7	3.0	7.5	6.3	6.0	3.8	7.5	5.9
	Linear+Med+mask	6.5	6.0	5.0	4.0	7.0	3.0	4.5	3.3	4.8	5.8	4.3	2.0	3.0	5.7	4.3	3.2	3.0	4.0
	Linear+Iter	5.0	4.5	5.5	8.5	3.0	4.5	6.5	9.0	**1.8**	2.8	3.7	7.3	8.0	5.3	4.9	5.5	8.0	5.9
	Linear+Iter+mask	9.0	2.0	5.5	8.0	2.0	4.2	6.5	5.0	1.8	2.2	3.3	5.7	4.5	5.3	4.0	4.5	4.5	4.6
	Linear+KNN	2.5	7.5	8.0	5.0	9.0	9.0	6.5	6.0	9.0	8.3	9.0	7.7	8.5	6.4	7.8	8.3	8.5	7.8
	Linear+KNN+mask	4.0	5.5	7.0	5.0	8.0	8.0	7.0	2.0	8.0	8.5	8.0	7.0	4.5	5.9	6.7	7.5	4.5	6.2

Response to reviewers Click here to access/download;Personal Cover;response_to_reviewers.pdf

Response to Reviewers

Alexandre Perez-Lebel, Gaël Varoquaux, Marine Le Morvan, Julie Josse, Jean-Baptiste Poline

We sincerely thank the reviewers for their time and feedback. This feedback led us to add significant material to the manuscript, and we believe that the resulting findings are interesting and add value to the study. Below we reply to the various points raised by the reviewers, quoting the questions and suggestions, and provide answers to complement and explain the changes that we made to the manuscript.

Response to Reviewer #1:

> *Many of the imputation methods used to compare to MIA aren't commonly used in practice, such as what's called here "constant imputation", using the mean or median across all of a measured feature to substitute for those missing. Also, the current standard in the field (at least for inference) is multiple imputation, which doesn't appear to have been used. It's not clear if this is one of the methods described as "conditional imputation", but it would make sense to include this method, given its wide use, despite the computational burden.*

This comment echoes comment #2 of the other reviewer. Thus for this important question, we kindly ask the reviewer to look at our answer to comment #2 of the other reviewer.

> *In figure 1, MIA appears favorable at n=100000, but not so much at n=25000 or n=10000. Can the authors explain this? The imputation methods with their evaluation metrics should be evaluated separately at each n, not just overall.*

The Wilcoxon one-sided signed-rank test given in Supplementary Figure 4 shows that MIA is significantly favorable at level 0.05 against all other non-bagged methods at size n=2500. The higher the number of samples, the less significant are the results because the less tasks are available (indeed, only a small number of our datasets have more than 100000 samples). As the reviewer pointed out in the next comment, this is not clear to the reader when looking at Figure 1 so we added the results of the Wilcoxon test directly on Figure 1, as explained in the next comment.

> *Since some tasks are only examined at certain n, the different n are not comparable although they are shown side by side. This is very misleading.*

As each bar represents the difference to the mean for a given task (and given n), the fact that there are different tasks for different n does not lead to a systematic difference. Yet, as the reviewer points out, an important difference is that statistical power is not the same across sizes due to the varying number of tasks available at each size. To make it clearer for the reader, we added the results of the Wilcoxon one-sided signed-rank test directly on the results figures (Figure 1 and Supplementary Figure 2). Methods which performed significantly poorer (resp. better) at level 0.05 are marked with "*" (resp. "*(>)") and "**" (resp. "**(>)") for Bonferroni-corrected levels. By doing so, the reader can qualify his or her analysis of the results by seeing that some results are or are not significant at the considered size.

> *It seems that the info in figure 1 is an aggregate across all the datasets and tasks that were completed. Can it be shown broken out by data and task? Or can it be tested whether there were differences across data and task? **The best method may vary by task**. And if there were one very difficult task it would likely not be well represented here. A difficult task might merit more careful imputation, or not. Even though these are shown in the appendix, the tables are very dense and painful to examine.*

The reviewer is correct that there is "no free lunch" in machine learning, and it is well known that there is no universally optimal predictor. Following the reviewer's advice, we added Supplementary Figure 7 to better visualize scores obtained by the methods across tasks, showing scores of Figure 1 broken out by task. By doing so, we can see how each method compares to each other and observe the variabilities of the methods across tasks.
Regarding difficulties of the tasks, the reviewer highlights an interesting point: performances of the methods may vary depending on the difficulty of the task. To investigate this point, we added Supplementary Figure 5 showing the rank of each method as a function of the mean score obtained by all the methods on the task. The mean score is taken here as a proxy of the difficulty of the task: we assume that easier (resp. difficult) tasks have a high (resp. low) mean score. Local regressions (LOWESS) curves are plotted to spot trends. This figure highlights interesting trends : for some methods, the relative performance does not depend much on the difficulty of the task while others have strong trends. We added a paragraph highlighting these trends next to Supplementary Figure 5 in the appendix.

> *I find the relative prediction score difficult to interpret, the actual quality of any prediction isn't recoverable. Also if most of these data are MNAR, as the authors suggest, there might be very little predictive accuracy for any of these methods.*
> *If the results were broken out by task, then more standard measures of prediction accuracy like the AUC could be used instead of the relative prediction score, which is hard to interpret. Regardless of which metric is used (relative prediction score or AUC or coefficient of determination), the variability should be shown somehow, not just the average.*

Supplementary Figure 7 added in response to the previous comment that shows scores broken out by task also partly answers this comment.
The reviewer raises the important point that for results to be relevant, the methods should have a reasonable predictive accuracy. As visible on Supplementary Table 7, and newly added Supplementary Figure 7, we were careful not to benchmark tasks with predictive accuracies close to chance. 5 of the 10 classification tasks have an AUC above 0.8, 9 are above 0.6 and one, the worst performer has an AUC of 0.59 which is poor but still way above chance. 2 of the 3 regression tasks have a R2 above 0.5 and one, the worst performer has a R2 of 0.17.
Variability can now be read on newly added Supplementary Figure 7. Absolute predictive accuracies can also be read in Supplementary Table 8a.

> *The description of the nested cross-validations is a bit confusing. What exactly is the outer cross validation that "averages the validations of the tuned model"? Do they mean averaging the predictions? This is unclear.*

We rephrased and added more details in the second paragraph of the "Experiment" section page 7.

> *Presumably the same cross-validation and training sets were used across methods, but this should be stated explicitly.*

We added a sentence at the end of the first paragraph of the "Experiment" section page 7 to clarify.

> *Judging from figure 5, there are many features incorporated that have 80% or more incompleteness. That is a very difficult situation for extracting value from these features, did the authors look at whether it is worth the effort to include such data?*

To measure the feature importance and its relation with the proportion of missing values in the features, we ran permutation feature importance for each task for the method MIA. Once the method is fitted, to evaluate the importance of a feature, we randomly permute this feature 10 times and each time compute the drop in score which is then averaged. The higher the score drop the more important is the feature. We added Supplementary Figure 8 in which we plotted the feature importance (in terms of average score drop) as a function of the proportion of missing values in the feature. We observe no marked association between the proportion of missing values in the feature and its importance, arguing that all features are important for the prediction, even the ones with a high proportion of missing values (e.g 80%).

Minor comments

> *Page 3- "exposed in the appendices"- should this be "described in the appendices"?*

Indeed, we fixed the typo.

> *Figure 1. Why aren't KNN and KNN+mask shown for n=100000?*

KNN and KNN+mask were intractable at n=100000 in terms of memory usage: KNN has a $O(n^2)$ memory footprint (with 64-bit floats, 80GB are required).
To make it clearer, we added this information in Figure 1 and in its caption.

Response to Reviewer #2:

Summary:
The paper provides a rigorous empirical evaluation of imputation methods and native support of missing values via decision trees for downstream prediction tasks (classification and regression) on large-scale health datasets. The strengths of the paper include its originality in evaluating methods on large-scale health data, whereas previous evaluations focus on smaller-scale benchmark datasets and a principled evaluation based on significance tests on the relative rank of methods. As discussed more fully below, the paper does not appropriately discuss in the introductory section previous empirical evaluations of imputation methods for supervised learning. The paper should address in the section entitled 'Limitations and further work' two limitations of the study: (1) the evaluation metric focuses on accuracy in individual predictions rather than the capturing the multivariate distributional features of the data; and (2) the evaluations do not capture uncertainty of imputing missing values conditional on the observed values.

We thank the reviewer for recognizing that a major strength of this work is its evaluation of methods on large scale and real-world health databases whereas previous works have focused on much smaller datasets. We truly believe that this is what makes this work different and useful for the community.

Major recommendations:

> 1. It is claimed in the abstract and introduction that there is little or no existing empirical evaluations of imputation methods on downstream predictive tasks. There are in fact several existing evaluations of imputation methods based on downstream predictive tasks:

- *Bertsimas et al. (2017, Journal of Machine Learning Research)*
- *Poulos and Valle, (2018, Applied Artificial Intelligence)*
- *J¨ager et al. (2021, Frontiers in Big Data)*

The first and third papers listed also evaluates in terms of imputation quality. The second paper compares different combinations of imputation methods and predictive models in terms of downstream predictive performance, focusing on classification tasks only. The authors should discuss in the introductory section the differences between these evaluations and their work. I suspect a major difference is that the present study focuses on larger-scale medical data with many weakly correlated features, whereas the previous studies use smaller-scale machine learning "benchmark" datasets with varying average correlations between features.

We thank the reviewer for pointing us to these works which we were unaware of. We have taken them into account in the revised version of our manuscript and cited them as prior works in the introduction. Indeed, one of the major differences between these works and ours is the type of data used for benchmarking. Some of these works use rather small scale benchmark datasets with simulated (or artificially perturbed) missing values while we use large scale health databases with 'real' missingness (i.e missing values already present in the real datasets). Another important difference with some of these works is that we consider predictive models directly handling missing values.

> 2. There are two additional limitations that should be discussed in 'Limitations and further work': the evaluations are based on a single 1 performance metric that focuses on the accuracy of individual predictions rather than (1) distributional features of multivariate data or (2) the repeating sampling properties of the imputation methods. Regarding the first limitation, Little and Rubin (2014) argue that imputation methods should be evaluated on their ability to preserve the distributional characteristics of the data. Regarding the second limitation, Rubin (1996) argues that comparing imputed values to a single ground truth value fails to account for the uncertainty in imputing the missing values conditional on the observed data. A working paper by Wang et al. (2021) demonstrates why relying on a single performance metric and a single dataset (rather than repeatedly drawn samples of a dataset) may be misleading.

The reviewer touches on two crucial points here, which we address below.

- The first point mentions that "*imputation methods should be evaluated on their ability to preserve the distributional characteristics of the data".* In our paper, we do not assess the imputation quality of the various imputation techniques used. However, this is intended as the goal of the paper is to assess different schemes to predict in the presence of missing values, which is measured in terms of regression performance and classification accuracy. In particular, assessing the ability of an imputation method to preserve the distributional characteristics of the data does not provide a proxy for assessing its quality in prediction settings. Indeed, it has been shown recently that Impute-then-Predict procedures are Bayes optimal whatever the missing data mechanism, and whatever the choice of imputation function (reference LeMorvan 2021 in the manuscript). In other words, if the train and test

sets are imputed using the same imputation functions, then a flexible predictor will provide optimal performances asymptotically. This result highlights the fact that "good" imputation (in terms of preservation of the moments of the distribution for example) is not necessary for good prediction, at least asymptotically. Moreover, it also highlights the fact that using Multiple Imputation to reflect the uncertainty in imputed values is not necessary either, which brings us to the second point. This is at odds with the results of Little and Rubin, because the settings of Little and Rubin are quite different: Little and Rubin formulate a probabilistic model of the fully-observed data and ask how to recover this probabilistic model in the presence of missing values. However, here 1) we make no hypotheses with regards to the form of a probabilistic model of the data 2) the best predictor in the presence of missing values may not be the same as that for fully-observed data (theoretical discussions of this last point are made in Josse 2019 and LeMorvan 2021).

- The second point mentions that using single imputation instead of Multiple Imputation results in a failure of taking into account the uncertainty in the imputed values. This is true and it is indeed important for inference purposes to use Multiple Imputation or Bootstrapping to capture the uncertainty in the estimates due to missing values. However, again, the theoretical result mentioned above highlights the fact that it is not necessary to reflect the uncertainty in the imputed values to obtain optimal prediction performances.

Since the problem of imputation or inference is much more studied than that of supervised learning with missing values, it is natural to wonder whether the good practices in inference are also the ones that should be used for predictions. We therefore added a discussion about these points in the paper (paragraph "Multiple Imputation" page 3 and first paragraph of the "Interpretation" section page 6).

Moreover, as the previously mentioned theoretical results are true asymptotically, the question of whether or not Multiple Imputation is beneficial in finite-samples regimes remains. Thus, following the question of the two reviewers, we did additional experiments with Multiple imputation in the context of predictions. One challenge is that the notion of Multiple Imputation is not clearly defined in the context of predictive models (supervised learning), because it needs to be applicable in an out-of-sample setting: applying the predictive models on new data. Theoretically, the only result that we are aware of for Multiple Imputation in the context of prediction requires access to an oracle predictor for fully observed data and is valid only in MAR (Josse 2019, theorem 3). A tool closely-related to Multiple Imputation is that of the Bootstrap, which is used to obtain variances and gauge uncertainty with complicated estimators, including for Multiple Imputation. In predictive modeling, Bootstrap is classically used in the context of "bagging": Bootstrap aggregating, where multiple predictors are learned on bootstrap replicates and the prediction is an average of the prediction of each predictor. From the practical side, Khan et al 2019 review and compare a number of methods for using the Bootstrap and Multiple Imputation in the context of Ensemble approaches. It comprises the following methods: Learning on an averaged version of a multiply imputed dataset, Bagging single imputations, Bagging multiple imputations, constructing ensembles based on predictors that were each learned on a version of a multiply imputed dataset, etc. Since it is neither possible, nor the goal of this paper to compare all the ways to use Multiple Imputation in the context of predictions instead of inference, we chose one option: Bagging single imputation.

We thus added 3 new methods in the main experiment : Iterative+Bagging, Iterative+mask+Bagging and MIA+Bagging. By seeing the improvement brought by the two first methods, we suspected it was rather the effect of the Bagging than the effect of Multiple Imputation. To assess this claim, we ran MIA+Bagging and got as good results as Iterative+mask+Bagging, which confirmed the claim (as shown in Figure 1). To be sure that it was not an effect of an implicit multiple imputation when using MIA+Bagging, we ran a fourth method

Mean+Mask+Bagging, which obtained as good results and confirmed the fact that the improvement was an effect of the Bagging and not the effect of Multiple Imputation. Supplementary Figure 9 in the supplementary materials best summarizes these findings.
Note that these methods were very costly and this addition increased our total CPU time from 12 000 hours to 500 000 CPU hours, i.e 1 CPU for 60 years, which is a strong limitation of this part of our study. Not every lab could have afforded to run such a study.

We thoroughly rewrote the findings paragraph to integrate the new results coming from the experiments with multiple imputation and bagging. The new experiments also increased the correction for multiple comparisons in some of the statistical tests, which led to some differences no longer being significant.

Minor recommendations

> 1. pg. 2, middle left: the list of references starting with Miotto et al. 2016, should be in parentheses

Fixed.

> 2. pg. 3, top left: clarify that missing values in the test set should be imputed with the training set mean or median

We added a sentence to clarify.

> 3. pg. 3, top right: clarify that the feature sets should be the same within each predictive task. The concern is that if the ANOVA variable selection procedure yields different feature sets within a task, it would bias the comparison between imputation methods.

We added a sentence to clarify.

> 4. pg. 4, top right: "train datasets" → "training datasets"

Fixed.

> 5. pg. 8, bottom right: more information on how to access protocols.io

We added a link to the protocol in the "Availability of supporting data and material" section and added a reference to this link in the "Experiment" section.

> 6. pg. 11, Table 6 caption: typo: "larger that"

Fixed.
