# Supplementary material for: Benchmarking missing-values approaches for predictive models on health databases
Source: Gigascience. 2022 Apr 15;11:giac013. doi: 10.1093/gigascience/giac013 (PMC9012100; doi:10.1093/gigascience/giac013)
Supplement: giac013_Response_to_Reviewer_Comments_Revision_1 [file giac013_response_to_reviewer_comments_revision_1.pdf]

Author's Response To Reviewer Comments

Close

Response to Reviewers
Alexandre Perez-Lebel, Gaël Varoquaux, Marine Le Morvan, Julie Josse, Jean-Baptiste Poline

We sincerely thank the reviewers for their time and feedback. This feedback led us to add significant material to the manuscript, and we believe that the resulting findings are interesting and add value to the study. Below we reply to the various points raised by the reviewers, quoting the questions and suggestions, and provide answers to complement and explain the changes that we made to the manuscript.

Response to Reviewer #1:

> Many of the imputation methods used to compare to MIA aren't commonly used in practice, such as what's called here "constant imputation", using the mean or median across all of a measured feature to substitute for those missing. Also, the current standard in the field (at least for inference) is multiple imputation, which doesn't appear to have been used. It's not clear if this is one of the methods described as "conditional imputation", but it would make sense to include this method, given its wide use, despite the computational burden.

This comment echoes comment #2 of the other reviewer. Thus for this important question, we kindly ask the reviewer to look at our answer to comment #2 of the other reviewer.

> In figure 1, MIA appears favorable at n=100000, but not so much at n=25000 or n=10000. Can the authors explain this? The imputation methods with their evaluation metrics should be evaluated separately at each n, not just overall.

The Wilcoxon one-sided signed-rank test given in Supplementary Figure 4 shows that MIA is significantly favorable at level 0.05 against all other non-bagged methods at size n=2500. The higher the number of samples, the less significant are the results because the less tasks are available (indeed, only a small number of our datasets have more than 100000 samples). As the reviewer pointed out in the next comment, this is not clear to the reader when looking at Figure 1 so we added the results of the Wilcoxon test directly on Figure 1, as explained in the next comment.

> Since some tasks are only examined at certain n, the different n are not comparable although they are shown side by side. This is very misleading.

As each bar represents the difference to the mean for a given task (and given n), the fact that there are different tasks for different n does not lead to a systematic difference. Yet, as the reviewer points out, an important difference is that statistical power is not the same across sizes due to the varying number of tasks available at each size. To make it clearer for the reader, we added the results of the Wilcoxon one-sided signed-rank test directly on the results figures (Figure 1 and Supplementary Figure 2). Methods which performed significantly poorer (resp. better) at level 0.05 are marked with "*" (resp. "*(>)") and "**" (resp. "**(>)") for Bonferroni-corrected levels. By doing so, the reader can qualify his or her analysis of the results by seeing that some results are or are not significant at the considered size.

> It seems that the info in figure 1 is an aggregate across all the datasets and tasks that were completed. Can it be shown broken out by data and task? Or can it be tested whether there were differences across data and task? The best method may vary by task. And if there were one very difficult task it would likely not be well represented here. A difficult task might merit more careful imputation, or not. Even though these are shown in the appendix, the tables are very dense and painful to examine.

The reviewer is correct that there is “no free lunch” in machine learning, and it is well known that there

is no universally optimal predictor. Following the reviewer's advice, we added Supplementary Figure 7 to better visualize scores obtained by the methods across tasks, showing scores of Figure 1 broken out by task. By doing so, we can see how each method compares to each other and observe the variabilities of the methods across tasks.
Regarding difficulties of the tasks, the reviewer highlights an interesting point: performances of the methods may vary depending on the difficulty of the task. To investigate this point, we added Supplementary Figure 5 showing the rank of each method as a function of the mean score obtained by all the methods on the task. The mean score is taken here as a proxy of the difficulty of the task: we assume that easier (resp. difficult) tasks have a high (resp. low) mean score. Local regressions (LOWESS) curves are plotted to spot trends. This figure highlights interesting trends : for some methods, the relative performance does not depend much on the difficulty of the task while others have strong trends. We added a paragraph highlighting these trends next to Supplementary Figure 5 in the appendix.

> I find the relative prediction score difficult to interpret, the actual quality of any prediction isn't recoverable. Also if most of these data are MNAR, as the authors suggest, there might be very little predictive accuracy for any of these methods.
If the results were broken out by task, then more standard measures of prediction accuracy like the AUC could be used instead of the relative prediction score, which is hard to interpret. Regardless of which metric is used (relative prediction score or AUC or coefficient of determination), the variability should be shown somehow, not just the average.

Supplementary Figure 7 added in response to the previous comment that shows scores broken out by task also partly answers this comment.
The reviewer raises the important point that for results to be relevant, the methods should have a reasonable predictive accuracy. As visible on Supplementary Table 7, and newly added Supplementary Figure 7, we were careful not to benchmark tasks with predictive accuracies close to chance. 5 of the 10 classification tasks have an AUC above 0.8, 9 are above 0.6 and one, the worst performer has an AUC of 0.59 which is poor but still way above chance. 2 of the 3 regression tasks have a R2 above 0.5 and one, the worst performer has a R2 of 0.17.
Variability can now be read on newly added Supplementary Figure 7. Absolute predictive accuracies can also be read in Supplementary Table 8a.

> The description of the nested cross-validations is a bit confusing. What exactly is the outer cross validation that "averages the validations of the tuned model"? Do they mean averaging the predictions? This is unclear.

We rephrased and added more details in the second paragraph of the "Experiment" section page 7.

> Presumably the same cross-validation and training sets were used across methods, but this should be stated explicitly.

We added a sentence at the end of the first paragraph of the "Experiment" section page 7 to clarify.

> Judging from figure 5, there are many features incorporated that have 80% or more incompleteness. That is a very difficult situation for extracting value from these features, did the authors look at whether it is worth the effort to include such data?

To measure the feature importance and its relation with the proportion of missing values in the features, we ran permutation feature importance for each task for the method MIA. Once the method is fitted, to evaluate the importance of a feature, we randomly permute this feature 10 times and each time compute the drop in score which is then averaged. The higher the score drop the more important is the feature. We added Supplementary Figure 8 in which we plotted the feature importance (in terms of average score drop) as a function of the proportion of missing values in the feature. We observe no marked association between the proportion of missing values in the feature and its importance, arguing that all features are important for the prediction, even the ones with a high proportion of missing values (e.g 80%).

Minor comments
> Page 3- "exposed in the appendices"- should this be "described in the appendices"?

Indeed, we fixed the typo.

> Figure 1. Why aren't KNN and KNN+mask shown for n=100000?

KNN and KNN+mask were intractable at n=100000 in terms of memory usage: KNN has a $O(n^2)$ memory footprint (with 64-bit floats, 80GB are required).
To make it clearer, we added this information in Figure 1 and in its caption.

Response to Reviewer #2:

Summary:
The paper provides a rigorous empirical evaluation of imputation methods and native support of missing values via decision trees for downstream prediction tasks (classification and regression) on large-scale health datasets. The strengths of the paper include its originality in evaluating methods on large-scale health data, whereas previous evaluations focus on smaller-scale benchmark datasets and a principled evaluation based on significance tests on the relative rank of methods. As discussed more fully below, the paper does not appropriately discuss in the introductory section previous empirical evaluations of imputation methods for supervised learning. The paper should address in the section entitled ‘Limitations and further work’ two limitations of the study: (1) the evaluation metric focuses on accuracy in individual predictions rather than the capturing the multivariate distributional features of the data; and (2) the evaluations do not capture uncertainty of imputing missing values conditional on the observed values.

We thank the reviewer for recognizing that a major strength of this work is its evaluation of methods on large scale and real-world health databases whereas previous works have focused on much smaller datasets. We truly believe that this is what makes this work different and useful for the community.

Major recommendations:
> 1. It is claimed in the abstract and introduction that there is little or no existing empirical evaluations of imputation methods on downstream predictive tasks. There are in fact several existing evaluations of imputation methods based on downstream predictive tasks:
• Bertsimas et al. (2017, Journal of Machine Learning Research)
• Poulos and Valle, (2018, Applied Artificial Intelligence)
• J¨ager et al. (2021, Frontiers in Big Data)
The first and third papers listed also evaluates in terms of imputation quality. The second paper compares different combinations of imputation methods and predictive models in terms of downstream predictive performance, focusing on classification tasks only. The authors should discuss in the introductory section the differences between these evaluations and their work. I suspect a major difference is that the present study focuses on larger-scale medical data with many weakly correlated features, whereas the previous studies use smaller-scale machine learning “benchmark” datasets with varying average correlations between features.

We thank the reviewer for pointing us to these works which we were unaware of. We have taken them into account in the revised version of our manuscript and cited them as prior works in the introduction. Indeed, one of the major differences between these works and ours is the type of data used for benchmarking. Some of these works use rather small scale benchmark datasets with simulated (or artificially perturbed) missing values while we use large scale health databases with ‘real’ missingness (i.e missing values already present in the real datasets). Another important difference with some of these works is that we consider predictive models directly handling missing values.

> 2. There are two additional limitations that should be discussed in ‘Limitations and further work’: the evaluations are based on a single 1 performance metric that focuses on the accuracy of individual predictions rather than (1) distributional features of multivariate data or (2) the repeating sampling properties of the imputation methods. Regarding the first limitation, Little and Rubin (2014) argue that imputation methods should be evaluated on their ability to preserve the distributional characteristics of the data. Regarding the second limitation, Rubin (1996) argues that comparing imputed values to a single ground truth value fails to account for the uncertainty in imputing the missing values conditional on the observed data. A working paper by Wang et al. (2021) demonstrates why relying on a single performance metric and a single dataset (rather than repeatedly drawn samples of a dataset) may be misleading.

The reviewer touches on two crucial points here, which we address below.
The first point mentions that "imputation methods should be evaluated on their ability to preserve the distributional characteristics of the data". In our paper, we do not assess the imputation quality of the various imputation techniques used. However, this is intended as the goal of the paper is to assess different schemes to predict in the presence of missing values, which is measured in terms of regression performance and classification accuracy. In particular, assessing the ability of an imputation method to preserve the distributional characteristics of the data does not provide a proxy for assessing its quality in prediction settings. Indeed, it has been shown recently that Impute-then-Predict procedures are Bayes optimal whatever the missing data mechanism, and whatever the choice of imputation function (reference LeMorvan 2021 in the manuscript). In other words, if the train and test sets are imputed using the same imputation functions, then a flexible predictor will provide optimal performances asymptotically. This result highlights the fact that "good" imputation (in terms of preservation of the moments of the distribution for example) is not necessary for good prediction, at least asymptotically. Moreover, it also highlights the fact that using Multiple Imputation to reflect the uncertainty in imputed values is not necessary either, which brings us to the second point. This is at odds with the results of Little and Rubin, because the settings of Little and Rubin are quite different: Little and Rubin formulate a probabilistic model of the fully-observed data and ask how to recover this probabilistic model in the presence of missing values. However, here 1) we make no hypotheses with regards to the form of a probabilistic model of the data 2) the best predictor in the presence of missing values may not be the same as that for fully-observed data (theoretical discussions of this last point are made in Josse 2019 and LeMorvan 2021).
The second point mentions that using single imputation instead of Multiple Imputation results in a failure of taking into account the uncertainty in the imputed values. This is true and it is indeed important for inference purposes to use Multiple Imputation or Bootstrapping to capture the uncertainty in the estimates due to missing values. However, again, the theoretical result mentioned above highlights the fact that it is not necessary to reflect the uncertainty in the imputed values to obtain optimal prediction performances.

Since the problem of imputation or inference is much more studied than that of supervised learning with missing values, it is natural to wonder whether the good practices in inference are also the ones that should be used for predictions. We therefore added a discussion about these points in the paper (paragraph "Multiple Imputation" page 3 and first paragraph of the "Interpretation" section page 6).

Moreover, as the previously mentioned theoretical results are true asymptotically, the question of whether or not Multiple Imputation is beneficial in finite-samples regimes remains. Thus, following the question of the two reviewers, we did additional experiments with Multiple imputation in the context of predictions. One challenge is that the notion of Multiple Imputation is not clearly defined in the context of predictive models (supervised learning), because it needs to be applicable in an out-of-sample setting: applying the predictive models on new data. Theoretically, the only result that we are aware of for Multiple Imputation in the context of prediction requires access to an oracle predictor for fully observed data and is valid only in MAR (Josse 2019, theorem 3). A tool closely-related to Multiple Imputation is that of the Bootstrap, which is used to obtain variances and gauge uncertainty with complicated estimators, including for Multiple Imputation. In predictive modeling, Bootstrap is classically used in the context of "bagging": Bootstrap aggregating, where multiple predictors are learned on bootstrap replicates and the prediction is an average of the prediction of each predictor. From the practical side, Khan et al 2019 review and compare a number of methods for using the Bootstrap and Multiple Imputation in the context of Ensemble approaches. It comprises the following methods: Learning on an averaged version of a multiply imputed dataset, Bagging single imputations, Bagging multiple imputations, constructing ensembles based on predictors that were each learned on a version of a multiply imputed dataset, etc. Since it is neither possible, nor the goal of this paper to compare all the ways to use Multiple Imputation in the context of predictions instead of inference, we chose one option: Bagging single imputation.

We thus added 3 new methods in the main experiment : Iterative+Bagging, Iterative+mask+Bagging and MIA+Bagging. By seeing the improvement brought by the two first methods, we suspected it was rather the effect of the Bagging than the effect of Multiple Imputation. To assess this claim, we ran MIA+Bagging and got as good results as Iterative+mask+Bagging, which confirmed the claim (as shown in Figure 1). To be sure that it was not an effect of an implicit multiple imputation when using MIA+Bagging, we ran a fourth method Mean+Mask+Bagging, which obtained as good results and confirmed the fact that the improvement was an effect of the Bagging and not the effect of Multiple Imputation. Supplementary Figure 9 in the supplementary materials best summarizes these findings.

Note that these methods were very costly and this addition increased our total CPU time from 12 000 hours to 500 000 CPU hours, i.e 1 CPU for 60 years, which is a strong limitation of this part of our study. Not every lab could have afforded to run such a study.

We thoroughly rewrote the findings paragraph to integrate the new results coming from the experiments with multiple imputation and bagging. The new experiments also increased the correction for multiple comparisons in some of the statistical tests, which led to some differences no longer being significant.

Minor recommendations
> 1. pg. 2, middle left: the list of references starting with Miotto et al. 2016, should be in parentheses

Fixed.

> 2. pg. 3, top left: clarify that missing values in the test set should be imputed with the training set mean or median

We added a sentence to clarify.

> 3. pg. 3, top right: clarify that the feature sets should be the same within each predictive task. The concern is that if the ANOVA variable selection procedure yields different feature sets within a task, it would bias the comparison between imputation methods.

We added a sentence to clarify.

> 4. pg. 4, top right: “train datasets” → “training datasets”

Fixed.

> 5. pg. 8, bottom right: more information on how to access protocols.io

We added a link to the protocol in the “Availability of supporting data and material” section and added a reference to this link in the “Experiment” section.

> 6. pg. 11, Table 6 caption: typo: “larger that”

Fixed.

Close
