# Supplementary material for: Benchmarking missing-values approaches for predictive models on health databases
Source: Gigascience. 2022 Apr 15;11:giac013. doi: 10.1093/gigascience/giac013 (PMC9012100; doi:10.1093/gigascience/giac013)
Supplement: giac013_Response_to_Reviewer_Comments_Revision_2 [file giac013_response_to_reviewer_comments_revision_2.pdf]

Author's Response To Reviewer Comments

Close

All requested changes have been made.

Close
