# Supplementary material for: Benchmarking missing-values approaches for predictive models on health databases
Source: Gigascience. 2022 Apr 15;11:giac013. doi: 10.1093/gigascience/giac013 (PMC9012100; doi:10.1093/gigascience/giac013)
Supplement: giac013_Reviewer_1_Report_Original_Submission — Jamie Perin -- 8/9/2021 Reviewed [file giac013_reviewer_1_report_original_submission.pdf]

Reviewer Report

Title: Benchmarking missing-values approaches for predictive models on health databases.

Version: Original Submission **Date:** 8/9/2021

Reviewer name: Jamie Perin

Reviewer Comments to Author:

This is an interesting paper about how different methods for handling missing data can impact predictions. There are several things that need to be addressed however.
Many of the imputation methods used to compare to MIA aren't commonly used in practice, such as what's called here "constant imputation", using the mean or median across all of a measured feature to substitute for those missing. Also, the current standard in the field (at least for inference) is multiple imputation, which doesn't appear to have been used. It's not clear if this is one of the methods described as "conditional imputation", but it would make sense to include this method, given its wide use, despite the computational burden.
In figure 1, MIA appears favorable at n=100000, but not so much at n=25000 or n=10000. Can the authors explain this? The imputation methods with their evaluation metrics should be evaluated separately at each n, not just overall.
Since some tasks are only examined at certain n, the different n are not comparable although they are shown side by side. This is very misleading.
It seems that the info in figure 1 is an aggregate across all the datasets and tasks that were completed. Can it be shown broken out by data and task? Or can it be tested whether there were differences across data and task? The best method may vary by task. And if there were one very difficult task it would likely not be well represented here. A difficult task might merit more careful imputation, or not. Even though these are shown in the appendix, the tables are very dense and painful to examine.
I find the relative prediction score difficult to interpret, the actual quality of any prediction isn't recoverable. Also if most of these data are MNAR, as the authors suggest, there might be very little predictive accuracy for any of these methods.
If the results were broken out by task, then more standard measures of prediction accuracy like the AUC could be used instead of the relative prediction score, which is hard to interpret. Regardless of which metric is used (relative prediction score or AUC or coefficient of determination), the variability should be shown somehow, not just the average.
The description of the nested cross-validations is a bit confusing. What exactly is the outer cross validation that "averages the validations of the tuned model"? Do they mean averaging the predictions? This is unclear.
Presumably the same cross-validation and training sets were used across methods, but this should be stated explicitly.
Judging from figure 5, there are many features incorporated that have 80% or more incompleteness. That is a very difficult situation for extracting value from these features, did the authors look at whether

it is worth the effort to include such data?
Minor comments
Page 3- "exposed in the appendices"- should this be "described in the appendices"?
Figure 1. Why aren't KNN and KNN+mask shown for n=100000?

Methods

Are the methods appropriate to the aims of the study, are they well described, and are necessary controls included? Choose an item.

Conclusions

Are the conclusions adequately supported by the data shown? Choose an item.

Reporting Standards

Does the manuscript adhere to the journal's guidelines on [minimum standards of reporting?]() Choose an item.

Choose an item.

Statistics

Are you able to assess all statistics in the manuscript, including the appropriateness of statistical tests used? Choose an item.

Quality of Written English

Please indicate the quality of language in the manuscript: Choose an item.

Declaration of Competing Interests

Please complete a declaration of competing interests, considering the following questions:

- Have you in the past five years received reimbursements, fees, funding, or salary from an organisation that may in any way gain or lose financially from the publication of this manuscript, either now or in the future?
- Do you hold any stocks or shares in an organisation that may in any way gain or lose financially from the publication of this manuscript, either now or in the future?
- Do you hold or are you currently applying for any patents relating to the content of the manuscript?
- Have you received reimbursements, fees, funding, or salary from an organization that holds or has applied for patents relating to the content of the manuscript?
- Do you have any other financial competing interests?

- Do you have any non-financial competing interests in relation to this paper?

If you can answer no to all of the above, write 'I declare that I have no competing interests' below. If your reply is yes to any, please give details below.

I declare that I have no competing interests.

I agree to the open peer review policy of the journal. I understand that my name will be included on my report to the authors and, if the manuscript is accepted for publication, my named report including any attachments I upload will be posted on the website along with the authors' responses. I agree for my report to be made available under an Open Access Creative Commons CC-BY license (http://creativecommons.org/licenses/by/4.0/). I understand that any comments which I do not wish to be included in my named report can be included as confidential comments to the editors, which will not be published.

Choose an item.

To further support our reviewers, we have joined with Publons, where you can gain additional credit to further highlight your hard work (see: https://publons.com/journal/530/gigascience). On publication of this paper, your review will be automatically added to Publons, you can then choose whether or not to claim your Publons credit. I understand this statement.

Yes Choose an item.
